# Rapid Review of Gender-Affirming Healthcare for Children and Adolescents: Evidence Synthesis (2021–2025) and Recommendations for South Africa

**DOI:** 10.21203/rs.3.rs-8253372/v1

**Published:** 2025-12-09

**Authors:** KL Dunkle, Ingrid Lynch, Kevin Adams, Pierre Brouard, Jenna-Lee de Beer-Procter, Robin Dyers, Landa Mabenge, Liberty Matthyse, Chris McLachlan, Sakhile Msweli, Marion Stevens, Francois W.D. Venter, Elma de Vries

**Affiliations:** 1 Centre for Sexualities, AIDS and Gender, University of Pretoria, Pretoria, South Africa; 2 Hubert Department of Global Health, Emory University, Atlanta, USA; 3 Critical Studies in Sexualities and Reproduction, Rhodes University, Makhanda, South Africa; 4 Division of Plastic, Reconstructive & Maxillo-Facial Surgery, University of Cape Town, Cape Town, South Africa; 5 Department of Psychology, Stellenbosch University, Cape Town, South Africa; 6 Division of Health Systems and Public Health, Department of Global Health, Faculty of Medicine and Health Sciences, Stellenbosch University, Cape Town, South Africa; 7 WHO-FIC Collaborating Centre, Burden of Disease Research Unit, South African Medical Research Council, Cape Town, South Africa; 8 Department of Health and Wellness, Western Cape Government, Cape Town, South Africa; 9 Division of Public Health Medicine, School of Public Health, University of Cape Town, Cape Town, South Africa; 10 Gender DynamiX, Cape Town, South Africa; 11 KwaZulu Natal Department of Health, Pietermaritzburg, South Africa; 12 Department of Psychology, University of South Africa, Pretoria, South Africa; 13 KwaZulu Natal Department of Health, Empangeni, South Africa; 14 Affiliate, SARChi Chair Gender and Politics, Department of Political Sciences, Stellenbosch University, South Africa; 15 Ezintsha, Faculty of Health Sciences, University of the Witwatersrand, Johannesburg, South Africa; 16 School of Medicine, Faculty of Health Sciences, Nelson Mandela University, Gqeberha, South Africa

**Keywords:** gender-affirming healthcare, transgender and gender-diverse, affirming

## Abstract

**Why we need this rapid review:**

In South Africa, transgender and gender-diverse (TGD) children and adolescents continue to navigate health systems shaped by deep inequalities, limited specialised services, and persistent stigma. *Here at home*, these young people too often move through environments marked by the legacies of apartheid, economic exclusion, uneven service delivery, and ongoing social prejudice. These layered forms of inequality shape how families, caregivers, teachers, and communities are able to support the young people they love.

At the same time, international debates about gender-affirming healthcare (GAHC) for youth have become increasingly polarised, often driven by narratives that do not reflect South African realities or the rights-based framework of our Constitution. Much of this global rhetoric arrives at our shores without acknowledgment of our country’s unique social fabric, woven from resilience, cultural diversity, and a deep collective commitment to justice and dignity, even as we continue healing from our past.

This rapid review brings together rigorous, peer-reviewed evidence published from 2021–2025 on GAHC for youth under the age of 18 to help us understand what works, what is safe, and what young people need. It builds on the 2021 GAHC Guideline supported by Southern African HIV Clinicians Society (SAHCS), which is South Africa’s current clinical framework for gender-affirming care, ensuring that our practices remain aligned with the best available evidence and our constitutional values.

It also serves a deeper purpose: it offers guidance to the parents, families, caregivers, educators, health-workers, faith leaders, and communities who are trying to walk alongside TGD children and adolescents with compassion and clarity, sometimes in the face of fear, uncertainty, or misinformation.

It reflects the combined expertise of a queer- and trans-led team committed to dignity, equity, and affirming care, which are values consistent with *Ubuntu, Batho Pele*, and the broader South African human-rights tradition. This is work rooted in the understanding that a young person does not grow or struggle alone; they grow in families, in communities, in classrooms, in clinics, and in the collective dreams we hold for a more just and caring society.

This review is therefore not only a scientific exercise. It is an act of accountability. An offering of care. A step toward ensuring that every young person in this country, regardless of gender identity, race, class, disability, or geography, is met with dignity, safety, and support. It is work that recognises our shared responsibility to build a South Africa where all children and youth can thrive.

**What the evidence shows:**

Across 200 peer-reviewed research articles, 29 systematic reviews and 4 rigorous technical reports included in this rapid review, one picture emerges clearly, a picture that resonates with common sense, lived experience, and the stories told by TGD youth across the country:

**When TGD young people receive gender-affirming care within supportive families, schools, communities, and clinical settings, they do better, emotionally, socially, and medically.:**

An affirming home, an accepting teacher, a safe clinic, or a supportive friend can dramatically reduce distress, depression, and feelings of isolation. Young people become more confident, more hopeful, and more connected to their communities. Their school attendance improves. Their relationships deepen. Their sense of belonging grows.

**Non-clinical delays, long waiting lists, service gaps, and administrative barriers worsen distress and mental-health outcomes.:**

Internationally, these structural barriers are consistently linked to poorer wellbeing for TGD youth. In South Africa, many delays have nothing to do with safety or clinical readiness, they stem from limited staffing, uneven provincial capacity, referral bottlenecks, and financial constraints. For young people, these delays are not neutral. They often result in worsening anxiety, deepened dysphoria, and increased risk of self-harm or suicidality.

**Puberty pausers and gender-affirming hormones work as expected when monitored by specialists.:**

Puberty pausers do not override who a child is; rather, they give young people *time*; time to breathe, time to grow, time to make developmentally appropriate decisions about their bodies without the pressure of unwanted pubertal changes. Puberty pausers and gender-affirming hormones produce expected and desired outcomes under specialist monitoring. Side effects are usually mild and reversible, and mental-health outcomes are mostly stable or improved.

**Surgery for young people under 18 is rare, almost all evidence relates to chest reconstruction for transmasculine youth.:**

These surgeries are not undertaken lightly, are generally only offered to older adolescents, and are far less common than some public discussions suggest. When masculinising chest surgery is offered, it shows low complication rates and high satisfaction. Many young people report significant improvements in body image, participation in daily life, and overall wellbeing.

**Policies matter.:**

Policies shape lives. They decide whether a young person can change their name at school, whether a clinic has clear protocols, whether there is protection against discrimination, or whether a family must fight through unnecessary red tape.

- **Restrictive laws** are consistently linked to increased distress, self-harm, and suicidality.
- **Protective laws** such as anti-discrimination policies and access to legal gender recognition improve mental health and safety.

In short: **Affirming environments promote healthier outcomes. Restrictive environments are linked to distress and harm**.

**A South African lens:**

Almost all of the global research on GAHC for youth comes from high-income countries, places with more specialised services, shorter waiting times, and stronger safety nets than those available to most South Africans. Yet the findings are still clear and relevant when interpreted through a local lens:

**South African youth often face added burdens.:**

Poverty, community violence, discrimination, school-based exclusion, xenophobia, racism, homophobia, and limited access to specialised care all intersect to shape the mental health of TGD young people. These realities amplify the need for safe, affirming services, they do not diminish it.

**Most medical schemes do not cover GAHC.:**

Even families with medical aid often face high out-of-pocket costs. For many, this makes care inaccessible, reinforcing historical patterns of inequity.

**Nodal disparities across provinces deepen inequality.:**

Some provinces have dedicated clinicians, while others rely on referral pathways that stretch across hundreds of kilometres. Rural youth often carry the heaviest burden, travelling long distances, missing school, or facing stigma when trying to access support.

**Our histories matter.:**

South Africa’s past left a legacy of fragmented health systems and unequal access. But it also left a legacy of resilience, community solidarity, and a collective instinct to protect our most vulnerable.

This makes affirming, timely, and coordinated care even more essential here, not less.

**Limitations of the evidence:**

The evidence base is growing, but not perfect, and it is important for families and communities to understand its limitations without misinterpreting them:

- Most studies are observational, meaning they reflect real-world experiences rather than controlled clinical trials.
- Follow-up periods are short, especially for adolescents whose needs evolve rapidly over time.
- Non-binary and neurodivergent youth remain underrepresented, even though they make up an important part of our community.
- Very few studies come from the Global South, including African nations, where cultural contexts, resource constraints, and support systems differ.
- Randomised trials are not feasible in this field, given the small population, inability to blind participants or prevent them from accessing related interventions, and the ethical concerns of withholding needed care.

These limitations reflect gaps and constraints in the global research landscape, not a lack of benefit. Instead, they highlight the importance of building a stronger African evidence base in the years ahead.

**Conclusion: What this means for South Africa:**

The findings are remarkably consistent: Gender-affirming healthcare is effective and life-enhancing for young people who want it, with established safety profiles under professional care.

This rapid review supports the continued implementation and strengthening of the SAHCS GAHC Guideline. It also calls for policies and services that uphold the constitutional rights, dignity, and humanity of TGD youth.

But beyond the science, there is a deeper message for us as a country: When a child is affirmed, they are more likely to stay in school, maintain strong family bonds, build a sense of belonging, and grow into adults who contribute meaningfully to their communities. When a child is denied care and support, we risk losing them to despair, to disconnection, or to preventable harm.

**The message is simple:**

When we affirm, young people thrive. When we delay or deny, they suffer.

This review is a reminder that every young person deserves care that sees them, respects them, and allows them to grow into who they truly are without fear. It is a call to parents, teachers, health professionals, faith leaders, policymakers, and community members to walk alongside our youth with compassion and clarity.

In the spirit of *Ubuntu*, we remember: A child’s wellbeing is never theirs alone. It is held in the hands of all of us.

## Technical summary

**Introduction:**

In a context of growing international controversy and rising ideological opposition to gender-affirming healthcare (GAHC) overseas, South African stakeholders require an up-to-date, locally grounded, evidence-informed assessment of the health outcomes associated with gender-affirming interventions for transgender and gender-diverse (TGD) youth. While most of the global debate is shaped by political and cultural dynamics of the Global North, South Africa must interpret emerging evidence through its own constitutional, historical, and socio-cultural commitments, including the enduring principles of *Ubuntu, Batho Pele*, and a public health tradition rooted in equity and collective wellbeing.

This Rapid Review, which was not prospectively registered, synthesises research indexed between January 2021 and August 2025 to evaluate whether new evidence supports updates or refinements to the 2021 GAHC Guideline supported by Southern African HIV Clinicians Society (SAHCS), which is South Africa’s current clinical framework for gender-affirming care, ensuring that our practices remain aligned with the best available evidence and our constitutional values. The review was conducted by a queer- and trans-led team positioned in South Africa whose methodological, clinical, advocacy, and lived-experience expertise shaped the interpretation of evidence through an equity, rights-based, and context-sensitive lens. This positionality strengthens rather than biases the review; it ensures that the analysis attends to the realities of unequal access, historical trauma, and structural barriers that characterise healthcare experiences for many young people in South Africa.

**Objectives:**

The review sought to:

- Synthesise empirical evidence (2021–2025) across psychosocial, endocrine, surgical, policy, and non-medical gender-affirming interventions for TGD youth under 18, with attention to both benefits and potential harms.
- Assess alignment with the 2021 SAHCS GAHC Guideline, identifying where new data support, challenge, refine, or expand existing recommendations.
- Interpret global findings within South Africa’s unique realities, including constitutional protections, historical inequities, health-system constraints, cultural diversity, and intersecting structural barriers such as poverty, violence, stigma, racism, sexism, xenophobia, and homophobia.

In doing so, the review aims to support clinicians, policy-makers, psychosocial providers, educators, civil society organisations, and families in providing care that is evidence-based, developmentally appropriate, and aligned with the rights and dignity of TGD youth.

**Methods:**

A rapid review approach was adopted, consistent with PRISMA 2020 and PRISMA-RR guidance, and adapted to balance rigour with timeliness. Searches spanning January 2021 to August 2025 were conducted across 12 databases via EBSCO Host (University of Pretoria), supplemented by searches of ClinicalTrials.gov and the ISRCTN registry and targeted searches for recent systematic reviews, with search date limits of 2021-01-01 to 2024-12-31 and 2025-01-01 to 2025-12-31. The review team used Rayyan for screening and applied streamlining methods appropriate for rapid-review designs without compromising transparency.

**Eligibility criteria:**

Included:

- Peer-reviewed primary studies (N ≥ 5) reporting psychosocial or physical health outcomes of interventions aimed at TGD youth (<18), including interventions involving family systems, caregivers, educators, or public policy.
- Systematic, scoping, and narrative reviews with transparent and reproducible search protocols.
- Grey literature systematic reviews meeting the same methodological standards.
- Studies reporting family-level outcomes that included data for TGD youth.

Excluded:

Commentaries, opinion pieces, editorials, case reports or case series with N < 5, reviews lacking reproducible search strategies, and studies that described the health status or health history of TGD youth without reporting any health or psychosocial outcomes from some type of intervention were excluded.

Across all eligible reports, any empirically assessed health or psychosocial outcomes were included, and all empirical designs (quantitative, qualitative, and mixed-methods) were eligible; no meta-analysis was conducted because of extreme heterogeneity in populations, interventions, and outcome measures, so findings were synthesised narratively by intervention domain.

**Data extraction and synthesis:**

Two custom Airtable extraction tools were used to extract data from primary studies and systematic reviews respectively, capturing study characteristics, populations, interventions, outcomes, and key results. Formal de novo risk-of-bias or certainty grading across all individual studies was not undertaken; instead, where available, existing methodological appraisals and certainty assessments from included systematic reviews were used qualitatively to inform interpretation. Findings from both types of report were then synthesised narratively across five domains:

1. Psychosocial interventions
2. Endocrine interventions
3. Surgical interventions
4. Non-medical gender-affirming practices
5. Policy and legal interventions

The synthesis prioritised clinical relevance, harms and benefits, and equity considerations, with explicit attention to implications for South Africa’s health systems, socio-economic landscape, and constitutional obligations. Partial financial support for three authors was provided by Gender DynamiX.

**Results:**

The final dataset comprised 200 primary studies, 29 academic systematic reviews, and four grey literature systematic reviews, covering psychosocial, endocrine, surgical, non-medical, and policy/legal interventions for TGD youth.

Psychosocial interventions:

- Affirming psychosocial interventions, including support for social transition, family involvement, and safer school environments are associated with meaningful reductions in distress, anxiety, and suicidality. Neurodiversity-informed approaches support engagement and wellbeing for neurodivergent TGD adolescents.
- Affirming interventions are linked to improvements in emotional regulation, resilience, sense of belonging, school participation and day-to-day functioning. No study reported harms arising from affirming psychosocial care.
- In contrast, practices that delay, withhold, or discourage affirmation, including identity-change efforts, are consistently associated with psychological harm.
- Psychosocial care is not ancillary; it is an essential pillar of gender-affirming care for TGD children and adolescents, particularly in contexts shaped by structural violence, poverty, stigma, and limited mental-health resources.

Endocrine interventions:

- Puberty pausing medication and gender-affirming hormone therapy (GAHT) produce expected and desired physiological outcomes under specialist monitoring.
- Adverse events are generally mild, reversible, and consistent with known paediatric endocrine profiles.
- Mental health outcomes ranged from neutral to improved, with evidence that timely access (as opposed to prolonged non-clinical delay) is associated with lower suicidality, improved mood, enhanced quality of life, and greater appearance congruence and functioning.
- Adolescents receiving endocrine care report high treatment satisfaction, high continuation into adulthood, and very low rates of regret.
- Menstrual suppression is a safe, effective, and highly valued component of care for TGD adolescents who menstruate, reducing pain and menstrual-related dysphoria and resulting in high amenorrhoea rates and strong satisfaction.
- Fertility preservation through gamete preservation appears feasible and generally safe for adolescents, although accessibility for patients who are interested is constrained by cost, procedural dysphoria, and timing of referral. These factors combined highlight the importance of early, developmentally appropriate fertility counselling.

Surgical interventions:

- Evidence for patients under age 18 is limited but highly consistent and focuses almost exclusively on masculinising chest reconstruction, which is the only gender-affirming surgery routinely accessed by adolescents internationally.
- Within multidisciplinary programmes, masculinising chest surgery shows very low complication and revision rates, with safety profiles favourably comparable to those seen in adult or cisgender groups undergoing analogous breast procedures.
- Psychosocial outcomes are positive in the short to medium term, including improved body image, reduced dysphoria, and increased participation in social, educational, and physical activities, alongside high levels of patient satisfaction.
- Regret is rare, even in the longer-term follow-up studies that are available.

Non-medical gender-affirming practices

- Evidence on non-medical practices such as binding, tucking, packing, and padding is limited but indicates that these practices help many TGD adolescents manage dysphoria and navigate daily life, particularly where access to medical care is limited.
- Packing and padding are low-risk, while discomfort from binding and tucking is common but typically manageable with safer materials, rest periods, and clear guidance on warning signs.
- Clinicians should be familiar with these practices and provide non-judgemental, practical advice, including exploration of options for medical GAHC when indicated.

Policy and legal interventions:

- Restrictive or hostile policy environments – including healthcare bans, administrative barriers, and exclusionary school policies – correlate with measurable increases in distress, self-harm, suicidality, social withdrawal, disrupted care, and family strain among TGD youth.
- Conversely, protective policy frameworks such as anti-discrimination regulations, legal gender recognition pathways, and inclusive school protocols are linked with improved mental-health outcomes, reduced risk behaviours, and enhanced wellbeing.
- These findings demonstrate that healthcare outcomes are shaped not only by clinical interventions but also by the broader social, legal and institutional conditions that enable or obstruct access to affirming care.

**Limitations:**

- The majority of studies were small and observational, constraining causal inference and precision.
- Short follow-up periods limit understanding of long-term outcomes.
- Samples seldom reflected South Africa’s population in terms of race, socio-economic status, disability, neurodiversity, and rural/urban distribution, and no eligible primary studies from the Global South or South Africa were identified.
- Variability in outcome measures reduced comparability across studies, and rapid-review streamlining (including single-reviewer screening for some stages and limited grey literature searching) may have led to some missed or delayed records.
- Despite these limitations, the consistency of findings across study designs, populations, and regions strengthens confidence in the overall conclusions.

**Interpretation and implications for South Africa:**

Clinical practice:

- Affirmation is central to safe and effective care, reflecting both clinical evidence and South Africa’s rights-based obligations.
- Multidisciplinary, coordinated teams that include supportive families improve outcomes across medical and psychosocial domains, and strengthen continuity and safety.
- Delays arising from policy and administrative barriers, resource shortages, or lack of trained providers worsen youth mental health and constitute avoidable harm; they should be reduced wherever possible.
- Psychosocial support, including that provided by families and caregivers, should be integrated across all aspects of care, including assessment, initiation of desired treatment, and ongoing follow-up, with supportive management of co-occurring mental-health conditions rather than their use as reasons to delay care.
- Menstrual suppression and fertility counselling should be offered proactively as part of comprehensive, developmentally appropriate, patient-centred sexual and reproductive health care when indicated or requested.
- Clinicians should adopt neurodiversity-informed approaches to meet the needs of TGD youth who are autistic, ADHD, or otherwise neurodivergent, recognising that neurodivergence is not a contraindication for GAHC.
- Surgical pathways require structured preparation, psychosocial support, and equitable access, with attention to the psychological impacts of long waitlists and provincial disparities in availability.
- Structured caregiver engagement is essential, as affirming families and caregivers are consistently linked with improved mental-health outcomes, sustained engagement in care, and enhanced daily functioning.

Policy and health systems:

- Restrictive policies including administrative hurdles, opaque referral structures, or exclusion from medical-aid benefits cause measurable harm.
- Protective policies strengthen wellbeing at population level by reducing distress and suicidality, supporting resilience, and lowering demand on mental-health and emergency services.
- Health systems must address stock-outs, long waiting lists, and uneven provincial distribution of expertise, as these system-level barriers shape treatment trajectories, contribute to avoidable distress, and leave many adolescents without feasible routes into care.
- Equitable financing mechanisms are needed; current medical-aid exclusions disproportionately harm economically marginalised families and entrench inequities across provinces, sectors, and socioeconomic groups.
- Financing reforms should support multidisciplinary teams; reliable access to puberty pausers, GAHT, and menstrual suppression within essential medicines frameworks; workforce development; and expanded public-sector surgical capacity. Routine monitoring of wait times and geographic service distribution is also essential.
- Strengthening adolescent-friendly services and integrated pathways aligns with national priorities in youth mental health, HIV/SRHR, and human-rights protections and enables many non-specialised components of gender-affirming care to be delivered within routine adolescent health and mental-health services.
- Timely access should be treated as a matter of health equity and cost-effective prevention, given the documented harms of delayed care and its downstream impacts on schooling, distress, and emergency mental-health utilisation.

**Conclusion:**

Findings from this rapid review demonstrate that gender-affirming healthcare for TGD youth is evidence-informed, improves wellbeing, prevents harm, and supports healthier developmental and mental-health trajectories when delivered within supportive social, familial, clinical, and policy environments. The review reinforces the foundations of the existing South African GAHC Guideline and identifies opportunities to strengthen its implementation within South Africa’s legal, historical, and health-system context. While most available studies are observational, this reflects ethical and methodological realities of paediatric research and interventions that cannot be ethically randomised or withheld; consistent findings across diverse settings provide compelling, real-world evidence of effectiveness and safety.

Ultimately, this work reflects South Africa’s constitutional values: dignity, equality, and the right to access healthcare without discrimination. South Africa has a longstanding tradition of protecting the marginalised, guided by principles of collective care and justice, and ensuring affirming, timely care for TGD youth is both clinically sound and a continuation of that legacy, and a commitment to safeguarding the wellbeing of every young person entrusted to collective care.

## 1. Introduction

### 1.1 Rationale for this rapid review

Transgender and gender-diverse (TGD) is an umbrella term that describes people whose current gender identities do not align with the legal sex they were assigned at birth. Adolescents in this group face some of the greatest barriers to accessing healthcare worldwide, even as growing evidence shows that timely gender-affirming interventions are effective to alleviate distress and improve well-being and quality of life (1). Gender-affirming healthcare (GAHC) encompasses a wide range of social, psychological, and medical interventions that support TGD people to live in accordance with their gender identity. Recognised by major medical and human rights bodies as essential to dignity, autonomy, and equitable access to healthcare, GAHC is a critical component of human rights and health systems (2).

For adolescents, GAHC may include family, community and psychosocial support; puberty-pausing medication; gender-affirming hormone therapy; and, in some older adolescents, surgical interventions. These interventions alleviate gender dysphoria, prevent the distress of unwanted pubertal changes, and promote psychosocial wellbeing and dignity (2). Evidence consistently suggests that timely access to puberty-pausing medication and gender-affirming hormone therapy reduces depression, anxiety, and suicidality among adolescents experiencing gender dysphoria. These interventions act through well-understood psychosocial and physiological mechanisms, by providing psychosocial affirmation, interrupting unwanted secondary sex characteristics and reducing distress associated with gender dysphoria. These are evidence-based treatments endorsed by a wide range of medical associations worldwide and reflected in multiple international standards of care (2–7). Withholding or delaying GAHC when indicated is not neutral; it risks preventable harm (8).

Over recent decades, global medical consensus on the necessity of GAHC has strengthened, alongside recognition of the need to expand access (9). Yet, in parts of the Global North – notably the US and UK – political opposition has intensified (10,11). Laws and policies restricting adolescent access to gender-affirming care have functioned as a leading edge of broader right-wing efforts to roll back transgender rights. These campaigns typically link bans or severe limits on gender-affirming care with measures to curtail legal gender recognition, restrict TGD students’ participation in education and sports, regulate bathroom and facility access, and suppress LGBTQ-inclusive curricula or public expression under the banner of “protecting children” or “gender ideology” (12). These regions have also become influential sources of misinformation and disinformation about GAHC, contributing to political polarisation and creating confusion among both providers and the public about the scientific evidence base (13,14).

Such developments have had international ripple effects, including within South Africa, where the spread of misinformation and disinformation originating overseas risks distorting the scientific evidence base and undermining clinical practice in ways that threaten access to care and undermine adolescents’ broader sexual and reproductive health and rights (15–17). At the same time, local access to adolescent GAHC remains constrained by entrenched social and economic inequalities, shortages of specialised services, and persistent stigma and discrimination (18).

South Africa’s constitutional, legislative and health-policy frameworks strongly protect the rights to dignity, equality, non-discrimination, autonomy and access to healthcare, as enshrined in Sections 9, 10, 12, and 27 of the Constitution of the Republic of South Africa, 1996 (19). These protections are reinforced for children and youth in particular. Section 129 of the Children’s Act 38 of 2005 (20) provides a clear consent framework for healthcare, while Section 28(2) of the Constitution affirms that a child’s best interests are of paramount importance in every matter concerning them. These commitments are part of an ongoing project to address our country’s long standing health disparities, rooted in the legacy of apartheid. Within this rights-based framework, multiple policies have sought to expand equitable access and address the needs of marginalized groups (21)Against this global and national landscape, the 2021 South African HIV Clinicians Society (SAHCS) GAHC Guideline provides nationally relevant, patient-centred, informed-consent-based clinical guidance.

However, since its publication in 2021, the global evidence base on GAHC for children and adolescents has expanded rapidly. This newly published research varies widely in methods, scope and quality, but has emerged almost entirely from the Global North. Alongside this accelerating publication of primary evidence, multiple systematic reviews (22–49), evidence synthesis (2,3,5–7,49 (50–53) and new international guidelines (2,3,5–7,54) have been released. With very limited and partial exception (2) all have emerged from the Global North, with research questions and conclusions most relevant to patients, providers, health systems and political debates in high-income countries.

Given this evolving landscape, South African stakeholders – including TGD youth, their families, caregivers, educators, mentors and clinicians – need a comprehensive, rigorous, transparent, and *locally-grounded* synthesis of this rapidly growing body of research. This rapid review therefore examines studies indexed from Jan 2021 to August 2025 across psychosocial, endocrine, surgical, nonmedical, and policy domains of GAHC for youth under 18 years of age. Its purpose is to assess the rapid expansion in the evidence base on GAHC for youth since the publication of the 2021 SAHC GAHC Guideline, identify areas of uncertainty, and determine whether new evidence warrants updates or refinements. By situating emerging findings within South African legal, ethical, and health-system realities, the review aims to support clinicians, policy-makers, young people, and families with accurate, locally relevant, evidence-informed recommendations.

### 1.2 Purpose and research questions

**The purpose of this rapid review is to synthesise recent evidence (indexed 01 Jan 2021 – 14 Aug 2025) on the health impact of interventions for TGD children and adolescents (<18 years), across all intervention types**, including psychosocial, endocrine, surgical, legal, policy, structural and other interventions. Our overall aim is to collate findings from recent empirical research **to inform evidence-based South African clinical practice and planned future updates to the SAHCS GAHC Guideline** (2021).

The review is guided by four broad research questions mapping areas of emerging evidence since 2021:

1. **Psychosocial and supportive interventions**: What is the emerging evidence on psychosocial interventions for TGD youth < 18, including different approaches to mental health care, peer or community supports, family and parental/caregiver support and/or lack of support, and school-based interventions? What outcomes are reported, including benefits and risks?
2. **Endocrine interventions**: What is the emerging evidence on endocrine interventions for TGD youth < 18, including puberty pausing medication, gender-affirming hormone therapy, and other endocrine therapy, including benefits and risks? What outcomes are reported, including benefits and risks?
3. **Surgical interventions**: What is the emerging evidence on gender-affirming surgeries for TGD youth < 18, including masculinising chest surgery and other relevant procedures? What outcomes are reported, including benefits and risks?
4. **Legal, policy, and structural environments**: What is the emerging evidence on the *health impacts for TGD youth* < *18* of legal, policy, systemic and/or structural interventions that address or affect GAHC for youth? What outcomes are reported, including benefits and risks?

## 2. Background

South Africa’s legal, clinical, and structural landscape creates both opportunities and challenges for delivering high-quality, rights-based GAHC to adolescents. Access remains uneven and public discourse highly polarised, making it essential that emerging evidence is interpreted within the realities of the local health system, constitutional commitments, and lived experiences of TGD youth. This section provides the contextual foundation for interpreting the review findings and understanding their relevance for national guideline development.

### 2.1. What is gender-affirming healthcare?

GAHC encompasses a broad set of interventions that may be combined or accessed separately, depending on the needs and goals of each young person. GAHC is not linear nor prescriptive: there is no single set of interventions that applies to everyone. A patient-centred approach emphasises that care is individualised, rights-based, and participatory, with providers working alongside adolescents and their families to support dignity, autonomy, and wellbeing (55). To ensure that care is both competent and affirming, delivery of GAHC requires qualified professionals with expertise in child and adolescent health, gender identity, and the assessment of capacity to consent (2).

Depending on individual needs and goals, components of GAHC for youth under the age of 18 may include (55):

- Social and structural affirmation: affirming families, schools, healthcare settings, faith communities, cultural spaces and workplaces; legal recognition (name/gender marker changes)
- Psychosocial care: individual, family, and/or peer-group counselling, safe spaces
- Endocrine care: puberty-pausing medications, gender-affirming hormone therapy (GAHT), fertility counselling/preservation
- Surgical care: procedures available in later adolescence or adulthood, including a wide range of possible masculinising or feminising procedures
- Voice and communication support: voice therapy, communication skills, affirming expression
- Gender affirming and age-appropriate sexual and reproductive health care: tailored contraception, menstrual suppression, STI prevention, pre-exposure prophylaxis (PrEP), post-exposure prophylaxis (PEP), and HIV counseling, testing and treatment provided in gender-affirming clinical contexts

Different components are relevant at different points across the life course. For pre-pubertal children, no medical interventions are indicated; care focuses on psychosocial and social-structural support (55). In adolescence, psychosocial support, social affirmation, puberty-pausing medication, and GAHT are often central, with fertility counselling as needed. Timely access is crucial, as the development of unwanted secondary sex characteristics can be distressing, intensify dysphoria, and necessitate future surgical interventions. Surgical interventions, if any, are usually sought in later adolescence or adulthood, but remain an important part of the broader spectrum of care (2).

GAHC emphasises that care must be tailored to the values, needs and priorities of each young person, while recognising that access is shaped by barriers such as geography, cost, provider availability, stigma, and discrimination (56,57). Ensuring equity of access is therefore essential for translating clinical standards into timely access for all TGD youth.

### 2.2. Rights-based and legal foundations of GAHC in South Africa

South Africa’s policy environment strongly affirms the rights of children and adolescents to access appropriate healthcare (see Table 1). The Constitution enshrines the rights to equality, dignity, and freedom from discrimination (Sections 9 and 10)(19) – protections that extend to gender identity and gender expression (58). Section 27 guarantees the right to access healthcare, while Section 28 affirms children’s rights to basic healthcare services and to have their best interests considered paramount (59). Inherent in these rights are principles of bodily autonomy and self-determination, affirming that all people have the right to make informed decisions about their own bodies and healthcare (59).

These constitutional provisions are reinforced by the Batho Pele principles, which require public services to be delivered with consultation, service standards, courtesy, access, information, openness, transparency, and redress (19). Together, they create a legal and ethical imperative for patient-centred care – care that is respectful, responsive, and grounded in the lived experiences of those most affected. In the case of GAHC, this means centring the voices and needs of TGD persons, including children and adolescents, who often face intersecting forms of marginalisation (60,61).

International and domestic legislative frameworks further support adolescent access to GAHC:

- The UN Convention on the Rights of the Child affirms children’s rights to the highest attainable standard of health, non-discrimination, and consideration of the child’s evolving capacities and best interests (62).
- The Children’s Act 38 of 2005 provides the legal framework for informed consent. A child over 12 may consent to medical treatment if sufficiently mature and capable of understanding benefits, risks, and implications; surgical treatment requires the child’s consent (where capable) and parental or legal guardian assistance (20).

South African health policy instruments further strengthen this foundation. The National Contraception and Fertility Planning Policy and Service Delivery Guidelines (2019) (21) promote differentiated, person-centred service delivery and emphasise coordination and equitable geographic distribution of services. Both include commitments to improving access to hormonal care for TGD people, including through decentralised service delivery. The broader National Adolescent and Youth Health Policy (2017) (63) embeds adolescent-friendly standards – confidentiality, respect, non-judgment, and accessibility – directly relevant to GAHC delivery.

These rights-based frameworks shape not only the availability of care but also how evidence should be interpreted and applied in the South African context. **Clinical guidelines for GAHC must be developed and implemented in ways that uphold constitutional commitments to dignity, equality, non-discrimination, and bodily autonomy**. This requires recognising the social determinants that shape TGD adolescents’ health, ensuring equitable access to services, and guarding against the misuse of evidence in ways that could perpetuate exclusion or harm.

### 2.3. South African guidelines and standards shaping youth GAHC

Clinical guidelines provide evidence-informed recommendations that support high-quality, equitable, and consistent care that upholds legal and ethical standards. In politically contested areas of healthcare – including GAHC – they are especially important for grounding practice in established clinical norms.

The SAHCS GAHC Guideline (2021) was developed through a participatory process involving providers, TGD clients, and civil society organisations (55). This process reflects a standard in health research and guideline development where meaningful patient and community involvement is recognised as critical to producing guidance that is patient-centred and contextually relevant (64). The guideline operates on an informed consent model, recognising TGD clients as capable decisionmakers and centring their autonomy in the healthcare process. It is culturally responsive and adopts a lifespan approach, providing tailored recommendations for children, adolescents, and adults, with adaptations for resource-constrained settings.

Values underpinning the guideline include affirmation, dignity, equity, inclusion, informed consent, Ubuntu, Batho Pele (“People First”), and a strength-based approach that recognises trans resilience. These principles are echoed in other local professional and civil society statements (see Box 1).

### 2.4. Systemic and structural barriers to equitable GAHC access

Although GAHC has been available in South Africa since the 1970s, access remains highly uneven (65,66). Services are concentrated in a small number of urban centres, and many are available only in the private sector. As a result, TGD people — especially those who are economically marginalised and living outside major cities — face long travel distances, high transport costs, and substantial out-of-pocket expenses. Many ultimately go without care (18,67).

These inequities reflect broader patterns of structural exclusion. TGD people in South Africa experience pervasive stigma, discrimination, and violence, including workplace exclusion, poverty, and lack of access to safe housing and basic services, with significant impacts on health and wellbeing (68–70). Within healthcare settings, discrimination, denial of care, and provider abuse remain common (67,71,72).

Public-sector provision is constrained by limited funding, long waiting lists, and shortages of specialised personnel (18,73). The inclusion of gender-affirming hormones on the Tertiary National Essential Medicines List in 2019 improved policy clarity, but restricted initiation to tertiary hospitals and specialist endocrinologists, substantially limiting practical access (74). While technical support and online training have enabled some general practitioners to prescribe hormones in lower-level facilities, coverage is uneven and far from universal (73). Following major cuts to United States foreign aid for key population services in early 2025, provision further weakened, leading to the closure of decentralised GAHT programmes that had expanded access for people outside urban hubs (75). These services had played a critical role in reducing barriers for TGD people living far from tertiary hospitals, particularly those outside major urban centres (76). Gender-affirming surgeries are offered at only a few tertiary hospitals, with waiting times often spanning decades (73).

Young people face all these barriers alongside age-specific obstacles: lack of parental support, dependency on caregivers for transport, household poverty, educational disruption and heightened risks of homelessness (18,77). School-based discrimination and bullying further undermine mental health and disrupt access to vital school-based referrals or support systems (61,70). Within an already limited service landscape, the Gender Identity Development Service at Red Cross War Memorial Children’s Hospital in Cape Town remains the only dedicated public resource for TGD children and adolescents, underscoring the scarcity of age-appropriate specialised care.

Private-sector GAHC offers faster access but is unaffordable for most. Only 15.8% of South Africans have medical aid (78) and most schemes exclude GAHC (73). This means that even those with medical aid often face high out-of-pocket costs, while the vast majority must rely on the public sector. As a result, only a small proportion of TGD people in South Africa have realistic access to the full range of GAHC services. These disparities reflect the deep socio-economic and racial inequalities that continue to shape healthcare access in the country.

### 2.5. Safeguarding youth GAHC evidence integrity

Research on adolescent GAHC has become highly politicised in several Global North contexts, underscoring the importance of transparent, rigorous evidence evaluation. In the United States and parts of Europe, restrictive legislation has been justified through selective citation practices, privileging studies that support predetermined positions, omitting higher-quality research that does not, or overstating findings from methodologically limited studies (14,79,80). These tactics resemble broader anti-science strategies used in other contested areas of health and science, including coordinated campaigns that have influenced policy debates in parts of Africa (17,81,82).

The risks of evidence distortion are particularly acute when evidence is misrepresented in clinical decision-making. In clinical medicine, guideline development is expected to follow established norms for evidence appraisal and decision-making. Because randomised trials with minors are ethically and practically constrained, high-quality observational and longitudinal research forms the backbone of adolescent healthcare evidence (83). Most paediatric and adolescent guidelines – including many unrelated to GAHC – make strong recommendations based on low-certainty evidence when the balance of benefits, harms, feasibility, and patient values is clear (84). This reflects the GRADE Evidence-to-Decision framework, in which patient and caregiver values, preferences, and priorities take precedence when evidence is uncertain, rather than serving as secondary considerations (85,86).

Safeguarding evidence integrity therefore requires:

- transparent documentation of how evidence is identified, evaluated, and weighted;
- accurate representation of study findings, limitations, and methodological constraints;
- consideration of local health-system realities and lived experience when interpreting the relevance and applicability of evidence.

Explicit use of these principles distinguishes rigorous guideline development from politicised policymaking, reduces the risk of hidden bias, and clarifies how evidence is prioritised in areas of uncertainty (84,87). They align closely with South Africa’s constitutional commitments to dignity, equality, non-discrimination, bodily autonomy, and access to healthcare. These same principles guide the methodological approach used in this rapid review. The methods applied to identify, appraise, and synthesise evidence on GAHC for people under 18 are outlined in the following section.

## 3. Methods

This review uses a rapid review design. Rapid reviews streamline elements of systematic review methodology such as search scope, screening, and data extraction while maintaining transparency and rigour (88). This approach was selected to generate timely, policy-relevant evidence, balancing methodological robustness with the need for actionable findings to support excellence in clinical practice and inform planned future updates to the GAHC guideline. To ensure that findings were robust to clinical needs from the perspective of both TGD community members and clinicians who care for TGD youth, we deliberately built a project team that included all of these stakeholders and knowledge users (89).

The initial conceptualisation of the review arose jointly from the SAHCS, as the holders of the current guideline; Gender Dynamix, a South Africa transgender advocacy organisation who partnered in developing the 2021 Guideline; and the Professional Association for Transgender Health South Africa (PATHSA), who did not exist at the time of the original guideline creation, but who now provide a professional development home for South African clinicians who care for TGD people. Prof KL Dunkle, an epidemiologist, and Dr Ingrid Lynch, a research psychologist, served as technical leads for the review process with active collaboration from clinical and community stakeholders and experts at all phases from the process (89). This rapid review was not prospectively registered in a public registry such as PROSPERO.

Due to the wide variety of interventions and outcomes included under the broad rubric of gender affirming health care, our team had no expectation of being able to perform any meta-analysis of other formal quantitative synthesis; we instead focus on narrative syntheses of findings. The review nonetheless adheres to PRISMA 2020 reporting standards (90), with streamlining adaptations consistent with interim published guidance for reporting of rapid reviews (91). All streamlining decisions were systematically documented and summarised in this section to ensure transparency and replicability.

### 3.1. Eligibility criteria

***Peer-reviewed studies*** indexed from 01 January 2021 to 14 August 2025 were included if they reported psychosocial and/or physical health outcomes of interventions for TGD youth < 18, or for family units *including* TGD youth. Each study was required to present data on at least five individuals or family units; case reports and case series of fewer than five participants were excluded.

Eligible studies assessed interventions or exposures at any level of the socioecological model (individual, family, institutional, policy/legal, sociocultural) and were ***required to include empirical health outcome data for TGD youth* < *18***. Interventions of interest encompassed all types of GAHC (evaluated alone or in overlap); support or lack of support from families and caregivers; interventions based in schools or other institutional environments; as well as laws, policies, and structural interventions intending to impact TGD youth. We also included published empirical assessments of non-affirming interventions: psychosocial interventions without gender affirmation (provided that outcomes specific to TGD youth were reported), “gender exploratory therapy” (therapy that typically views gender diversity as a result of trauma, mental illness, neurodivergence or similar factors), and SOGIE (sexual orientation, gender identity and expression) conversion practices.

Controls or comparators were not required. Outcomes of interest included any quantitative or qualitative measure of health or psychosocial impact. Descriptive epidemiological studies were excluded. Studies focusing on barriers or facilitators to access to GAHC were excluded unless the health impact of barriers/facilitators was *specifically* reported.

All empirical research designs (quantitative, qualitative, and mixed-methods) were eligible, including prospective, retrospective and cross-sectional research using all data sources, including *inter alia* medical records, registries, surveys, interviews, and focus groups.

***Systematic, scoping and narrative reviews*** were eligible if they matched the above criteria and also reported a fully transparent and reproducible search and screening protocol for the assessment of primary research sources (we did not require protocol pre-registration). Systematic reviews that incorporated participants over the age of 18 were included if (a) they included outcome reporting or synthesis of findings specific to youth under 18, or (b) more than 50% of the included studies had a mean participant age < 18. In cases of ambiguity regarding the latter criterion, we erred on the side of inclusion. We did not include reviews of reviews.

For **systematic reviews only**, we included grey literature reports that met our systematic review inclusion criteria. This decision was based on the emergence of multiple such reports in recent years and the desire to avoid duplication of effort in the field given scarce resources.

This rapid review could not define precise PICO(T) criteria due to the wide diversity of interventions and outcomes. However, inclusion criteria were aligned as closely as possible to these frameworks as shown below. (92)

Exclusion criteria:

- **Not empirical research**: Specifically excluded were reviews without a published and reproducible search and screening protocol, summaries, commentaries, think pieces, editorials, and opinions.
- **Population not relevant**: Studies without data on health outcome for TGD youth under 18 excluded. Proxy data from families or HCPs permitted. For studies of SOGIESC youth, separately analysed data on at least five TGD youth must be presented.
- **Insufficient sample size**: Case reports or case series with N < 5 excluded. Composite or hypothetical examples not counted towards N.

### 3.2. Search strategy, screening and selection

#### Databases used

Searches were conducted via EBSCO Host at the University of Pretoria, with the following databases included:

1. Academic Search Complete
2. Africa-Wide Information
3. APA PsycArticles
4. APA PsycInfo
5. CINAHL
6. ERIC
7. Family & Society Studies Worldwide
8. Health Source - Consumer Edition
9. Health Source: Nursing/Academic Edition
10. MEDLINE
11. Social Work Abstracts
12. SPORTDiscus with Full Text

#### Search string

The overall search strategy was to combine [any term for trans and gender diverse identity] AND [any term for youth under 18] AND [any term for interventions related to gender identity].

To ensure that all potentially relevant evidence was captured, we specifically included terms for TGD identities considered outdated, pathologising, insulting, or offensive within the community, provided they could plausibly be used in peer-reviewed publications. Likewise, we included terms for approaches, philosophies, and intervention strategies deemed pathologising, demeaning, harmful, or abusive by members of the TGD community.

Our final search string used thus combined the following terms:

**Table:** 

Domain	Search Terms *(Caution: includes pathologising language, see above for rationale)*
**TGD identities**	Transgender* OR Transsexual* OR Transmen OR Transman OR Transwomen OR Transwoman OR “Trans men” OR “Trans man” OR “Trans women” OR “Trans woman” OR Transmasculine OR Transfeminine OR “Gender dysphoria” OR “Gender identity disorder” OR “Gender incongruence” OR “Gender nonconforming” OR “Gender euphoria” OR Nonbinary OR “Non binary” OR “Gender diverse” OR “Gender expansive” OR “Gender expansiveness” OR Agender OR Genderqueer OR “Gender queer” OR “Gender fluid” OR “Gender creative” OR Pangender OR “Two-Spirit” OR “2 Spirit” OR Bigender OR Demiboy OR Demigirl OR Genderflux OR Gendervariant OR “Gender variant” OR “Third gender” OR Androgynous OR Intergender OR “Gender questioning” OR AMAB OR AFAB OR ASAB OR “Cross-dresser” OR “Social contagion” OR “Rapid-onset gender dysphoria” OR “ROGD” OR Autogynephilia OR “De-transition” OR Detransition* OR Retransition OR “Gender confusion” OR “Gender distress”
**Youth/Age terms**	Child* OR Youth OR Young OR Adolescent* OR Adolescence OR Toddler OR Preschool* OR Tween OR Teen* OR Pediatric OR Pubescent OR Prepubescent OR “Post-pubescent” OR postpubescent
**Interventions**	Masculiniz* OR Masculinis* OR Feminiz* OR Feminise OR Feminising OR “Gender affirming” OR “Gender affirmation” OR “Gender confirming” OR “Gender confirmation” OR “Gender reassignment” OR “Sex reassignment” OR “Transition related care” OR “Transition-related care” OR “Gender affirming Hormone Therapy” OR GAHT OR “Cross sex hormones” OR “Hormone Replacement Therapy” OR “Hormonal Replacement Therapy” OR HRT OR “Hormone blockers” OR “Puberty blockade” OR “Pubertal blockade” OR “Puberty blockers” OR “Pubertal blockers” OR “Puberty delay” OR “Pubertal delay” OR Estrogen OR Estradiol OR Antiandrogens OR Spironolactone OR “5-alpha reductase inhibitor” OR “5-alpha reductase inhibitors” OR “5-alpha reductase inhibition” OR Finasteride OR Dutasteride OR Cyproterone OR Progestogen* OR Progesterone* OR Medroxyprogesterone OR Testosterone OR “Histrelin acetate” OR Leuprolide OR “GnRH analogue” OR “GnRH analogues” OR “Menstrual suppression” OR “Top surgery” OR “Bottom surgery” OR Vaginoplasty OR Vulvoplasty OR Phalloplasty OR “Penile Implant” OR Metoidioplasty OR “Clitoral release” OR Urethroplasty OR “Urethral lengthening” OR Scrotoplasty OR Glansplasty OR “Glans implant” OR Thyrochondroplasty OR “Facial Feminization” OR “Facial Feminisation” OR “Tracheal Shave” OR Vaginectomy OR Hysterectomy OR Oophorectomy OR Ovariectomy OR Salpingectomy OR Mastectomy OR “Breast reduction” OR Mammoplasty OR “Breast augmentation” OR “Chest augmentation” OR “Chest reconstruction” OR “Genital Reconstruction” OR Penectomy OR Orchiectomy OR Labiaplasty OR Clitoroplasty OR “Social transition” OR “Name change” OR “Gender marker” OR “Sex marker” OR “Legal transition” OR “Legal gender” OR “Legal sex” OR “Client-Centred” OR “Client Centred” OR “Client-Centered” OR “Client Centered” OR “Strengths-Based” OR “Family Acceptance” OR “Family Support” OR “Familial Support” OR “Family System” OR “Parental support” OR “Parent Support” OR Resilien* OR “Trauma-Informed” OR “Trauma Informed” OR Intersectional OR “Social Justice-Oriented” OR “Social Justice Oriented” OR “Minority Stress” OR WPATH OR “World Professional Association for Transgender Health” OR Psychodynam* OR Psychoanal* OR “Watchful waiting” OR “Gender exploratory therapy” OR “Gender critical therapy” OR “Reparative therapy” OR “Conversion therapy” OR “Neutral stance” OR “Identity-focused therapy” OR “Identity focused therapy” OR “Holistic therapy” OR “Child sterilisation” OR “Child sterilization” OR “Chemical castration” OR “Hormonal interference” OR “Hormone interference”

#### Dates of database searches

Multiple test searches on various proposed terminology lists were conducted in October and November 2024 to pilot search parameters and record retrieval procedures. An initial search using the above string was run on 2024-11-26 support development and pilot testing of appropriate inclusion and exclusion criteria. Following this first formal search, a decision was made to drop OpenDissertations and Teacher Reference Centre from the list of included databases to streamline screening, and final inclusion and exclusion criteria were agreed among the team. Second search for the 2024 round of screening was run on 2024-12-04. Both 2024 searches specified date restrictions of 2021-01-01 to 2024-12-31 which introduced a large number of duplicate records but ensured full coverage of the year 2024.

To accommodate the fast pace of research in the field, the search was re-run on 2025-08-14, with specified date restrictions of 2025-01-01 to 2025-12-31 (NB: dates after the search date are included as some articles are indexed online ahead of recorded publication dates).

#### Registry searches

In addition to database searches, ClinicalTrials.gov and the ISRCTN registry were searched in November 2024 using the same core concepts for transgender and gender-diverse youth and gender-affirming interventions, but no eligible registered trials involving TGD youth under 18 were identified.

#### LLM-assisted searches for previous systematic reviews

To supplement the database searches and ensure that other relevant systematic reviews, including grey literature sources, covering GAHC for youth conducted since 2021 were retrieved, we submitted the following prompt to ChatGPT and Perplexity research functions on 2025-09-12.

Search all online sources, including peer-reviewed and grey literature, for systematic reviews published from 2021 onwards that evaluate [DETAIL] Reviews must document a reproducible search process (e.g., PRISMA or equivalent), but need not be prospectively registered. If strictly pediatric-only reviews are limited, include systematic reviews that are broader as long as they report or extract data for minors (<18). Include all types of GAHT, any treatment length, and all outcomes (medical, psychological, social, quality of life, continuation/regret). Exclude non-systematic reviews and case series unless part of included reviews. Include grey literature (e.g., unindexed government/health authority summaries, agency reviews). Return all qualifying sources, and highlight those that report data or sub-analyses specifically for patients under 18.

The [DETAIL] block contained, in sequence:

- “any outcomes of gender-affirming endocrine interventions—specifically puberty blockers (GnRH agonists, progestins) and gender-affirming hormone therapy (GAHT: estrogen, testosterone, anti-androgens)—in patients under 18 years old, globally.”
- “any psychosocial interventions (excluding legal and policy reforms) aimed at improving outcomes for gender-diverse or transgender children and adolescents under 18 years old globally. Include reviews of family-based, school-based, individual, and group psychosocial interventions (e.g., supportive counseling, mental health therapies, peer support programs, family therapy, school-based inclusion efforts, teacher/staff training).”
- “any outcomes of any type of gender-affirming surgery in patients under 18 years old, globally. Include any surgical procedure (chest, genital, facial, or others), any length of follow-up, and all outcomes (medical, psychological, social, or quality of life).”
- “the impact of law or policy changes (at any level—local, regional, national, or international) on health outcomes for transgender or gender-diverse children and adolescents under 18 years old, globally. Include any review synthesizing evidence on the effects of anti-discrimination laws, healthcare access policies, education policy, insurance coverage, legal recognition, or other legal/policy measures on physical or mental health, well-being, safety, or healthcare utilization in minors.”
- “the outcomes of non–gender-affirming interventions for transgender, gender-diverse, or gender-distressed children and adolescents under 18 years old globally. Include reviews of interventions such as sexual orientation and gender identity/expression (SOGIE) change efforts, conversion therapy or practices, and “neutral exploratory therapy” approaches (i.e., approaches not affirming a trans identity or aiming to change or delay gender-related feelings/identity).”

LLM-suggested sources were screened by KLD to confirm they were actual literature reviews/evidence syntheses. Full text was retrieved where relevant and further screened by KLD and IL to verify whether the source met all eligibility criteria for systematic reviews.

#### Selection of retrieved sources for inclusion

Retrieved citations and abstracts were first uploaded into EndNote for initial deduplication. From EndNote, they were uploaded into Rayyan (rayyan.ai) for additional deduplication. Automated deduplication within Rayyan tools was used to batch-eliminate clearly ineligible sources types (theses, dissertation and all citations from newspapers and magazines).

After dual-screening approximately 300 abstracts from the search on 2024-11-26 to finalise and synchronise understanding of eligibility criteria, we proceeded with single-reviewer screening to accelerate the process (93). Initial screening was typically conducted by a single reviewer (usually KLD or EMdV for 2021–2024, and KLD or IL for 2025), with authority to exclude obviously ineligible sources. In alignment with acknowledged rapid review methods, we opted not to blind reviewers to each other’s ratings during screening; this compromise allowed us to streamline our processes in the face of time and resource constraints. This is an accepted compromise in rapid reviews that must prioritize efficiency over strict adherence to traditional systematic review protocols (94).

This first screening stage excluded only clearly ineligible sources, and any source not clearly ineligible was tagged as “Maybe” to ensure a second review and/or full text examination. For inclusion in the final dataset, a source had to receive at least two “Include” ratings at the abstract stage, with the “Include” rating sustained after detailed examination of the full text by two reviewers (KLD and IL). Disagreements between reviewers were resolved by discussion (co-authors participating in reviews included KLD, EMdV, IL, RD and RB).

These deviations from standard systematic-review methods (for example, single-reviewer screening, restricted grey literature searches) are the among the reasons this product is classified as a rapid review.

### 3.3. Data extraction and analysis

#### Data extraction tool development

Two distinct data extraction tools were developed in Airtable to accommodate the different evidence types in this review. For primary studies, an Airtable form was created by KLD and piloted by KLD and IL using 25 studies. This pilot phase refined coordination between reviewers, established quality assurance procedures, and optimized pre-coded dropdown fields for interventions and outcomes (93). A similar process using different data fields was used for the included systematic reviews. This approach recognized that systematic reviews required different data extraction fields focused on review methodology, included studies, and synthesized evidence rather than individual participant data (93).

#### Data extraction domains

##### Primary sources (Airtable form)

Data extraction for peer reviewed journal articles presenting original research followed a structured approach capturing information relevant to the research questions through dropdown menus, text fields, and checkbox selections:

- **Study characteristics:** DOI/PMID, authors, year, title, journal, abstract, country, study design (cross-sectional/observational cohort, case series, case-control, pre-post, controlled intervention, systematic review/meta-analysis, qualitative)
- **Population and recruitment:** Sample size, age characteristics, recruitment method (specialist gender clinic, other clinic, record review, registry cohort, population-based, community/convenience, internet, other), and diversity characteristics (autistic/neurodivergent, other disability/chronic illness, racial/ethnic minority, nonbinary explicitly mentioned, history of ACEs/violence, socio-economic/educational diversity, explicitly intersectional)
- **Interventions and exposures:** Comprehensive categorization including puberty pausers, gender-affirming hormones, surgery, social transition, individual therapy/counseling, peer group therapy, family support/lack thereof, social experiences, policy/legal factors, conversion practices, binding/tucking/packing, voice interventions, and other specified interventions
- **Outcomes and methodology:** Outcome types (gender dysphoria/body satisfaction, mental health, psychosocial functioning, physical health, adverse events, persistence/desistence/regret), outcome timing (baseline/cross-sectional, <1 year follow-up, ≥1 year follow-up, unclear), detailed outcome assessment methods, outcome assessor blinding, researcher positionality/reflexivity
- **Results:** Key quantitative results qualitative themes
- **Study context:** funding sources, conflicts of interest, and COI statements

##### Systematic reviews (Airtable form)

Data extraction for systematic followed a structured approach capturing information relevant to the research questions through dropdown menus and text fields:

- **Metadata and methods:** DOI/PMID and other identifiers, bibliographic details (authors, year, title, journal or report series), record type (academic or grey literature), protocol registration, provenance and commissioner, funding sources, conflicts of interest, number of included studies, countries represented, synthesis method (narrative review or meta-analysis), appraisal tools used, GRADE or other certainty summaries, and key methodological notes.
- **Review questions, eligibility and populations:** Study questions as formulated by each review, eligibility criteria for included reports, age eligibility thresholds, whether evidence for youth under 18 was separated or combined with adults, and the types of study designs and populations included.
- **Interventions and settings:** Intervention categories covered in each review (psychosocial and family support, puberty suppression, gender-affirming hormones, surgeries, policy and legal interventions, and other specified approaches), whether interventions were evaluated alone or as part of combined pathways, and any notes on service settings or delivery models.
- **Outcome domains and measures:** Outcomes synthesised across reviews, including suicidality and self-harm, mood, anxiety and distress, gender-related wellbeing and body image, family and peer relationships, broader psychosocial functioning and quality of life, bone health and growth, neurodevelopment, cardiometabolic health, fertility and sexual function, puberty progression, continuation and regret, adverse events and safety monitoring, and equity, access and service-delivery outcomes, together with the outcome measures or tools used.
- **Synthesised findings and certainty:** Brief main conclusions (typically one to two sentences) for each review, organised by intervention and outcome domain, any reported certainty or strength-of-evidence ratings, and methodological caveats such as risk of bias, indirectness, imprecision, or limitations related to setting and population coverage.

***Inclusion of original research reports in previous systematic reviews*** was handled as a distinct extraction domain for primary source within Airtable. We captured:

- Which primary studies from our original research report dataset were included in which systematic review.
- Any formal tools the previous review used to appraise those studies (for example, risk-ofbias instruments or GRADE-type approaches), and the certainty or quality ratings assigned to each relevant outcome where these were reported.
- How often each primary study appeared across our systematic review corpus, which helped identify reports that had been repeatedly synthesised versus those rarely or never included.

This information was used in two ways. First, because the rapid review design did not include a de novo, outcome-by-outcome certainty assessment, existing appraisals from prior reviews were treated as a secondary resource to inform interpretation of the underlying evidence. Second, cross-mapping of inclusions helped contextualise and avoid inadvertently over-weighting highly cited studies, by making patterns of repeated inclusion transparent.

#### Data extraction process

Data extraction from full-text documents of included primary research and systematic review sources was conducted by KLD and IL. Large language models (LLMs), primarily ChatGPT but also Perplexity, were used in a limited, supportive role in this process. A structured data-extraction prompt was applied to individual full-text PDFs to generate a plain text draft of source-specific data elements aligned with the Airtable fields; these outputs were then line-checked against the source articles by human reviewers (primarily KLD and IL), who corrected errors and manually entered final data into the extraction database. The LLMs were explicitly instructed to flag any data elements they could not locate or were uncertain about for targeted human review, and all such gaps were resolved using the original reports.

#### Analytic approach

Due to extreme heterogeneity in study designs, interventions, and outcomes, no meta-analysis was possible. Findings were therefore synthesised narratively, drawing directly on the structured Airtable extractions for primary studies and systematic reviews. The author team read full texts and extracted data iteratively, paying close attention to study design, sample characteristics, outcome measures, and any formal certainty ratings from existing reviews, so that patterns were interpreted with appropriate caution rather than simply counted.

#### Use of LLMs in analysis

The LLMs (ChatGPT and Perplexity) were used on an *ad hoc* basis to cross-check draft narrative descriptions of results against spreadsheets of the extracted data and PDF source material, helping to highlight possible omissions, internal inconsistencies, or misplaced details for human correction.

LLMs were not used for screening of retrieved sources, inclusion–exclusion decisions, formal data analysis, or to generate recommendations, conflict-of-interest statements, or contextual framing. Their roles were confined to fact-checking support, proofreading, and smoothing of wording, with all outputs treated as editable drafts. In all cases, information drawn from LLM-assisted drafts was reviewed, verified, and, quite often, rewritten by the human author team before inclusion in the final report.

### 3.4. Narrative synthesis of findings

Findings were organised across five primary domains of care – psychosocial, endocrine, surgical, non-medical, and policy interventions – with domain leads responsible for checking that narrative summaries accurately reflected the underlying data and highlighted important uncertainties. The synthesis prioritised practice-relevant findings and trends in relation to benefits, harms, and equity and access considerations, while explicitly noting where evidence was sparse, methodologically weak, or inconsistent.

Particular attention was paid to three cross-cutting interpretive lenses:

- **Global scope and local gaps**, focusing on post-2021 publications while highlighting areas where South African or broader LMIC-specific evidence remains absent.
- **Equity and context**, identifying where studies addressed, or failed to address, variation by geography, race/ethnicity, socioeconomic status, disability, or other axes of inequity.
- **Policy and practice relevance for South Africa**, framing outcomes in terms of their usefulness for clinicians, families, policymakers, and guideline developers, without overstating their potential for immediate revision.

To strengthen practice relevance while acknowledging the generally low–to–moderate certainty of much of the evidence, the synthesised findings were then assessed against the SAHCS GAHC Guideline (2021). For each domain, the team considered not only the direction and consistency of results, but also study quality, follow-up duration, and relevance to the South African context, to classify implications as:

- **Consistent** – emerging evidence aligns with current guidance and does not warrant changes to its scope or implementation.
- **Refine** – emerging evidence suggests current guidance could be sharpened or specified (for example, thresholds, subgroups, timing, dosing, monitoring, or implementation).
- **Challenge** – emerging evidence contradicts current guidance or introduces caveats that may require narrowing, modifying, or updating the recommendation.
- **New content area** – emerging evidence highlights an area not currently addressed for TGD youth, with potential for inclusion in future guidance.

## 4. Findings

The final dataset comprises 200 primary studies, 29 academic systematic reviews, and four grey-literature systematic reviews on gender-affirming care for TGD youth under 18, for a total of 233 retrieved sources.

The full PRISMA diagram on the next page shows how 7,560 database records (plus 59 records identified via LLM-assisted web searches and hand-searching reference lists of retrieved systematic reviews) were progressively narrowed through removal of duplicates, automated exclusions on ineligible publication types, and abstract and full-text screening to 233 included reports (90).

The full list of peer reviewed original research articles retrieved is given in the “Table A Full PRJ List” tab of the Data Appendix file, and the full list of systematic literature reviews with their extracted data is given in the “Table B Full SLR Data” tab of the Data Appendix file.

Overall, 109 out of our 200 retrieved primary articles (54.5%) had been described within at least one systematic review in our data set, with a range from 1 to 10 previous descriptions as shown in the histogram below. Conversely, this means that n=91 (45.5%) of the original research reports retrieved in our dataset have not been previously covered in systematic reviews on GAHC for youth under 18.


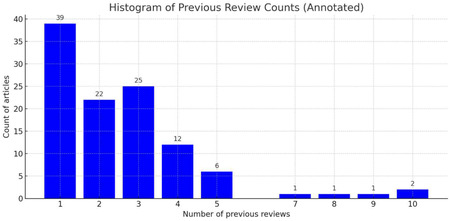


The third tab in the Data Appendix, “Table C PRJs mapped to SLRs” lists each of previously described sources mapped to the systematic review in which it appeared, along with the relevant review’s formal appraisal of the certainty of evidence, where this was done and locatable. Note that the certainty assessments were based on each systematic review’s research question(s) and assessment protocols, which resulted in varied assessments within articles that were reviewed multiple times.

The most highly reviewed articles were, unsurprisingly, all from 2021 or 2022:

- **10 previous reviews:** Carmichael P, Butler G, Masic U, Cole TJ, De Stavola BL, Davidson S, et al. Short-term outcomes of pubertal suppression in a selected cohort of 12- to 15-year-old young people with persistent gender dysphoria in the UK. *PLoS One*. **2021**;16(2):e0243894. doi:10.1371/journal.pone.0243894
- **10 previous reviews:** Tordoff DM, Wanta JW, Collin A, Stepney C, Inwards-Breland DJ, Ahrens K. Mental health outcomes in transgender and nonbinary youths receiving gender-affirming care. *JAMA Netw Open*. **2022**;5(2):e220978. doi:10.1001/jamanetworkopen.2022.0978
- **9 previous reviews:** Becker-Hebly I, Fahrenkrug S, Campion F, Richter-Appelt H, Schulte-Markwort M, Barkmann C. Psychosocial health in adolescents and young adults with gender dysphoria before and after gender-affirming medical interventions: A descriptive study from the Hamburg Gender Identity Service. *Eur Child Adolesc Psychiatry*. **2021**;30(11):1793–1803. doi:10.1007/s00787-020-01640-2
- **8 previous reviews:** Navabi B, Tang K, Khatchadourian K, Lawson ML. Pubertal suppression, bone mass, and body composition in youth with gender dysphoria. *Pediatrics*. **2021**;147(5):e2020039339. doi:10.1542/peds.2020-039339
- **7 previous reviews:** van der Loos MA, Hellinga I, Vlot MC, Klink DT, den Heijer M, Wiepjes CM. Development of hip bone geometry during gender-affirming hormone therapy in transgender adolescents resembles that of the experienced gender when pubertal suppression is started in early puberty. *J Bone Miner Res*. **2021**;36(7):1333–1343. doi:10.1002/jbmr.4262

In conducting the rest of our analyses, we treated prior systematic reviews as evidence sources in their own right and used them qualitatively to contextualise patterns and certainty of evidence in the primary studies; however, we took care to avoid inflating the influence of primary articles that appeared repeatedly across reviews. Our synthesis and conclusions are grounded in the totality of our source dataset, with insights and conclusions from prior reviews used to inform and enrich interpretation rather than to drive analyses.

### 4.1. Psychosocial support and care

Psychosocial care for TGD children and adolescents is outlined in the SAHCS GAHC Guideline (2021), particularly in Chapter 5 (Psychosocial Care) and Chapter 2 (Informed Consent). This rapid review considers psychosocial interventions that form part of the standard of care as well as proposed alternative interventions.

**SAHCS GAHC Guideline (2021):** The standard of care identifies psychosocial care as a central element of GAHC. Psychosocial care involves providing TGD children and adolescents with safe and affirming spaces, supporting social transition if desired, addressing mental health needs, and strengthening family and community support. The term mental health professionals (MHPs) refers broadly to providers whose scope of practice includes mental health or psychosocial support for TGD persons. MHPs accompany TGD clients on their gender-affirming journeys, ensuring that care is client-centred, affirming, and free of pathologisation.

The guideline highlights that children are most often brought for psychosocial care by parents or caregivers when a child expresses or is exploring a TGD identity, or when support for social transition is sought. Adolescents may access psychosocial care to manage heightened dysphoria at puberty, to seek support around social or medical transition, or as part of the informed consent process for puberty pausers or hormone therapy.

Across developmental stages, psychosocial interventions include supporting the process of self-understanding and identity formation, facilitating social transition where desired, and assisting adolescents in navigating stigma and minority stress. Family and community engagement are central, as supportive environments are consistently linked to improved outcomes. MHPs also play a critical role in informed consent processes for medical interventions, in working with schools and communities, and in addressing co-occurring mental health conditions without framing these as inherent to being TGD.

#### 4.1.1. Overview of the evidence base

This review identified 53 primary studies, 12 academic systematic reviews and four grey literature reports examining psychosocial aspects of care for TGD children and adolescents. The primary studies evidence base is methodologically heterogeneous and dominated by observational and qualitative designs, with the majority comprising cross-sectional surveys or retrospective clinic analyses (95–118). A substantial proportion of studies used qualitative or mixed-methods designs, drawing on interviews with young people, caregivers, families, or clinicians to explore experiences of support, identity development, access barriers, and care processes (103,119–131).

Only a small subset of studies comprised pilot or feasibility intervention evaluations, including group-based psychological programs, self-compassion courses, CBT groups, peer-support programmes, autism-informed sessions, and early-stage digital tools (120,132–137). Across the corpus, samples were typically small: most quantitative studies recruited between 30 and 200 participants, and most qualitative samples ranged from 5 to 40 participants (123,128,131). Ages most commonly ranged from 12–18 years, though some studies included younger children (3–11 years) (Durwood et al., 2021) or young adults recalling adolescent experiences (98,123,124).

Geographically, the literature is concentrated in high-income settings – primarily the United States, Canada, Australia, Aotearoa/New Zealand, and Western Europe (e.g., (95,99,104,105,107,131,132).

Recruitment was split between specialist gender clinics, often producing samples with higher baseline psychosocial need (e.g., (95,131,132), and community-based convenience samples, frequently online (e.g., (100,112,133). Intervention studies were generally small-scale, uncontrolled, or short-term, and experimental designs remain limited (132–135,137). Overall, the psychosocial evidence base is characterised by modest sample sizes, methodological diversity, and narrow geographic coverage, but provides a wide range of perspectives across clinical, community, family, school, and digital contexts.

We present the findings across four domains: social transition; family, peer, school, and community supports; psychological and digital interventions (including tailored approaches for neuro-diverse youth); and practices outside the recognised standard of care, followed by a synthesis of systematic reviews.

#### 4.1.2. Findings from primary studies

##### Social transition

Findings from multiple studies using diverse designs show that when TGD youth have their names, pronouns, identities, and forms of gender expression — such as clothing and documentation — affirmed, their overall wellbeing improves. Large-scale survey data show that feeling supported to affirm one’s gender — socially, legally, or medically — is associated with lower levels of suicidality, psychological distress, and anxiety, and with higher levels of happiness. However, nonbinary youth are the least likely to feel supported in these areas (100,138).

Qualitative research with children and adolescents similarly describes relief, joy, and better day-to-day functioning when youth are correctly named and gendered, and distress when misgendered or when social transition is delayed (102,103,116,125). In a large adolescent survey, greater perceived progress in social transition was linked to higher gender congruence, which in turn related to fewer symptoms of depression and anxiety (94). Prospective cohort and qualitative family-based evidence further underscore the psychosocial benefits of early social transition. In a longitudinal study of socially transitioned children, who on average transitioned around age 7, parent-reported anxiety and depressive symptoms decreased after transition, with no evidence of harm (139). Qualitative findings similarly show that early social transition improved mood, confidence, and social relationships, strengthened resilience, and produced immediate wellbeing gains for both children and their families (121).

Longitudinal cohort work focusing on identity development finds that most children who socially transition continue to identify as TGD several years later, with detransition and retransition uncommon and not reported as regretful in qualitative follow-up (140,141). Administrative cohort data from a paediatric service show that reidentification with birth-registered sex is rare overall and occurs mostly before or early in assessment, with very few cases after medical treatment (142). Community comparisons echo these findings: socially supported and socially transitioned youth report mental health within the typical range for their age, with only small differences in parent reports of anxiety compared to peers (143).

Qualitative studies with older adolescents and young adults add further nuance, showing that when detransition does occur it is typically shaped by contextual factors – such as limited support, invalidating care, or unsafe social environments – rather than regret about earlier social transition; many participants shifted to, or settled into, trans or nonbinary identities after discontinuing aspects of social and/or (mostly adult) medical transition, reflecting developmental fluidity rather than reversal (124). A companion study noted that public and clinical misrepresentations of detransition can heighten stigma and reduce access to support, underscoring the importance of non-pathologising care (124).

Some clinic-based analyses have reported poorer mental health among adolescents who have socially transitioned; however, closer examination suggests these patterns reflect contextual stressors (e.g., school harassment, strained peer/family relationships) rather than transition itself. In retrospective analyses, the apparent link between adolescent transition and suicidality disappears after adjusting for school harassment (115). In a clinic cohort, peer relations and family functioning – not social transition status – predicted psychological difficulties (113). Clinic-based comparisons likewise found no overall differences in self-esteem between socially transitioned and non-transitioned TGD children, with only minor domain-specific patterns emerging by sex assigned at birth. These likely reflect wider contextual factors rather than transition itself (117). A clinic-based study likewise found that social transition status alone did not predict mental health once peer and family factors were taken into account, underscoring the importance of contextual support in shaping wellbeing (108).

Alongside reductions in distress, social affirmation is also linked to gender euphoria and more expansive future orientation, highlighting that affirmation is generative – supporting flourishing and longer-term wellbeing (69,81).

Most studies are cross-sectional and concentrated in North America, and few follow participants beyond early adolescence, which limits causal inference and generalisability. Taken together, though, the evidence indicates that social transition in childhood and adolescence is associated with clear benefits and is not linked to worse mental health, while harms are more consistently tied to external stigma, harassment, and lack of family support rather than to transition itself.

##### Family, peer, school, and community supports

Parental affirmation and caregiver connectedness consistently emerge as among the strongest protective factors for TGD youth. In this literature, “family support” typically refers to affirming the young person’s gender identity and expression. Large-scale survey data show that such affirmation reduces risks of suicidality, with particularly strong protective effects for TGD and non-binary youth from racially marginalised groups in the US (109). Family support also buffers adolescents from the increased risks of suicide attempts and running away that often arise at key gender-identity milestones when families are unsupportive (96). Clinic-based and cohort studies also find that parental support is associated with lower rates of non-suicidal self-injury (110), fewer depressive symptoms and better overall health outcomes (95,104) and higher health-related quality of life and self-esteem (99)). Support from friends has similarly been linked to reduced anxiety and suicidality (110).

Cross-sectional community research further shows that parent support correlates with fewer depressive symptoms, lower disclosure stress, and reduced verbal and physical abuse, with some evidence that parental responses become more positive over time (101). In a large community cohort of socially transitioned children (ages 3–15), parent reports indicated that family and peer support were associated with fewer symptoms of anxiety and depression, and that peer and school support buffered the mental health impacts of victimisation (144). Even partial or evolving family support can have measurable benefits. A large community survey found that having at least one highly accepting adult or peer was associated with significantly lower odds of suicide attempts, with parental and peer acceptance providing the strongest protective effects (112).

Qualitative work highlights the complexity of family dynamics, showing that while children’s wellbeing improves with affirmation, parents may experience grief, ambivalence, or slower adjustment. Psychosocial interventions such as counselling, psychoeducation, and peer support can help parents process these emotions without delaying or undermining affirmation for the child, reinforcing the importance of youth-centred care that protects mental health while also strengthening family connection (121,125,129).

Other studies note that when families are unsupportive, adolescents often carry the burden of educating them, underscoring the value of structured counselling and psychoeducation to help families move toward affirmation (119,122,125,127). Clinical evidence also points to the role of practitioners in helping families adapt while maintaining support for their child throughout this process (129,145). Family dynamics also shape how adolescents experience care more broadly: supportive caregivers increase feelings of safety and confidence in healthcare decision-making, while rejecting or invalidating families intensify distress and suicide risk (51,64).

Schools are a particularly influential context for TGD youth. Survey evidence shows that greater school connectedness (i.e., feeling a sense of belonging at one’s school) is linked to fewer symptoms of depression and anxiety (111). Conversely, school-based victimisation is linked to markedly higher suicidality: in a large survey of TGD adults recalling their primary and secondary school years, those reporting school mistreatment had nearly double the odds of suicide attempts compared with those who did not (107). Qualitative studies likewise describe how affirming practices – correct pronoun use, supportive teachers, and access to appropriate bathrooms – improve engagement and foster belonging (103,114,116,146). Research in schools applying a minority stress framework found that TGD children often face discrimination, misgendering, and bullying, while family advocacy and peer connection helped buffer these stresses (147). TGD adolescents reported that affirming and knowledgeable school counsellors, and access to safe, confidential spaces at school, improved their wellbeing and engagement, while a lack of understanding from staff created mistrust and distress (106)

Beyond the school, family support, stable housing, and tolerant communities are associated with better health and lower substance use among TGD adolescents (104). Large surveys also point to inequities: nonbinary youth are less likely to feel supported to affirm their gender than binary peers (100).

Most available studies are cross-sectional, self-reported, and derived from high-income contexts, which limits causal inference and generalisability. Nonetheless, findings across family, school, and community contexts consistently show that supportive environments are protective, while unsupportive or hostile settings significantly increase risks of psychological distress, self-harm, and suicidality, as well as disengagement from education and care.

##### Psychological, digital, and tailored interventions

Evidence for affirming psychological and digital interventions is promising but remains methodologically limited. Pilot and open trials report encouraging effects: a resilience camp with fewer than 40 participants improved self-esteem and quality of life (118); a single-session triage program (FASST) offered to families while waiting for specialist services was linked to reduced depression and anxiety and better quality of life, with young people and families describing greater validation, hope, and support (132). Two pilots of mindful self-compassion interventions tailored for TGD adolescents also found significant mental-health benefits: An initial small study (N = 13) showed reductions in depression and anxiety, alongside gains in self-compassion and belonging that were maintained at three months (134). A subsequent larger open trial (N = 35) reported substantial reductions in suicidal ideation and depression, together with increases in self-compassion, that persisted at two-month follow-up (134). Separately, a co-designed cognitive behavioural therapy (CBT) group helped participants develop coping strategies for minority stress, with young people emphasising the importance of interim support while on long waitlists for medical care (120).

Digital interventions show mixed effectiveness. A pilot trial of a web app co-designed for LGBTQ+ youth improved coping skills and young people’s confidence in their ability to cope with minority stress, though it did not reduce overall symptoms more than the control group, and the sample was not TGD-exclusive (133). A nationwide rollout of the SPARX computerised CBT program found that TGD adolescents started with higher levels of depression, engaged less, and did not show the same improvements as their cisgender peers, suggesting that generic digital tools may fall short of meeting their needs (105). A trial of “It Gets Better” coping videos found a small, short-term reduction in suicidal thoughts among transgender and non-binary youth, particularly when they could identify with the narrators, but effects faded within weeks (137). Overall, emerging findings suggest that digital and psychological interventions are most effective when they reflect young people’s identities, use inclusive design, and sustain participants’ engagement over time (105,133,137).

Qualitative research shows that neurodivergent TGD youth, particularly adolescents with autism, value predictable, sensory-friendly environments, clear structure, and collaborative goal-setting in services. Such adaptations are not only therapeutic but also vital for ensuring that informed consent processes are participatory and accessible (128). A co-designed autism-informed group program with TGD youth and their parents identified practical strategies such as using role models, structured skills-building, and peer connection, all of which were highly valued (131). Similarly, a pilot of a peer-support group for TGD adolescents with autism found that parents reported improved wellbeing and reduced distress, while young people described becoming more aware and expressive about their gender identity (136).

Taken together, the literature suggests that affirming psychological and digital interventions can help reduce distress and support coping for TGD youth. While current evidence is small-scale and short-term, tailored and participatory designs appear most effective, generic digital tools may be less suitable, and brief media-based interventions have only temporary effects without sustained support. Importantly, no harms have been reported across available studies.

##### Practices outside the standard of care

A smaller body of research examines practices that fall outside recognised standards of care, including gender identity and expression change efforts (GIECE) (also referred to as “conversion therapy”). These are practices that aim, explicitly or implicitly, to discourage, suppress, or redirect a person’s affirmed gender identity or gender expression. They can include delaying or withholding affirmation while being framed as neutral observation or caution, as well as pathologising or gatekeeping approaches in assessment and care (148).

Across study designs, these practices are consistently associated with harm rather than benefit (98,102,124,149). Survey evidence links exposure to gender identity change efforts with substantially elevated risks of serious harm (98). TGD adolescents exposed to these practices were 55% more likely to attempt suicide and more than twice as likely to run away from home, with the most damaging effects observed when conversion therapy took place at younger ages (11–14) (98). These harms emerged rapidly and persisted and often intensified over time (98).

Qualitative studies describe how approaches framed as neutral – such as withholding affirmation entirely or requiring young people to “prove” the persistence of their identity before being affirmed – generates emotional distress, concealment, and strained family relationships (103,124,149).

We did not identify any primary studies supporting the claim that co-occurring mental-health or developmental conditions should be treated before – or instead of – providing gender-affirming healthcare (including in detransition-focused qualitative studies, which explicitly reported that gatekeeping did not prevent detransition and often eroded trust) (124). This approach falls outside the standard of care, which emphasises that treating co-occurring conditions is important but is not an alternative to gender-affirming care and does not address gender dysphoria itself (2).

Taken together, findings converge in the opposite direction from affirming interventions: non-affirming or pathologising practices increase risks of mental health deterioration, self-harm, and suicidality, while damaging trust within families and diminishing confidence in care systems.

#### 4.1.3. Systematic reviews of psychosocial evidence

Fourteen reviews synthesised psychosocial evidence for TGD children and adolescents: twelve academic journal articles (23,24,26,28,32–34,39,42,44,45,150) and three grey literature reports (50,51,53). Across all reviews, the underlying studies were limited by small samples, descriptive or cross-sectional designs, and geographic concentration in high-income countries.

##### Social transition

A systematic review of 11 studies assessing social transition in children and adolescents found strong evidence for the stability of early childhood transitions and the protective effects of chosen-name use among adolescents (44). Negative outcomes were attributable to contextual stigma, such as school harassment, rather than social transition itself. No study reported evidence of harm linked to social transition (44).

Reviews focusing on school-based environments similarly found that programmes and policies supporting TGD youth – such as student gender-sexuality alliances (GSAs), enumerated anti-bullying policies, inclusive curricula, affirming facilities, and visible institutional commitments to diversity – were associated with lower depression and suicidality, although implementation was uneven across study contexts (25,103).

##### Family, peer, school, and community supports

A systematic review of suicide prevention interventions for TGD children and adolescents found consistent evidence that family acceptance, affirming school climates, and access to gender-affirming medical care reduced suicidality, with no intervention-related harms reported (24). Reviews of mental-health correlates found that family connectedness, school safety, and peer affirmation were protective, whereas victimisation, discrimination, and isolation were associated with worsened depression, anxiety, and suicidality (33,38).

A metasummary of 31 qualitative studies examining parental responses to TGD children and adolescents found that affirming parents – particularly those who actively advocate in school and healthcare settings – were associated with improved emotional wellbeing and smoother access to care. Rejection or conditional support was linked to distress, secrecy, and disrupted healthcare access (42). Although experiential rather than interventional, the review highlighted parental support often strengthens over time with education and engagement, underscoring the clinical importance of psychoeducation and safety planning (42).

A systematic review of family-focused interventions identified seven programmes, including family therapy models, caregiver peer-support groups, and online psychoeducation (23). Despite heterogeneity in intervention design and outcome measures, findings showed a consistent direction of benefit: increased caregiver acceptance and affirming behaviours; improvements in youth depression, anxiety, and suicidality; and, in some cases, enhanced family communication. No studies reported harms (23).

Another review of 32 papers reporting on family-based interventions for TGD youth identified similar protective patterns (32). The review found no quantitative outcome trials of family therapy with TGD youth; evidence was primarily qualitative, observational, or derived from service evaluations. Across these studies, social affirmation and supportive relationships with families and peers were associated with anxiety and depression levels comparable to cisgender peers, and with psychosocial difficulties remaining below the clinical range. Access to family-based support services was also associated with substantially lower suicide attempts among TGD youth (32).

##### Psychological, digital, and tailored interventions

A review of 22 studies reporting on affirming psychological interventions for TGD youth and adults identified promising effects on depression, anxiety, resilience, and minority stress, although inconsistent and limited by methodological constraints (28). Among interventions specifically involving TGD adolescents, evidence came from two digital programmes and two group-based interventions. No harms were reported (28).

A systematic review of psychosocial interventions commissioned to inform the Cass Review identified six interventions for adolescents with gender dysphoria or incongruence, including CBT-based, skills-based, and multimodal therapeutic programmes (45). Outcomes generally improved or remained stable, and no intervention-related harms were reported. Study quality across interventions was low, with small samples and methodologically limited designs (45).

The grey-literature Evidence Check commissioned by the New South Wales Ministry of Health identified seven psychosocial interventions spanning psychotherapy, family therapy, crisis support, group CBT, web-based programmes, self-compassion training, and psychoeducation (50). Reported benefits included reductions in suicidality, depression, and anxiety. One RCT was identified, although findings were not disaggregated for TGD adolescents (50). Methodological limitations – including small samples, limited participant diversity, and reliance on mixed populations without subgroup analysis – constrained certainty. No harms were reported (50).

A grey-literature evidence brief commissioned by the New Zealand Ministry of Health synthesised six qualitative studies evaluating targeted mental-health and wellbeing interventions for adolescents (53)Interventions included online self-compassion training, clinic-based youth groups, multidisciplinary specialised care, a residential pride youth camp, and parent-focused programmes. Across studies, adolescents reported improved mood, reduced isolation, and better functioning, while parents described increased confidence and strengthened relationships (53). Effective interventions shared features such as safe spaces, body-kindness practices, peer support, and family involvement. Study quality ranged from high to very low, and all studies had small samples, with no reported harms (53).

A grey-literature rapid review by the RAND Corporation synthesised 22 psychosocial interventions for TGD children, adolescents, and young adults (51). Affirming interventions were associated with decreased suicidality and improved mood, although the evidence was rated as low certainty, largely due to small samples and variable study quality (51).

A systematic review focused on TGD youth with autism identified that neurodiversity-informed adaptations – such as sensory-friendly environments, structured sessions, and communication supports – were important facilitators of engagement and wellbeing. The evidence was descriptive and heterogeneous but directionally consistent (33).

##### Practices outside the standard of care

The grey-literature RAND review synthesised evidence on gender identity and expression change efforts (GIECE), identifying four relevant studies: three large retrospective community-based surveys and one case report (51). Across all studies, exposure to GIECE was associated with increased suicidal ideation and suicide attempts, with no evidence of benefit. Mental-health symptoms either worsened or remained unchanged. The overall certainty of evidence was rated low to very low, primarily due to retrospective designs, non-representative samples, and inconsistent definitions of GIECE (51).

The same review also identified seven case reports describing treatment of co-occurring mental-health or developmental conditions in TGD children and adolescents (51). The included cases described interventions targeting mental-health conditions, autism spectrum disorder, or both. Across reports, conventional treatment of co-occurring disorders alone did not lead to meaningful improvement and in some instances introduced additional risks (51). By contrast, when gender-affirming interventions were incorporated alongside conventional treatment, patients showed improvement. Evidence was rated as very low certainty, due to the exclusive use of case report designs (51).

Across systematic reviews, the evidence coheres with findings from primary studies: affirming psychosocial supports – including family acceptance, social transition, school inclusion, and tailored psychological interventions – are associated with improved mental-health outcomes and reduced suicidality. Conversely, non-affirming or pathologising practices, including GIECE, are associated with harm. Although certainty is limited by small samples, qualitative or observational designs, and methodological heterogeneity, the consistent direction of benefit across academic and grey-literature reviews – including two government-commissioned evidence briefs – strengthens confidence in these conclusions.

#### 4.1.4. Psychosocial care findings synthesis and guideline implications

Recent evidence on psychosocial interventions and support for TGD youth affirms the 2021 SAHCS recommendations and highlights several areas for refinement. Across domains of social transition, family and peer relationships, school and community environments, and tailored psychological, digital, and neurodiversity-focused supports, studies consistently show that affirmation is associated with improved mental health and wellbeing.

Affirming psychosocial practices – such as supporting a young person’s gender expression, strengthening family and peer connectedness, creating safer school environments, and providing tailored psychological or digital interventions – are linked to reduced depression, anxiety, and suicidality, as well as gains in resilience, belonging, and daily functioning. No study or systematic review has reported harms from affirming psychosocial care.

By contrast, non-standard or pathologising practices, including identity change efforts, enforced delays, or withholding affirmation, are consistently associated with adverse outcomes, such as heightened distress, self-harm, suicidality, and deteriorating family relationships. These findings reinforce that such practices fall outside recognised standards of ethical psychosocial care.

The strength of this literature lies in the consistency of findings across diverse study designs and contexts – even though most studies are cross-sectional, self-reported, and concentrated in high-income settings, with relatively few longitudinal or experimental designs. Nonetheless, findings converge to support psychosocial affirmation as both safe and beneficial, while underscoring the importance of supporting adolescents who face inconsistent or unsupportive family environments, addressing inequities affecting nonbinary youth and those from racially marginalised groups, and ensuring equitable access across diverse social and community settings.

Table 3 summarises the key implications for guideline development and highlights areas where recommendations may be refined.

### 4.2. Endocrine care

Gender-affirming endocrine care is addressed in Chapter 6 (“Hormone Therapy”) of the SAHCS GAHC Guideline (2021). This rapid review considers three main components of such care relevant to TGD adolescents: puberty-pausing medication, gender-affirming hormone therapy (GAHT), and the sequential or combined use of both. We also briefly consider emerging evidence on menstrual suppression as a gender-affirming intervention and on fertility counselling and preservation.

#### 4.2.1. Overview of the evidence base

Between 2021 and 2025, 117 original journal articles, 17 systematic reviews in academic journals and four grey literature reviews examined endocrine interventions for TGD adolescents. Most original research was observational cohorts or record reviews in specialist gender clinics in high-income settings, supplemented by a few registry and community-survey studies. Follow-up durations typically ranged from six months to several years. Study designs were predominantly non-randomised, with small to moderate samples and some overlapping cohorts. Measurement tools for mental health and wellbeing varied, and reporting of attrition was inconsistent. At the same time, longitudinal cohorts provided repeated measures across developmental, bone, and psychosocial domains, while registry data enhanced external validity for safety and utilisation outcomes.

Overall, this evidence base is sufficient to characterise short- to medium-term effectiveness and safety of puberty pausing medication and GAHT under specialist care. Remaining evidence gaps relate to long-term skeletal and cardiometabolic outcomes, generalisability beyond high-income clinic settings, and the limited visibility of non-binary and neurodivergent adolescents in reported outcomes. Very few studies capture adolescents’ own values, treatment goals, or decision-making needs, and new research that meaningfully incorporates youth voices is essential to support rights-based, person-centred care.

#### 4.2.2. Puberty-pausing medication

**SAHCS GAHC Guideline (2021):** Pausing puberty using gonadotropin-releasing hormone agonists (GnRHa) in GAHC aims to relieve gender dysphoria, prevent distress from unwanted pubertal changes, and support psychosocial wellbeing. No medical interventions are required for pre-pubertal TGD children. By pausing pubertal changes, suppression can ease acute distress and allow adolescents time to mature cognitively and emotionally before considering interventions with irreversible effects.

Pausing puberty is considered fully reversible within current evidence and builds on long-standing paediatric endocrine practice for precocious puberty and certain reproductive cancers in youth. The same medications are used to pause the development of secondary sex characteristics. Initiation should follow multidisciplinary assessment, informed consent, parental or caregiver involvement wherever possible, and oversight by a paediatric endocrinologist.

##### Findings from original research

Twenty-three original journal articles focused on puberty pausers for TGD adolescents. Most were observational cohorts embedded in specialist gender clinics in the US, Europe, and other high-income settings, often using retrospective record review alongside prospective follow-up (151–165).

One administrative dataset contributed population-level data (156) and one neuroimaging study compared adolescents on GnRHa with peers (166). The evidence base also includes one cross-sectional metabolic comparison of TGD adolescents and cisgender controls (167–169) and one qualitative study examining adolescent, parent, and clinician perspectives on puberty suppression(170).

Participants were typically in early to late puberty at initiation, usually Tanner stages 2–3 (154–156,158,171), with some studies enrolling adolescents in later pubertal stages (152,153,172). Most clinical cohorts enrolled between 40 and 100 adolescents, and a few larger record-based studies exceeded 100 participants. Follow-up ranged from 6 months to around 3 years for most cohorts (152,155,161), with some record-based studies capturing longer treatment courses (147,154).

Outcomes covered include physical and developmental outcomes (pubertal suppression, growth and bone health), mental health and psychosocial wellbeing, safety and adverse events, and treatment trajectories.

###### Physical and developmental outcomes

Across cohorts based on record review and prospective clinical studies, GnRHa consistently suppressed gonadotropins and sex-steroid levels to prepubertal ranges and halted pubertal progression, using both implant and depot formulations(152,154,158,165,172). Growth and height velocity generally slowed during suppression, reflecting the intended effect, with younger patients and those initiating at earlier Tanner stages experiencing less deviation from expected height trajectories than those starting later. Alternative androgen-blocking regimens were less common; one cohort of transfeminine adolescents treated with bicalutamide reported breast development in most patients within the first 7 months and no evidence of hepatotoxicity (Fuqua et al., 2024).

Bone mineral density (BMD) and bone mineral content generally increased with growth but declined relative to age-matched norms, with lumbar spine z-scores most affected. These reductions were typically transient, with recovery toward affirmed-gender reference ranges after discontinuation of GnRHa or initiation of GAHT (153,157,160,171). Registry data described expected endocrine profiles and normal pubertal resumption when treatment was stopped or transitioned to hormones (173). Neuroimaging work did not identify structural brain harm attributable to suppression (166).

###### Mental health and psychosocial wellbeing

Prospective cohorts using validated tools such as the Child Behavior Checklist (CBCL) and Youth Self-Report (YSR) generally found mental health to be stable or modestly improved over 12–36 months of GnRHa treatment (151). Service-based cohorts and cross-sectional comparisons reported reduced internalising and depressive symptoms, lower anxiety, and lower self-reported distress compared with baseline or untreated peers (127,155,156).

Research with more granular repeated measures identified clearer patterns: one prospective cohort conducted at a multidisciplinary gender clinic found significant improvements in depressive symptoms, anxiety, suicidality, internalising and externalising problems, and body uneasiness within months of initiating GnRHa, following an observed deterioration during the extended pre-treatment assessment period (172). Individual trajectories were heterogeneous, with many adolescents maintaining similar scores over time and smaller subsets showing improvement or deterioration on different scales (161). Cross-sectional clinic and community samples found overall psychosocial functioning and quality of life broadly comparable to population norms, with some areas of residual distress related to external stressors (163,164). Importantly, no study identified systematic worsening of mental health attributable to GnRHa treatment.

###### Safety and adverse events

Across studies, adverse events matched the expected safety profile of GnRHa in paediatric practice. Common side effects included hot flushes, fatigue, and headaches (151,158,159). Laboratory monitoring showed biochemical suppression without clinically significant hepatotoxicity, renal dysfunction, or haematological abnormalities (152,154,157,172). Where metabolic differences were observed in cross-sectional comparisons with cisgender controls (e.g., lower insulin sensitivity, higher HbA1c; (168), these reflected group differences rather than treatment-emergent pathology, and were not interpreted by study authors as clinically actionable abnormalities.

Bone-related concerns centred on transient BMD z-score declines, particularly in the lumbar spine, with evidence of catch-up after GnRHa discontinuation or subsequent GAHT (153,160,171). Serious adverse events were very infrequent; across all included studies, only one case of idiopathic intracranial hypertension was identified (174). No study identified a pattern of irreversible harm linked to GnRHa use in adolescents.

###### Treatment trajectories and patient experience

Longitudinal and mixed-method studies described puberty suppression as providing relief from pubertal distress, improved day-to-day functioning, and space to make informed decisions about future interventions (155,170). Adolescents and parents emphasised the value of halting unwanted development while retaining flexibility about subsequent GAHT (155,170). A retrospective cohort analysis found that adolescents initiating GnRHa are not more likely to continue on to gender-affirming hormones than those who were GnRHa-na ve (173).

Service evaluations reported high adherence to dosing schedules, successful implant replacements, and stable biochemical suppression over years of care (158,159). In an extended-duration implant study, about 16% of adolescents had hormone levels that suggested a partial return of puberty, although most did not show any physical pubertal changes (159). Cross-sectional work suggested that earlier suppression was associated with less distress in puberty-linked characteristics such as voice, and higher satisfaction with gender-related bodily attributes (163).

##### Findings from systematic reviews

Four systematic reviews in peer-reviewed journals synthesised evidence on puberty-pausing medication for adolescents: an academic critical review (35), a systematic review commissioned for the Cass Review (175) a SEGM-commissioned systematic review and meta-analysis (Miroshnychenko, Roldan, et al., 2025), and a grey literature evidence brief from the New Zealand Ministry of Health (53). The Cass Review-linked and SEGM-commissioned reviews were produced within highly contested policy debates in the UK and US respectively. These commissioning contexts shaped several methodological decisions – particularly the use of narrow inclusion criteria and lack of substantive input from TGD youth or communities into defining meaningful outcomes, framing the research questions or interpretation of the findings.

The critical review synthesised nine adolescent studies and reported consistent suppression of pubertal progression, transient reductions in bone mineral density, decreases in depressive symptoms and distress, improved peer relations, and stable or improved suicidality (36). Study quality varied widely, and follow-up was short (36).

The systematic review commissioned as part of the Cass report (50 studies initiated <18 years) found that puberty suppression reliably halted puberty and produced expected endocrine effects (175). Mental-health findings were mixed – small improvements in some cohorts and no change in others – with no evidence of psychological harm. Certainty was limited by small samples, non-comparative designs, and overlapping cohorts (175).

The SEGM-commissioned review included ten studies with up to 36 months of follow-up (48). Evidence suggesting possible small improvements in depression and functioning and minimal change in gender dysphoria was rated very low certainty. Meta-analysis confirmed reductions in bone-mineral-density z-scores. Certainty ratings were very low across domains due to confounding, co-interventions, and small sample sizes (48).

The New Zealand Ministry of Health Evidence Brief synthesised clinical outcomes from 25 studies and mental-health outcomes from 11 studies, all involving adolescents aged 12–18 years (53). Puberty suppression effectively paused puberty, reduced growth velocity as expected, and produced smaller-than-expected bone-density gains, with no new cardiometabolic abnormalities (53). Mental-health syntheses described improvements across treated cohorts, including reductions in suicidality, depression, anxiety, and dysphoria, although most studies had methodological constraints (53).

Across all reviews, mental-health trajectories for adolescents receiving puberty suppression were broadly stable or improved, and no review identified evidence of psychological harm attributable to treatment. Serious adverse events were rare. Bone-density reductions were the most consistent physiological effect and are well described in the primary literature. All reviews noted methodological constraints common in pediatric research with small populations and emphasised the need for larger, longer-term prospective studies with appropriate comparators and clearer accounting for co-interventions and structural determinants of health.

#### 4.2.3. Gender-affirming hormone therapy

**SAHCS GAHC Guideline (2021):** Gender-affirming hormone therapy is recognised as safe and effective, and is listed as essential medicines for tertiary care in South Africa in 2019. The goal of hormone therapy is to affirm the young person’s experienced gender, with treatment tailored to their desired outcomes, including for non-binary clients, through the administration of sex hormones.

##### Findings from original research

Twenty-nine primary studies focused primarily on GAHT in adolescents. Study designs were generally observational. Clinic-based prospective and retrospective cohort studies contributed the largest share of evidence (156,176–180). Additional record-review and registry- or insurance-based studies focused on treatment trajectories, continuation, and discontinuation (181–184). Several cross-sectional comparative studies assessed metabolic (176), neurocognitive (185) and psychological outcomes (121). Community- and online-survey studies provided broader youth data on mental health, wellbeing, and treatment experiences (97,186–188). Mixed-design and chart-enhanced studies contributed findings on psychosocial outcomes, adverse events, or patterns of GAHT use (179–189).

Most work came from specialist gender or paediatric endocrine services in high-income countries, supplemented by large online surveys of adolescents and young adults recruited through diverse community settings (97,186–188).

###### Physical and developmental outcomes

Clinic and registry studies consistently showed that testosterone and oestrogen regimens achieved expected physical and endocrine changes under routine monitoring, including development of secondary sex characteristics associated with the affirmed gender. Testosterone therapy was associated with increases in haemoglobin and haematocrit; small proportions of adolescents exceeded haematocrit thresholds and required dose adjustment or closer follow-up(177,189,190). Oestrogen therapy produced decreases in haemoglobin and haematocrit and modest increases in prolactin, typically without clinical sequelae (180,190).

Lipid profiles shifted in predictable ways by regimen and time on therapy, including higher triglycerides and changes in low- and high-density lipoprotein cholesterol, with no indications of acute organ toxicity reported in follow-up periods ranging up to two years(176,180,190). Testosterone-related increases in creatinine led to changes in estimated glomerular filtration rate (eGFR) when sex-specific equations were used, but values remained within clinically interpreted ranges (191). A cross-sectional lipoprotein analysis showed that transmasculine adolescents on testosterone had more “male-typical” and somewhat more atherogenic lipid particle profiles than cisgender female peers, broadly resembling cisgender males (166). Large registry and record-based cohorts similarly reported no high-frequency serious harms attributable to GAHT in adolescents under specialist care (182,183).

Pelvic pain emerged as a common issue among transmasculine adolescents, with high prevalence in both testosterone users and non-users; pain was somewhat less frequent in those on testosterone but often pre-dated GAHT and substantially affected school, work, and activities (192,193). A US retrospective cohort (N = 611) reported no venous or arterial thrombotic events during ~1.5 years of adolescent GAHT, despite high rates of baseline risk factors including obesity, smoking, migraine with aura, and family history of thrombosis (177). Mild headaches were somewhat more frequent among hormone users than non-users (189).

###### Mental health and psychosocial wellbeing

Across a range of study designs, access to GAHT was associated with neutral-to-favourable mental-health outcomes (194–196). Clinic-based comparative studies found that adolescents on GAHT, particularly testosterone, had lower anxiety and depression scores, trends toward lower suicidality, and less body dissatisfaction than untreated peers (195,196). In one cross-sectional study, adolescents on GAHT showed fewer parent-reported executive functioning difficulties than those not on GAHT, although the finding was modest and influenced by co-occurring ASD and anxiety (Strang et al., 2022). Neuroimaging work identified distinct amygdala–prefrontal connectivity patterns linked to differences in processing emotional cues, with no evidence of negative mental-health impact (185). A large prospective cohort across four US paediatric gender clinics reported sustained improvements in appearance congruence, depression, anxiety, positive affect, and life satisfaction over 24 months of GAHT, with earlier initiation associated with better baseline psychosocial functioning (194).

Large community and quasi-experimental studies found that access to gender-affirming hormones in adolescence was associated with lower odds of recent depression and reduced past-year suicide attempts, with the strongest protective effects observed when hormones were accessed in mid-adolescence (97,186,188). Among young adults, those who had received hormones during adolescence reported less severe psychological distress and lower suicidality than peers who desired but did not receive GAHT (188).

Prospective clinic cohorts reported improved appearance congruence and reductions in depressive and anxiety symptoms over 6–24 months of GAHT, with individual variation captured through measures such as the Transgender Congruence Scale, Beck Depression Inventory-II, and NIH Toolbox Emotion Battery (194). Other comparative studies showed domain-specific differences that generally favoured treated youth or indicated no detriment relative to comparison groups (97,185,196). Small neurocognitive studies suggested selective changes in areas such as processing speed, verbal memory, and aspects of emotion recognition with testosterone exposure – patterns consistent with typical adolescent neurodevelopment rather than treatment-emergent impairment – without evidence of global cognitive decline or worsening mood (185,197).

###### Safety and adverse events

Serious adverse events were uncommon. Service and record-based evaluations documented routine surveillance for erythrocytosis with testosterone and monitoring of liver enzymes, lipids, and glycaemic markers, with few clinically significant abnormalities observed (177,178,180,189,190,198,199). In one cohort study, testosterone initiation was associated with new or worsening acne in more than half of adolescents, especially when combined with progestin for menstrual suppression (200).

Comparative work on different routes of administration for testosterone found similar trough testosterone levels and no major differences in serious safety signals, although some lipid differences persisted over time (201). Community surveys did not systematically collect clinical adverse events but their mental-health findings support the safety of GAHT from a psychosocial perspective (97,186). Comparison studies of different estradiol products in transfeminine adolescents indicated that oral, transdermal, and subcutaneous preparations all achieved physiologic estradiol levels; transdermal estradiol, particularly when combined with spironolactone, produced effective testosterone suppression at relatively lower estradiol concentrations (178).

###### Treatment trajectories and patient experience

Clinical cohorts described GAHT initiation with gradual dose titration, regular follow-up, and achievement of secondary sex characteristics aligned with adolescents’ goals (177,179). Prospective assessments documented increases in appearance congruence and variable, generally favourable changes in mood and anxiety that reflected individual circumstances and concurrent psychosocial supports (194).

Cross-sectional studies reported that many adolescents perceived hormones as crucial for aligning their bodies with their gender and improving daily functioning, with experiences varying by age at initiation and duration of therapy (185,196,197).

Across cohort and registry-based studies that followed adolescents and young adults on GAHT, continuation rates were high, with clinic cohorts reporting over 90% ongoing use and large insured cohorts showing around 70% continuation at four years, and very low rates of permanent discontinuation or expressed regret (181–184). Large clinic cohorts reported that more than 90% of adolescents remained on GAHT at follow-up, with discontinuation typically reflecting external factors (such as cost, bullying, or difficulties with medication access), completion of desired physical changes, or a shift to a gender identity or embodiment that no longer required ongoing hormones rather than dissatisfaction with treatment (181,182). In a national insured cohort, adolescents who initiated GAHT before 18 years showed higher persistence than those starting in adulthood, challenging assumptions that minors discontinue more frequently (183). In a UK endocrinology service, only one adolescent discontinued GAHT, and no adolescents reported regret (184). Qualitative data from a Canadian–US online survey reinforced that adolescents and young adults who paused or discontinued hormones typically did so for contextual, developmental, health, or identity-related reasons rather than treatment harm, and most continued to identify as transgender or gender-diverse (187).

##### Findings from systematic reviews

Two systematic reviews synthesised evidence on GAHT initiated in adolescence: a systematic review commissioned for the Cass Review (175) and a SEGM-commissioned systematic review and meta-analysis (48). As noted earlier, the Cass Review-linked and SEGM-commissioned reviews were conducted in politicised contexts shaping methodological choices. For the GAHT evidence, this included strict adolescent-only criteria and extensive certainty downgrading, contributing to uniformly low or very low certainty ratings. Despite this, findings aligned with other reviews, showing expected endocrine changes and stable or improved mental-health outcomes.

The Cass Review-linked systematic review included 53 studies where GAHT began before age 18 (175). GAHT produced expected endocrine changes and body-composition shifts aligned with treatment goals. Bone-density z-scores increased after GAHT following earlier suppression, though many transfeminine adolescents remained below age-expected norms. Cardiometabolic findings were inconsistent but generally clinically mild (175). Psychological outcomes improved in several cohorts – particularly body satisfaction and general functioning – while others showed no measurable change. All evidence was rated low or very low certainty due to small samples, non-comparative designs, and overlapping cohorts (175).

The SEGM-commissioned review synthesised 24 studies of individuals under 26 years, including several cohorts with adolescent treatment initiation (48). Across very low-certainty evidence, meta-analyses suggested possible decreases in depression and gender dysphoria after GAHT and small improvements in global functioning. Bone-density findings were mixed, with small or no changes (48) Cardiovascular event rates were low, based largely on large mixed-age case series, and were the only outcomes rated moderate to high certainty. Evidence on suicidality, sexual function, and fertility was very limited. Overall evidence ratings were low due to confounding and co-interventions (48).

Across both reviews, findings indicate that GAHT reliably produces expected physical and hormonal changes, while mental-health and functional outcomes are generally stable or improved at group level. No review identified evidence of psychological harm attributable to GAHT. Adverse events were predominantly mild, and serious complications were rare. Evidence on bone, cardiometabolic, and psychosocial outcomes remains limited by methodological constraints common in adolescent research. Both reviews emphasised the need for longer-term prospective studies with appropriate comparison groups and clearer attribution of outcomes to GAHT versus preceding puberty suppression.

#### 4.2.4. Combined endocrine treatment pathways

While puberty-pausing medication and GAHT can be used independently (depending on age, Tanner stage, and therapeutic goals), they are often administered sequentially or with periods of overlap in clinical care. This section considers studies that follow adolescents across both phases of treatment to examine combined effects and trajectories.

##### Findings from original research

Fifty-two original research studies examined combined or sequential use of GnRHa followed by GAHT, or compared adolescents receiving endocrine care with those who did not. These included long-term Dutch registry cohorts (202–204) multi-site and prospective cohorts from the US and Canada (167,169,205–210), large US administrative datasets (211,212), and retrospective chart reviews from Canada, Israel, Turkey, the UK, Spain, Australia and the US (56,57,142,184,213–219). Additional cross-sectional comparative studies examined growth, bone health, metabolic indices, and body composition (220–225). A qualitative study examined psychosocial experiences related to body image, eating, and exercise across puberty suppression and GAHT (226). Complementary cross-sectional and registry-based analyses assessed venous thromboembolism risk, skeletal and body-composition development, sexual function, and longer-term employment outcomes in adolescents and young adults who had accessed puberty suppression and/or GAHT (198,208,220,227–229).

Survey and mixed-methods studies contributed data on satisfaction, continuation, and regret (181–183,230–233), while qualitative work described experiences of transitioning and detransitioning (123,124). One Dutch long-term follow-up study examined post-treatment sexual functioning in adulthood (234).

Across studies, follow-up ranged from short-term clinical monitoring to more than a decade in the longest Dutch cohorts, spanning puberty suppression, hormone initiation, and young adulthood. Adolescents typically began GnRHa in early-to-mid puberty and commenced testosterone or oestrogen in mid-to-late adolescence. Several cohorts included internal comparisons between adolescents with and without prior GnRHa exposure at the time of GAHT initiation (169,222,223).

###### Physical and developmental outcomes

Across clinical cohorts, adolescents who had begun puberty suppression and later initiated GAHT achieved expected growth targets under routine monitoring. In Dutch cohorts, trans girls reached an average adult height within ~1–2 cm of predictions after GnRHa and estradiol, while trans boys reached about 3 cm above predicted and mid-parental height, with earlier puberty pausing associated with greater gains (210,215). Multicentre US data similarly show that early-puberty GnRHa followed by testosterone is associated with a modest increase in final adult height of around 2–3 cm compared with parental height predictions and with peers starting testosterone only in later puberty (222). Additional US data showed that adjunctive oxandrolone, when initiated earlier in adolescence, was associated with modest additional height gains in transmasculine youth, although this remains a non-standard intervention and is one of several considerations in height-related counselling (217).

Across available studies, adolescents with prior puberty suppression entered GAHT with lower bone mineral density (BMD) or lean mass relative to peers without GnRHa exposure, reflecting expected physiological effects of delayed sex-steroid exposure. In a US cohort of 119 adolescents, AMAB youth had lower lumbar-spine BMD Z-scores at baseline (−0.61 vs 0.04), and low BMI and vitamin D deficiency further reduced BMD (221). Cross-sectional data from 56 adolescents found that those on GnRHa monotherapy – particularly AMAB youth – had the lowest total-body BMD Z-scores, while adolescents on testosterone or oestrogen were closer to average ranges; longer GnRHa duration correlated with lower BMD, and higher BMI was protective (223). In a prospective cohort of 19 AFAB adolescents initiating testosterone, those previously on GnRHa started with lower BMD and higher fat mass, but both GnRHa-exposed and non-exposed adolescents showed meaningful BMD gains over 12 months, with convergence between groups (169,223). A complementary Dutch study found that higher-dose estradiol after GnRHa was associated with greater lumbar-spine BMD recovery, while standard 2 mg regimens were insufficient to return Z-scores to baseline (235).

Long-term Dutch follow-up shows that BMD reductions during puberty suppression largely resolve with sustained GAHT (204). In a cohort of 75 adults who began GnRHa in adolescence and used GAHT for a median of ~12 years, BMD Z-scores returned to pretreatment ranges at the lumbar spine, hip, and femoral neck in those treated with testosterone, and at the hip and femoral neck in those treated with oestrogen; a modest persistent lumbar-spine deficit remained among transfeminine adults, and only a small number met clinical osteoporosis thresholds (204). A separate Dutch cohort of 322 adolescents showed that early-puberty initiation of GnRHa followed by GAHT was associated with hip bone geometry tracking reference curves for the experienced gender, while mid- or late-puberty initiation produced patterns aligned with sex assigned at birth, indicating a potential early-puberty window for skeletal development (202). DXA-based analyses suggest that early puberty suppression followed by GAHT can shift some pelvic dimensions – and, in AMAB youth, shoulder breadth – towards affirmed-gender ranges, whereas GAHT alone has little skeletal impact once puberty is complete (208). Breast-volume analyses likewise show similarly modest volumes after ~4–5 years of estradiol regardless of timing of puberty suppression, although most participants reported satisfaction (226).

Prospective US studies show favourable changes in body composition and functional capacity during combined endocrine pathways (167,167,207). Across 12 months of testosterone treatment, AFAB adolescents demonstrated reductions in body fat and increases in lean mass, with those previously on GnRHa tending to show larger gains and convergence in body composition across groups (167,207). A companion study of the same cohort found that adolescents with prior GnRHa entered testosterone therapy with higher baseline aerobic fitness and subsequently showed greater improvements in exercise performance over 12 months (167). Dutch body-composition data parallel these findings, showing that most lean-mass gain and fat-mass reduction in trans boys occurs within the first year of testosterone after puberty suppression, whereas in trans girls puberty suppression is associated with gradual lean-mass loss and fat-mass gain that estradiol only partially reverses (218).

Large US electronic-health-record data indicate that absolute prevalences of cardiometabolic diagnoses such as dyslipidaemia, dysglycaemia, liver dysfunction, and hypertension remain low among TGD adolescents, although overweight/obesity is common (212). Within this cohort, testosterone prescriptions were associated with higher odds of several metabolic diagnoses compared with TGD youth not on testosterone, underscoring the need for routine monitoring rather than indicating frequent severe toxicity (212).

Prospective cardiac-electrophysiology data from an Israeli cohort suggest minimal clinical impact of GnRHa or GAHT on QTc intervals within standard regimens. Transfeminine adolescents showed modest QTc increases after GnRHa that remained within normal limits, while transmasculine adolescents demonstrated small QTc decreases after testosterone, with no arrhythmias or clinically abnormal intervals observed (215).

Data from middle-income settings highlight the consequences of later presentation and constrained dosing. In a Turkish cohort of 30 adolescents receiving medical intervention, GnRHa and GAHT were generally effective, but median initiation age was ~16 years, AMAB adolescents often required higher or more frequent GnRHa dosing to achieve adequate suppression, and BMD – already lower at baseline in AMAB youth – declined further during GnRHa with little short-term recovery (236). A US comparison of leuprolide formulations found that both intramuscular Lupron and subcutaneous Eligard produced reliable clinical suppression, with slightly higher biochemical suppression rates for Eligard, particularly in adolescents starting at later Tanner stages (237).

Across studies, menstrual suppression in AFAB adolescents was common but not universal with puberty suppression and/or testosterone alone. In a US cohort of 232 youth on testosterone, one in four experienced breakthrough bleeding beyond the first year, whereas all adolescents maintained on concurrent GnRHa remained amenorrhoeic (217). Another US cohort similarly reported persistent bleeding across a range of testosterone doses, indicating that testosterone alone does not consistently suppress menses (56). In the Turkish cohort, menstrual suppression was generally achieved within a few months, though breakthrough bleeding occurred in a minority of cases, and suppression was more variable where insurance constraints limited optimal GnRHa dosing (236). (See section 4.2.5 for the full menstrual-suppression evidence synthesis.)

###### Mental health and psychosocial wellbeing

Across prospective and observational cohorts, adolescents moving from puberty suppression into GAHT demonstrated stable or improved mental-health trajectories. In a US cohort, starting puberty pausers or progressing to GAHT was associated with 60% lower odds of moderate-to-severe depression and 73% lower odds of suicidality over 12 months, compared with adolescents who had not yet accessed endocrine care (205). This pattern was also observed in a large US–Canadian longitudinal cohort of early socially transitioned adolescents, who showed stable and low levels of depression and anxiety across stages of medical transition – before puberty suppression, during suppression, and after GAHT initiation – with mental-health trajectories broadly comparable to cisgender peers (210). In a large US registry cohort, receipt of GAHT was associated with a 44% lower risk of emergency-department or inpatient suicidality diagnoses among TGD adolescents relative to TGD peers without GAHT (211). In a multi-site US cohort including adolescents with and without prior puberty suppression, GAHT initiation led to sustained improvements over 24 months in psychological wellbeing, social satisfaction, self-efficacy, and negative affect, with gains linked to improved appearance congruence (206). Complementing these cohort findings, qualitative evidence also suggests that puberty suppression or GAHT can ease body-image and eating-related distress for many adolescents, though a minority reported heightened appearance pressures after gender-affirming changes (226).

Population-level US military registry data found reduced mental-health diagnoses and psychiatric hospitalisations in the two years after puberty suppression or GAHT initiation compared with the two years prior (238). Danish national registry data following adolescents into early adulthood found that accessing masculinising GAHT was associated with higher odds of employment, suggesting possible long-term functional benefits of timely endocrine care despite persistent disparities relative to cisgender peers (198).

Access barriers and family context shaped outcomes. Youth in a US registry cohort who remained on waiting lists or experienced delays in accessing puberty pausers or GAHT showed transient worsening in depression and suicidality at 3–6 months before later stabilisation, whereas those who initiated endocrine care improved (205). In a prospective study of adolescents newly starting GAHT, one year of treatment was associated with reduced body dissatisfaction, depression, anxiety, and victimisation, and with improved psychosocial functioning, with greater benefits among adolescents with higher baseline family support and parental acceptance (209). Complementing these prospective findings, cross-sectional comparisons from the Netherlands showed better body image, appearance satisfaction and social functioning among adolescents who had progressed from puberty suppression into GAHT than among those earlier in their treatment trajectory (208). A smaller UK cohort assessed before treatment, after ~1 year of GnRHa, and after ~1 year of GAHT similarly found reductions in internalising symptoms after GnRHa and improvements in body dissatisfaction, gender dysphoria, and social motivation after GAHT (239).

###### Safety and adverse events

Safety findings across sequenced endocrine pathways were consistent with those reported for puberty suppression and GAHT individually, with no evidence of additional risk emerging specifically from the combined trajectory. Testosterone-related increases in haematocrit occurred infrequently and were effectively managed through routine dose adjustments (212,224). Liver enzymes remained normal, and clinically significant lipid or glycaemic abnormalities were uncommon in available cohorts of adolescents moving from GnRHa into GAHT, although routine metabolic monitoring remains important (159,202,214). A large US pediatric cohort found venous thromboembolism to be very rare (0.16%) and not associated with GAHT exposure, with all events occurring alongside secondary risk factors (227).

Cardiac and blood-pressure monitoring findings were reassuring. No clinically significant QTc prolongation was observed during puberty suppression or subsequent GAHT, including among adolescents taking psychotropic medications known to affect QT intervals (214,215). A transient rise in diastolic blood pressure during GnRHa treatment remained within normal ranges and returned to baseline after testosterone initiation (213).

As described in the physical and developmental outcomes section, short-term reductions in bone mineral density are an expected effect of puberty suppression. In the context of safety monitoring, these findings underscore the importance of avoiding prolonged monotherapy when GAHT is indicated and ensuring follow-up DXA where clinically appropriate. Across prospective cohorts, BMD increased after GAHT initiation and long-term studies report substantial recovery with sustained hormone therapy (169,204,223).

No service-level reviews or large electronic-health-record analyses identified new, high-frequency adverse events attributable to the combined pathway (211,212). Common side effects – such as injection-site discomfort, acne, and mild mood fluctuations – were typical of adolescent testosterone therapy and were generally self-limited or manageable through routine clinical adjustments (224).

###### Treatment trajectories and patient experience

Longitudinal Dutch registry cohorts found high continuation of GAHT into adulthood among adolescents who began care with puberty suppression, with 98% maintaining GAHT at follow-up and most discontinuations reflecting prior gonadectomy rather than stopping gender-affirming care (193,194). Adolescents commonly reported relief of dysphoria during puberty suppression and satisfaction with bodily changes after starting GAHT, alongside improvements in daily functioning and alignment with gender goals (216,219,230).

Multiple adolescent cohorts show similarly high continuation and very low regret, with permanent discontinuation due to reidentifying with their birth-registered sex consistently uncommon (181,182,203,204) (142,183). In a large US cohort, reidentification occurred in only ~0.5% of adolescents after starting medical care (181). A second long-term US cohort likewise reported 0.5% permanent discontinuation and no adolescent regret, with continuation rates equal to or higher than those of adults (182). Most discontinuations reflected having achieved desired changes or difficulties accessing medication rather than dissatisfaction with treatment (182). UK clinic data showed comparably low discontinuation of puberty pausers or GAHT, generally linked to service-level barriers or psychosocial challenges rather than adverse effects (184). In a large US military-health-system cohort, adolescents similarly demonstrated high four-year continuation, exceeding adult continuation rates (183). A prospective Australian cohort reported that 90.9% of adolescents diagnosed with gender dysphoria continued some form of gender-affirming care, although outcome definitions combined gender identity status and treatment status and most outcomes were measured after age 18 (232).

One mixed-methods study found that shifts in gender-related medical requests often occurred before treatment initiation – particularly among nonbinary youth – while shifts after starting treatment were uncommon and rarely reflected dissatisfaction with GAHT (218). Survey data from a large US pediatric gender clinic further showed that among adolescents and young adults who had ever taken hormones, temporary discontinuation was relatively common (21%) but overwhelmingly driven by external barriers – such as injection burden, access difficulties, or medical insurance challenges – rather than dissatisfaction or regret; many participants who had not accessed puberty suppression expressed wishing they could have (231).

Qualitative evidence underscores the need for responsive, non-pathologising support. Two studies including older adolescents described limited psychosocial support during transitions and detransition, occasional gatekeeping or invalidation, and challenges stopping hormones safely (123,124). A minority described supportive clinical care, while many experienced indifference – particularly when detransition did not involve regret, or when they continued to identify as trans or nonbinary after discontinuing GAHT or other aspects of medical transition (123,124).

Delays or interruptions generally reflected structural barriers – such as injection logistics, appointment availability, and insurance or service-coordination challenges – rather than identity-related factors (182,184,231). Evidence on identity change after treatment initiation remains limited but indicates that reidentification is rare: in a state-wide Australian audit, only 1% of adolescents who initiated puberty pausers or GAHT later reidentified with their birth-registered sex, most before substantial physiological change (142).

In a long-term Dutch follow-up study of adolescents who received sequential puberty suppression and GAHT, timing of suppression (early vs late) did not adversely affect adult sexual functioning; sexual satisfaction was more strongly influenced by body confidence, access to surgery, and partner-related stigma (234). Qualitative interviews with US young adults who had received puberty pausers and/or GAHT similarly described predominantly positive or neutral changes in sexual desire and function, with no adverse patterns attributed to prior GnRHa use (229). In follow-up extending into late adolescence and early adulthood, adolescents reported high satisfaction with the gender-affirming medical care they received (puberty suppression and/or GAHT), with regret consistently rare (240).

##### Findings from systematic reviews

Eleven academic systematic reviews (22,24,25,29–31,35,37,40,41,46) and three grey-literature reports (50–52) synthesised outcomes for adolescents receiving combined multiple endocrine interventions – typically puberty suppression followed by GAHT – or analysed outcomes across both interventions. Despite differences in scope, these reviews consistently described expected endocrine effects, high continuation, and generally stable or improved mental-health and functional outcomes.

Among the grey-literature reviews, the Utah report (52) was mandated by state legislation restricting adolescent GAHT. This context shaped several design choices – such as a narrow focus on safety, stricter evidence-grading criteria, and conservative weighting of observational studies – however, its substantive findings aligned with broader academic reviews.

Across academic reviews, GnRHa reliably halted pubertal progression and development of dysphoria-related secondary sex characteristics, while subsequent GAHT induced pubertal changes aligned with affirmed gender (25,35,40,41,46). Treatment patterns across adolescent cohorts showed puberty suppression typically beginning in mid-adolescence with GAHT introduced later; progression from GnRHa to GAHT was the norm, with very few adolescents discontinuing treatment (31,35,40,41). A dedicated continuation review reported continuation rates above 90% among adolescents starting GAHT before age 18, with discontinuation usually linked to external or structural factors – such as insurance or access barriers, family or social pressures, bullying, unrelated medical concerns, or evolving identity – rather than treatment-related effects; regret was rare (31). Broader adolescent treatment reviews similarly reported discontinuation rates generally under 10% (40).

Alongside these continuation-focused syntheses, one additional systematic review examined the prevalence of medical discontinuation and detransition across endocrine pathways (29). In this review, “detransition” referred specifically to stopping or reversing medical treatment – such as discontinuing GnRHa or GAHT – and did not include broader identity changes or psychosocial experiences. The review included 15 observational studies (3,804 adolescents and 3,270 adults) and analysed adolescent outcomes separately. Adolescent point-prevalence estimates for discontinuation or detransition were consistently in the low single digits: shifts in request before any treatment were reported as low as ~0.8% in some cohorts; GnRHa discontinuation ranged up to ~7.6%; and GAHT discontinuation generally ranged from ~2–3% when adolescent-only data were available (29). Reasons for discontinuation among adolescents included resolution or desistance of gender dysphoria, poor compliance, and other psychosocial contributors—not always well specified in the primary studies – but generally relating to family, school, or mental-health stressors. Evidence quality was rated low, constrained by retrospective designs, small event counts, heterogeneous definitions, and variable disaggregation by age (29).

Mental-health and psychosocial outcomes were consistently stable or improved across combined pathways (22,24,25,30,35,37,40,41,46). One systematic review reported reductions in depression and anxiety and improvements in quality of life after GAHT – often following earlier puberty suppression – with no evidence of deterioration; the authors rated the strength of evidence as low (22). Reviews of puberty suppression described improvements in psychosocial functioning, mood, and peer relationships, with further gains in body image and wellbeing after GAHT (25,35,41). An adolescent treatment review reported decreases in depressive symptoms and improvements in global functioning, albeit across heterogeneous observational cohorts (40). A broader mental-health review emphasised high baseline distress among transgender adolescents and found that gender-affirming medical care was associated with more favourable mental-health trajectories, particularly in affirming family and school contexts (37).

Suicidality-focused evidence showed the same overall pattern of stable or improved outcomes and no indication of harm (22,24,30,35,37). Adolescents who accessed puberty suppression and GAHT demonstrated lower suicidality than untreated peers, with protective effects from affirming family environments, inclusive schools, and accessible legal recognition processes (24). No review identified increased suicidality attributable to endocrine interventions (22,24,35). Qualitative syntheses described puberty pausers and GAHT as protective, with adolescents reporting improved self-esteem, identity congruence, and outlook after accessing treatment, and identifying delays, denials, and hostile clinical encounters as major sources of distress (30).

Bone-health findings across reviews were consistent: BMD z-scores typically declined or increased more slowly during GnRHa treatment and rose after GAHT, with partial normalisation over time (25,35,40,41,46). Transfeminine adolescents often remained below age-expected norms even after years of GAHT, prompting recommendations for ongoing DXA monitoring, adequate oestrogen dosing, vitamin D assessment, and caution regarding prolonged suppression without progression to GAHT (25,41,46). Growth-velocity declines during suppression – particularly when initiated later in puberty – were followed by catch-up growth after GAHT, with final height typically within target ranges and no evidence of pathological growth restriction (40,41).

Cardiometabolic and broader safety outcomes were consistently reassuring across adolescent cohorts. Reviews described expected physiological shifts in lipids, haemoglobin, and body composition with puberty suppression and GAHT, with most cardiometabolic parameters remaining within normal reference ranges(25,40,41,46). Serious adverse events were rare. Common side effects – including headaches, hot flushes, acne, mood changes, weight gain, and implant-site discomfort – were generally mild, anticipated from treatment mechanisms, and seldom led to discontinuation (25,30,35,41). A small number of isolated stress fractures were reported during puberty suppression, usually in adolescents with additional risk factors such as low baseline bone density or high-impact physical activity; reviews noted these as rare events not indicative of an elevated fracture risk at population level (25,35,41).

Across reviews, certainty was consistently rated low or very low due to observational study designs, small and overlapping samples, short follow-up periods, limited adjustment for confounding, and poor representation of marginalised adolescents (22,24,30,35,37,40,41,46). Reviews identified no evidence that puberty suppression or GAHT worsened mental-health outcomes; harms were more often associated with delayed or restricted care, discrimination, and a lack of familial or school support (30,37). Despite these limitations, no review identified psychological harm attributable to endocrine interventions when delivered within gender-affirming care. Instead, evidence consistently showed expected physiological effects, high continuation and low regret, and stable or improved mental health and wellbeing, alongside well-documented risks associated with delayed or denied care.

Three grey-literature reviews – the NSW Evidence Check, the RAND report, and the Utah review – synthesised evidence on puberty suppression and GAHT within combined (50–52). Despite different mandates, all drew on overlapping primary studies and reached convergent conclusions on benefits, safety, and limitations of the evidence base.

The NSW Evidence Check reported that puberty pausers effectively halted pubertal progression and reduced emotional and behavioural problems, while GAHT produced expected physical changes and was associated with reduced depression, anxiety, and suicidality in several cohorts, with neutral findings in others (50). Overall, affirming medical care was linked to improved mental-health and quality-of-life outcomes, although certainty was constrained by small, uncontrolled studies. Bone-density declines during suppression were generally followed by improvements after GAHT, and growth and cardiometabolic measures remained within reference ranges. Regret was rare, with only isolated reports across adolescent endocrine studies, and treatment was generally well tolerated (50).

The RAND report analysed puberty-suppressing hormones and GAHT as sequential but distinct interventions. For puberty suppression, low-certainty evidence showed effective pausing of targeted pubertal changes, reductions in gender dysphoria, and mental-health outcomes ranging from improvement to stability, with no harms reported (51). BMD reductions during suppression were common, followed by increases after GAHT. For GAHT, very low-certainty evidence indicated improvements in depression, anxiety, functioning, and gender dysphoria, with dysphoria-related measures showing the largest effects. Bone density increased following GAHT; cardiometabolic parameters generally remained within healthy ranges; side effects were mild; continuation was high; and regret was rare (51).

The Utah review concluded that GAHT – typically following suppression – improved mental health, functioning, body satisfaction, and induced expected physical changes (*Utah Report*, n.d.). Treated adolescents showed lower depression, anxiety, and self-harm indices compared with untreated peers, with sustained improvements in dysphoria and quality of life over time. BMD typically declined during GnRHa treatment but stabilised or partially recovered once GAHT was introduced, with no clinically significant growth or metabolic concerns. Longer-term follow-up of individuals who initiated treatment before 18 years demonstrated ongoing psychological benefits and no signal of increased venous thrombosis risk; cancer risks were comparable to cisgender reference populations. The authors concluded that restricting access to GAHT for adolescents was not justified by available evidence.

All three grey-literature reviews noted that evidence remains limited by observational designs, confounding, short follow-up, and reliance on high-income clinic samples (50–52). Despite these limitations, they consistently found that combined endocrine pathways produced expected physiological changes, high continuation and low regret, and stable or improved mental-health and wellbeing, with no evidence of psychological harm attributable to treatment and clear risks associated with delayed or restricted access.

#### 4.2.5. Menstrual suppression as gender-affirming care

**SAHCS GAHC Guideline (2021):** Menstrual bleeding can contribute to dysphoria and might persist despite the use of testosterone (or in the absence of testosterone treatment for those clients who do not wish to use it). The guideline notes that in such cases agents such as leuprolide, medroxyprogesterone acetate and anastrazole may be considered.

The guideline does not include content on menstrual suppression as a standalone gender-affirming intervention for TGD adolescents, nor does it include adolescent-specific recommendations. Guidance applies broadly across age groups.

##### Findings from original research

Five original research studies examined menstrual suppression among TGD adolescents (241–245). Across retrospective cohort reviews and one large cross-sectional baseline study, methods included norethindrone acetate, levonorgestrel intrauterine devices (IUDs), etonogestrel implants, depot medroxyprogesterone, combined oral contraceptives, danazol, and testosterone with or without adjunctive progestins. Sample sizes ranged from 101 to 530 participants, with most adolescents aged 12–18 and recruited through specialist gender services. No systematic reviews were identified. Outcome measures were typically chart-based or clinician-documented, with few validated symptom or wellbeing scales, which limits causal inference. Nonetheless, findings were consistent across settings.

###### Physical and developmental outcomes

Across methods, amenorrhoea rates were high. In a cohort of 101 adolescents using menstrual-management methods, 80% achieved amenorrhoea within 1–6 months, rising to 93% by 9–18 months (245). Danazol produced amenorrhoea in 85% of transgender and non-binary adolescents, with median onset at three months (244). A Long-Acting Reversible Contraception (LARC) focused cohort similarly reported high continuation and acceptable bleeding profiles, with nearly half of LARC users experiencing expected bleeding changes (241). In a separate clinic cohort, 94% of adolescents using menstrual suppression (across a range of methods) reported it to be effective (243).

A large US cohort (N = 220) reported median time to amenorrhoea ranging from 77–182 days depending on method, with norethindrone acetate and depot leuprolide producing the most rapid cessation (77–78 days), testosterone alone taking a median of 151 days, and fewer than half of all adolescents achieving amenorrhoea within six months (242).

Pain-related outcomes also improved. Danazol was associated with substantial improvements in dysmenorrhoea and endometriosis-related pain (244), and a majority of adolescents using progestins or LARC reported reductions in pelvic pain or menstrual-related discomfort (245).

###### Mental health and psychosocial wellbeing

Improved mood, reduced menstrual-related dysphoria, and greater day-to-day functioning were commonly documented, though usually through narrative clinical notes rather than validated scales. In chart-based follow-up, most adolescents experienced reductions in dysphoria and mood symptoms after achieving amenorrhoea (245). Danazol users similarly reported improvements or complete resolution of menstrual-related dysphoria (79%) (244).

Cross-sectional baseline data from a clinic cohort (N = 530) found high rates of menstrual-related distress (93%) and strong unmet need among non-users (88% expressed interest in suppression) (243). However, suppression use at baseline was not associated with differences in broader mental-health indicators (gender dysphoria, depression, anxiety), likely reflecting the limitations of a single time-point assessment rather than lack of treatment benefit.

###### Safety and adverse events

Across studies, adverse events were generally mild and infrequent. Progestin-based methods and LARC showed low rates of side effects, with infections occurring in fewer than 1% of IUD users and only rare instances of expulsion or pelvic pain (241). Norethindrone acetate was associated with minor side effects in around one-fifth of adolescents, most commonly mood changes or irregular bleeding (245). Danazol produced mild androgenic effects – such as acne or voice change – in roughly one-third of users, though these were seldom reasons for discontinuation and were sometimes welcomed as gender-affirming changes (244).

No study identified serious adverse events, perforations, or clinically significant complications. Across the evidence base, authors emphasised the importance of counselling young people on expected bleeding changes and side-effect profiles as part of routine menstrual-suppression care.

###### Treatment trajectories and patient experience

Continuation rates were high across menstrual-suppression methods. One multisite review reported 92% continuation at 12 months and ~75–80% at 24 months for IUDs and implants (241). Norethindrone acetate and levonorgestrel IUDs had continuation rates between 76–84% at 9–18 months (245). For danazol, 58% of TGD adolescents remained on treatment beyond one year, with discontinuation most commonly linked to initiating testosterone rather than intolerance or lack of efficacy (244).

Qualitative and chart narratives emphasised significant relief from menstrual-related dysphoria, increased comfort in school and social environments, and improved alignment with affirmed gender (243–245). Across studies, achieving amenorrhoea was associated with satisfaction and continuation.

#### 4.2.6. Fertility counselling and preservation

**SAHCS GAHC Guideline (2021):** Fertility counselling should occur before initiating both puberty pausing and GAHT, as part of a developmentally appropriate informed-consent process delivered by clinicians with relevant training. The guideline notes that pausing puberty at its onset may prevent the possibility of harvesting sperm or ova later, an effect that is irreversible unless GnRHa is stopped in time. Because hormone therapy can also affect future fertility, counselling should also include information for young people and their families on fertility-preservation options before starting GAHT.

##### Findings from original research

Fifteen original research studies examined fertility counselling, fertility preservation practices, and reproductive decision-making among TGD adolescents, as well as TGD adults who initiated puberty pausers and/or GAHT in adolescence (246–260). Most were retrospective clinical reviews from specialist gender services or fertility centres in high-income settings (Australia, Europe, Israel, the United Kingdom, and the United States), supplemented by a small number of qualitative case series and long-term follow-up studies, with sample sizes ranging from small cohorts to large registries.

Across studies involving transfeminine adolescents, fertility preservation was technically feasible through semen collection, testicular biopsy, testicular sperm extraction (TESE), or testicular tissue cryopreservation (246–248,256,257,259). Sperm retrieval success ranged from around 68% in testicular biopsy cohorts to 100% in small TESE case series before GAHT (248,257), and 82–83% achieved storage in a large UK cohort (259). However, semen quality was frequently below WHO reference values, with high rates of impaired morphology, concentration, and motility – even among hormone-na ve adolescents (246,247,254,259). A comparative study of transfeminine adolescents and cisgender adolescent cancer patients undergoing fertility preservation found broadly similar semen parameters between groups, although azoospermia was more common among transfeminine adolescents (over one-fifth of the cohort) (254). Testicular tissue analyses showed retained immature germ cells in most adolescents and occasional mature sperm when treatment began at later pubertal stages (253,256,257).

Among transmasculine adolescents, oocyte cryopreservation was successful and generally safe, with high oocyte yields and only mild complications (249,251). Outcomes were similar for those with and without prior testosterone exposure (249,251). Studies emphasised the importance of dysphoria-sensitive protocols, including transabdominal ultrasound monitoring, gender-affirming communication, and minimising delays to testosterone initiation (249, 251).

Despite feasibility, uptake of fertility preservation was consistently low. US clinic reviews reported that <10% of adolescents proceeded to cryopreservation even when counselling was documented (250,255). A pre–post quality-improvement intervention doubled counselling rates but did not increase preservation (258). In Denmark’s public system, fewer than half of transfeminine adolescents chose cryopreservation despite universal offer (247). Long-term follow-up in the Netherlands showed that limited counselling and structural barriers during adolescence contributed to later distress about infertility, with many adults stating they would now opt for preservation (252).

Qualitative studies illuminated the decisional context: adolescents described balancing urgent relief from dysphoria and fear of pubertal progression against the possibility of future genetic parenthood (249,250,252,260). Masturbation-based semen collection was commonly reported as unacceptable or highly dysphoria-provoking, and surgical options, though invasive, were frequently preferred when accompanied by consistent parental, psychological, and clinical support (248,260).

Taken together, the primary studies show that fertility preservation procedures are technically feasible and generally safe for TGD adolescents in small observational cohorts, with multiple options for both transfeminine and transmasculine youth (248,249,256,257). However, semen quality is often reduced (246,247,254,259), uptake remains low even when counselling is routine (247,250,255,258), and decisions are heavily shaped by age, dysphoria, urgency to initiate GAHT, procedural invasiveness, and structural barriers such as cost, service availability, and medical insurance coverage (248,249,252,260). Long-term follow-up findings underscore that reproductive desires evolve over time, reinforcing the need for structured, iterative, developmentally appropriate fertility counselling grounded in adolescents’ current priorities while acknowledging future uncertainty (252).

##### Findings from systematic reviews

One academic systematic review (38) and two grey literature reports (50,51) examined fertility considerations among TGD young people.

The academic systematic review included 76 studies examining fertility desires, counselling, and fertility preservation among TGD people, with adolescent findings summarised separately (38). Adolescent data, drawn mostly from European cohorts, showed low fertility preservation uptake (<15%) despite higher rates of counselling, with oocyte or sperm cryopreservation performed only in small proportions before GAHT (38). Puberty pausing with GnRHa was the most common initial stage of gender-affirming care for adolescents, with fertility preservation discussions occurring before or at its initiation. Fertility preservation options such as semen or oocyte cryopreservation were available in small proportions, whereas tissue-based methods (ovarian/testicular tissue cryopreservation and testicular biopsy in early puberty) were described as technically feasible but experimental due to their uncertain future reproductive utility (38).

Reported barriers included anxiety about procedures, discomfort with genital-focused examinations, cost, and lack of parental agreement, while adolescents expressed some desire for future parenthood but less frequently than adults (38). No serious adverse events or post-fertility preservation regret were reported. Overall, the review found that fertility preservation is rarely pursued in adolescents, despite counselling, due to psychological, ethical, familial, and financial constraints and the limited availability of adolescent-specific data (38).

The RAND Cooperation grey literature report synthesised nine studies on fertility preservation noting that under-18 data was often embedded in mixed-age samples (51). Across small cross-sectional studies and case reports, oocyte, embryo, and semen cryopreservation were generally successful, most commonly when undertaken before puberty suppression or GAHT, or after pausing GAHT for at least three months; one case report documented successful oocyte preservation without pausing testosterone, and a small number of transfeminine adolescents showed azoospermia before hormone treatment for unclear reasons (51). Adverse events were limited to mild or moderate ovarian hyperstimulation and minor procedural effects, with no major complications reported, and long-term outcomes such as pregnancy or live birth were rarely documented. The review rated the fertility-preservation evidence as very low certainty because of small samples, high risk of bias, imprecision, and indirectness. It reported that viable gametes were successfully retrieved in small cohorts after pausing GAHT for several months, although these findings should be interpreted cautiously given the limited evidence base (51).

The Rapid Evidence Check for the New South Wales Ministry of Health reached similar conclusions (50). It found that semen and oocyte cryopreservation are effective, low-risk, but underused, with uptake constrained by cost, late referral, low awareness, and procedural distress – particularly when genital-focused examinations or oocyte retrieval exacerbate dysphoria (50). The review noted potential impacts of puberty pausers and GAHT on gamete development, although direct adolescent evidence was limited. Reported findings included reduced semen quality relative to cis male reference values, highly successful oocyte cryopreservation after pausing testosterone (with outcomes comparable to cis women), successful TESE in most adolescent trans girls with adequate testicular volume, and low adverse-event rates (mostly mild ovarian hyperstimulation) (50). Despite low evidence certainty, the review reinforced that structured fertility counselling is essential, including in settings where access to preservation technologies is limited (50).

#### 4.2.6. Synthesis of endocrine evidence and guideline implications

The 2021–2025 endocrine literature for TGD adolescents shows a consistent pattern: endocrine interventions generally achieve their intended physiological effects; adverse events are typically mild, reversible, and manageable under specialist care; and group-level mental-health outcomes are broadly stable or improved. Although methodologically constrained and concentrated in high-income clinical settings, the evidence provides a coherent short- to medium-term picture of effectiveness and safety.

Across puberty-pausing medication, GAHT, and combined treatment pathways, GnRHa reliably halts unwanted pubertal development and GAHT induces expected masculinising or feminising changes, with endocrine and metabolic parameters usually remaining within clinically acceptable ranges under supervised care. Bone density reductions during suppression are common but appear largely reversible with subsequent hormone-induced puberty, and serious adverse events are rare. Mental-health outcomes are heterogeneous at the individual level yet broadly neutral-to-favourable across cohorts: adolescents who access puberty suppression and/or GAHT generally show reductions in depressive symptoms and suicidality, alongside improved appearance congruence and functioning, compared with their own baseline and with peers who want but cannot access treatment. Longitudinal data indicate high continuation into adulthood and very low regret among adolescents who commence endocrine care, and long-term follow-up suggests that puberty suppression does not compromise adult sexual wellbeing.

Although menstrual suppression is not addressed in the 2021 SAHCS Guideline, evidence from this rapid review shows it is a safe, effective, and highly valued component of care for TGD adolescents who menstruate. Across methods, studies report high rates of amenorrhoea, improvements in pain, reductions in menstrual-related dysphoria, and high satisfaction, with few significant adverse effects. While the evidence is methodologically limited, it supports including menstrual suppression as a recommended option, accompanied by counselling on side effects, consent, and how suppression may interact with future GAHT or reproductive-health planning, including the possibility of preserving and exercising reproductive intentions in adulthood.

Fertility preservation evidence remains limited but consistent. Studies show that semen and oocyte cryopreservation are feasible and generally safe for adolescents, though uptake is low and often constrained by dysphoria related to procedures, cost, and timing of referral, underscoring the need for early fertility counselling.

Key evidence gaps cut across all endocrine interventions. Long-term skeletal and cardiometabolic trajectories into adulthood remain insufficiently characterised, as do very rare adverse events. Most studies are drawn from high-income, tertiary-centre settings, with limited data on non-binary adolescents and minimal disaggregation by structural determinants of health. There is almost no primary evidence from the Global South, where resource constraints, stock-outs, and workforce shortages shape what is feasible in practice.

Within these limitations, the literature supports the current SAHCS Guideline framing of puberty suppression and GAHT as clinically effective and generally safe components of adolescent gender-affirming care when delivered within multidisciplinary services and with appropriate monitoring.

Tables 4 and 5 summarise key implications for guideline development for puberty suppression and GAHT, respectively, and highlight areas where recommendations may be strengthened or refined. Evidence from combined treatment pathways is incorporated across both tables rather than presented separately. Implications for fertility counselling and preservation are similarly integrated to reflect their relevance wherever endocrine interventions are considered. The section concludes with Table 6 outlining implications for future guideline development related to menstrual suppression.

### 4.3. Surgical care

Surgical care for TGD adolescents is outlined in the SAHCS GAHC Guideline (2021) in Chapter 2 (Informed Consent) and Chapter 7 (Surgery).

**SAHCS GAHC Guideline (2021): Gender-affirming surgery (GAS)** refers to a set of reconstructive surgical procedures intended to help TGD people live comfortably in their affirmed gender, alleviating gender dysphoria and supporting social integration. These procedures may include masculinising or feminising chest surgery (top surgery), genital reconstruction (bottom surgery), as well as facial, laryngeal and other procedures. GAS should not be viewed as cosmetic but rather as medically necessary. There is no standard path. Each client’s surgical needs, if any, are individualised, with the aim of enabling autonomy, dignity, and improved well-being. Satisfaction rates are generally high, but irreversible changes and the diversity of surgical options mean comprehensive, client-centred preparation is essential.

**For adolescents**, gender-affirming surgery is most commonly limited to masculinising chest reconstruction for those assigned female at birth who are mature enough to require and benefit from surgery. Other surgical options are seldom considered before adulthood. Access to any surgical intervention is grounded in South African law and requires the adolescent to be over 12 years old, assessed as mature and cognitively able to consent, and supported by their parent or guardian. Informed consent is a thorough, collaborative process; pre-operative psychological assessment and documentation by an experienced provider is standard. The recommended practice is a multidisciplinary, team-based approach incorporating mental health and surgical professionals, with robust, ongoing psychosocial support before and after surgery.

Access to surgery is heavily constrained by limited resources, lengthy public sector waiting lists, and high costs in private care. These factors collectively create profound issues of access and equity.

#### 4.3.1. Overview of the evidence base

We identified ten original research studies reporting outcomes of gender-affirming surgery in adolescents (262–271) and 15 studies in which surgery was examined within broader gender-affirming care trajectories that also included puberty pausers and/or GAHT (149–162) (231). Two grey-literature systematic reviews assessed surgical outcomes in youth (50,51), and one academic review included a small adolescent subgroup within a broader synthesis of pediatric gender-affirming healthcare but did not report surgery-specific outcomes (40).

Across the evidence base, study designs were predominantly observational. Most papers used retrospective analyses of clinical records or cohort data, including single-center case series and chart-based cohorts (204,233,234,262,268–270,272,273). Large retrospective registry-based cohort studies drew on national surgical quality or administrative datasets (264–266). A smaller number of studies used prospective or longitudinal cohort designs with planned follow-up assessments (263,271,274–277). Recruitment was almost entirely from specialist gender clinics, hospital-based programs, or registry-linked clinical cohorts in high-income settings, including the USA (139–144,146–148,150,156,158–160), the Netherlands (204,234,272,274), Germany (275,277), Norway (273,278), Israel (268), and a binational North American cohort spanning the USA and Canada (187). Sample sizes ranged from single-surgeon cohorts and practice series (N=53–208; (268,270,271) to large national registry–based cohorts (N≈108–2,504; (264–266).

Outcome assessment combined objective clinical and registry data with patient-reported measures across studies. Routine perioperative documentation and standardized complication coding from hospital records or national surgical registries captured short-term surgical safety outcomes such as hematoma, wound complications, reoperation, readmission, and mortality (264–266). Alongside these clinician-coded outcomes, many studies used validated self-report instruments to assess chest dysphoria, body image, gender congruence, satisfaction, and broader psychosocial functioning, including the Chest Dysphoria Measure, Transgender Congruence Scale, Body Image Scale, and depression and anxiety scales (263,270,271,275,277). Across the evidence base, outcome measurement typically combined clinician- or registry-coded surgical complications with validated self-report scales assessing dysphoria, appearance congruence, body image, mental health symptoms, satisfaction with care, and social or relational functioning (231,233,279).

Follow-up for surgical complications was usually confined to the immediate perioperative period or 30-day window in hospital- and registry-based cohorts, using EMR, anesthesia records, or standardized registry fields to identify adverse events, reoperations, and readmissions (262–266). In contrast, several clinic-based series reported complications and revision needs over longer intervals, drawing on surgeon-led chart review and follow-up visits, with some explicitly requiring ≥3–12 months of follow-up for inclusion (268–270). Revision procedures were captured only in clinic-based surgical cohorts, primarily via surgeon or team review of operative and follow-up records in single-surgeon or single-centre studies (263,268,269,271) and through combined chart abstraction and patient-reported follow-up in the multi-year survey cohort (270). By contrast, registry-based analyses relying on ACS NSQIP data do not report longer-term revision surgery beyond the 30-day postoperative window (264–266). Outcomes assessed with more than 12 months of follow-up included chest dysphoria, body image, appearance congruence, satisfaction, and mental health in primary surgical cohorts, often using validated scales such as the Chest Dysphoria Measure, Transgender Congruence Scale, and symptom inventories (267–271). Longer-term mental health, psychosocial functioning, quality of life, treatment trajectories, and gender dysphoria were evaluated over multiple years in broader longitudinal gender-affirming care cohorts that followed adolescents from puberty suppression through hormones and, for many, surgery (233,234,272–278,280).

The evidence base focuses almost entirely on masculinizing chest reconstruction in adolescents and young adults, with only a very small number of other procedures reported for minors (handful of hysterectomies, one vaginectomy, and one facial feminization case in a pediatric registry cohort) (262,265). Studies that examined surgery within broader treatment pathways typically followed adolescents from puberty suppression into gender-affirming hormone therapy and, for many, subsequent chest or genital surgery, with multidisciplinary teams contributing longitudinal clinical, endocrine, and psychosocial data across these stages of care (234,274,280).

Key strengths across the evidence base included multiple prospective or longitudinal psychosocial cohorts with baseline and follow-up assessments that evaluated changes in mental health, functioning, body image, chest dysphoria, and gender dysphoria over time (233,234,263,267,274–278). Another strength was the inclusion of large multi-site clinical cohorts and national or binational datasets, which increased statistical power, enabled subgroup analyses, and enhanced external validity (234,264–266). Another important strength was the consistency of findings across diverse methods and settings, with qualitative interviews, clinic-based cohorts, and registry or survey data all indicating substantial improvements in dysphoria, functioning, and satisfaction after gender-affirming interventions, alongside very low rates of serious surgical complications and regret (263–265,267–271).

Methodological limitations largely mirror those seen in other adolescent gender-affirming care domains, including small or overlapping samples (with some Dutch longitudinal programmes contributing the same participants to multiple reports), absence of randomized designs, heterogeneous outcome measures, short follow-up in many cohorts, and geographically narrow evidence, with no studies from low- or middle-income countries and limited reporting on non-binary or neurodivergent youth. Because participants can enter at different stages of care, contribute to multiple sub-analyses, and appear across several linked publications or in both clinical and national registry studies, it is not possible to derive a single, non-duplicated total N of adolescents who have received surgery in this literature. Grey-literature and academic systematic reviews synthesising these studies highlight these design constraints, and generally rate the evidence for psychosocial 2benefits of gender-affirming surgery in youth as low to very low certainty—primarily due to small sample sizes, uncontrolled designs, and imprecision, rather than conflicting outcome patterns (50,51).

To aid interpretation, we group surgical findings into two categories: studies examining surgical procedures alone, and studies assessing surgery in combination with other forms of gender-affirming healthcare.

#### 4.3.2 Gender-affirming surgery

Across the ten surgery-only studies, evidence centres almost entirely on masculinising chest reconstruction among adolescents and young adults, reflecting the dominant pattern of surgical access in this age group (262–271). (The only exception came from a large national surgical registry in the USA, which listed a very small number of other surgical procedures among patients under 18, including hysterectomy (n = 4), vaginectomy (n = 1), and facial feminisation surgery (n = 1) (265). No study required prior GAHT for eligibility, although one explicitly required at least one year of mental health therapy and a letter of support before surgery (270). All were conducted within high-income specialist settings.

##### Physical outcomes, safety and adverse events

Analyses of large registry and health-system cohorts indicate that perioperative and 30-day postoperative safety for masculinizing chest surgery in patients under 18 is excellent, with complication rates around 4% and very low reoperation and readmission rates, comparable to those of older adolescents and young adults undergoing the same procedures (complications 4.2% in adolescents vs 4.1% in young adults; reoperation 1.6% vs 2.8%) (264–266). In a large single-surgeon series and an integrated health-system cohort with follow-up extending beyond one year, adolescents had lower overall complication rates (19% vs 42%), fewer revision surgeries (5.8% vs 16.3%), and higher satisfaction scores (mean 4.3/5 vs 3.6/5) than adults, with overall complication prevalence of 7.3% and revision rates of 10.9% in the health-system cohort (268,269).

Safety profiles for adolescents were consistently favourable across hospital-based case series and registry cohorts. In a pediatric academic hospital series of 65 minors undergoing chest reconstruction, hematoma occurred in 4%, airway events in 0.6%, and readmissions in 2.8%, with no major cardiopulmonary events reported (262). Registry-based comparisons of cisgender breast reduction and chest masculinization in adolescents found similar overall 30-day complication rates (about 4–5%), with no excess risk associated with gender-affirming chest surgery (266). Across these studies, major complications, unplanned readmissions, and returns to theatre in under-18 patients were rare—for example, hematoma reoperation rates of 2.8% in a national pediatric registry and 10.9% revision rates over longer-term follow-up in a large adolescent top-surgery cohort—supporting a consistent picture of low surgical risk for minors receiving masculinizing chest reconstruction (265,269,270).

##### Mental health and psychosocial wellbeing

Psychosocial outcomes for adolescents who received masculinizing chest surgery were consistently favourable over short to medium follow-up, with adolescent subsamples < 18 showing large reductions in chest dysphoria and improvements in gender congruence and body image (263,267,270,271). Among adolescents in the only study with an internal comparison group, those who underwent surgery had substantially greater improvements in chest dysphoria and appearance congruence at three months than matched adolescents who remained on surgical waitlists and did not receive surgery during the study period (263).

Qualitative interviews with transmasculine youth, most of whom had undergone chest surgery before age 18, described chest dysphoria as a major source of preoperative distress, including suicidality, and reported near-complete relief of chest dysphoria and marked gains in daily functioning, social participation, and comfort after surgery (267). In a follow-up survey of individuals who all received top surgery as minors, participants reported very low residual chest dysphoria, high gender congruence, and only minimal to mild anxiety and depression up to 7.6 years after surgery, with no reported regret among those operated under 18 (270).

##### Treatment trajectories and patient experience

Masculinising chest surgery for adolescents was typically embedded in multidisciplinary care pathways, with minors undergoing psychosocial assessment and gender-specialist evaluation before surgery, often alongside family involvement and, in some studies, at least one year of pre-surgical mental health therapy and a formal letter of support(267–271). Across prospective and longer-term follow-up cohorts that reported adolescent-specific data, young people consistently described gender-affirming masculinising chest surgery as increasing bodily congruence and enabling greater participation in school, sport, work, and social life (267,269–271)

Satisfaction with masculinising chest surgery was high in all studies that assessed it for adolescents and young adults, with marked improvements in chest dysphoria and body image and very low rates of regret. In the large integrated health-system cohort of 12–17-year-olds, documented regret was under 1% with no reversal surgeries, and satisfaction was recorded in over 90% of cases at up to 7 years’ follow-up (269). Qualitative interviews with transmasculine youth, most of whom had surgery before 18, reported near-total relief of chest dysphoria and no participants expressing regret (267). In prospective and post-operative survey cohorts including minors, adolescents reported sustained improvements in chest dysphoria and gender congruence, and no adolescent-operated participants agreed that they regretted surgery (270).

#### 4.3.3. Gender-affirming surgery within broader GAHC trajectories

Among the 15 studies examining surgery within broader GAHC pathways, adolescents were typically followed from puberty suppression into gender-affirming hormone therapy and, for a subset, later surgery, most often masculinising chest surgery, occurring in late adolescence or young adulthood (204,273,274).

##### Physical outcomes, safety and adverse events

Across longitudinal cohorts that included surgery within staged care, access to gender-affirming medical treatment (puberty suppression, hormones, and for some, surgery) was associated with improved appearance satisfaction and reduced gender-related distress, including better ratings of physical self and everyday functioning at follow-up compared with baseline (151,153,154). Although chest reconstruction was the most common surgery within these trajectories, one Dutch cohort assessed sexual functioning after vaginoplasty in transfeminine adults who had received puberty suppression and hormones in adolescence, finding that most participants could experience desire, arousal, and orgasm, and that the prevalence of sexual dysfunction was similar to that reported in transfeminine patients who began gender affirming treatment in adulthood, although a minority continued to report one or more sexual difficulties (234).

Safety findings in these broader-care cohorts were generally consistent with surgery-only series, with serious complications uncommon and no signal of difference in surgical risk attributable to earlier endocrine treatment stages (204,234).

##### Mental health and psychosocial wellbeing

Among the 15 studies where surgery occurred within broader gender-affirming care pathways, adolescents who progressed from puberty suppression and gender-affirming hormones to surgery—most commonly mastectomy, hysterectomy, or vaginoplasty in late adolescence or young adulthood—reported clear gains in appearance-related and global self-evaluation by follow-up (272,274). In Dutch longitudinal cohorts, young people who had completed puberty suppression, hormones, and surgery showed significant improvements in perceived physical appearance and global self-worth, while other psychosocial domains remained stable rather than deteriorating (272,275). Broader clinic and survey cohorts that included minors similarly found that adolescents who received desired surgeries within staged care reported high satisfaction with their bodies and care, alongside persistent frustration among those still waiting for surgery because of restrictive protocols, cost, or insurance barriers (187,273,279).

##### Treatment trajectories and patient experience

Studies that included surgery as part of broader gender-affirming pathways generally reported high continuity of care for adolescents, with most under-18s who started medical transition remaining engaged and few discontinuing treatment entirely; when care was interrupted, reasons more often reflected access barriers such as cost, insurance denials, travel distance, provider shortages, or parental consent constraints rather than loss of desire for gender-affirming treatment or surgery (187,275,279). Qualitative and mixed-methods work within these cohorts highlighted that adolescents considering or recovering from surgery relied heavily on multidisciplinary teams, affirming family members, and peer networks to navigate decision-making, manage expectations, and cope with postoperative recovery, and that gaps in any of these supports could delay or complicate access to desired surgical care (278–280).

#### 4.3.4. Findings from systematic reviews

Two grey-literature systematic reviews assessed outcomes of gender-affirming surgery in adolescents and young adults.

The Sax Institute Evidence Check for the New South Wales Ministry of Health (50) identified consistent postoperative improvements in gender dysphoria, body image, and psychosocial functioning, alongside low rates of short-term complications, predominantly for masculinising chest reconstruction (top surgery) in transmasculine and nonbinary youth. The review concluded that the certainty of evidence was low, reflecting reliance on observational cohorts and case series, short follow-up periods, small clinic-based samples, and heterogeneous outcome measures. It also highlighted the narrow geographic distribution of available studies (mostly from high-income countries) and the absence of data from low- and middle-income settings.

The RAND Corporation report (51) reported similar patterns: moderate- to high-certainty evidence for short-term surgical safety and low- to very-low-certainty evidence for psychosocial outcomes, with limitations driven primarily by study design rather than conflicting findings. As with Sax Institute Evidence Check, most included studies were drawn from high-income specialist clinics and involved predominantly masculinising chest surgery, with far fewer data on genital and other procedures.

We also note a third SEGM-commissioned systematic review and meta-analysis of masculinising chest surgery in individuals with gender dysphoria (281), which included too few participants under 18 for inclusion in this rapid review and is therefore not part of our main evidence base. Like all our included reviews, they were unable to perform meta-analyses on psychosocial outcomes because of heterogeneity in outcome measures and study designs. However, drawing on their pooled safety data for patients under 30, they found high-certainty evidence for very low rates of serious complications (including mortality, necrosis, and hypertrophic scarring), with rates equivalent to or below those reported in systematic reviews of reconstructive chest procedures in cisgender adolescents (162,163).

#### 4.3.5. Synthesis of surgical-care findings and guideline implications

The emerging evidence on surgical interventions in adolescents under 18 shows consistent patterns across multiple high-income settings and study designs, and remains broadly aligned with the 2021 GAHC guideline content on GAS. Masculinising chest surgery for medically eligible adolescents is associated with low short-term rates of serious surgical complications and with marked short-term improvements in chest dysphoria, body image, and daily functioning when delivered in multidisciplinary programmes (263,265). These findings are observed across cohorts using different observational designs, and although most follow-up periods remain limited, results to date are consistent and directionally aligned.

Systematic and registry-based analyses similarly report that major postoperative events (for example, reoperation for haematoma) are rare and similarly report very low rates of minor complications such as surgical-site infection or wound dehiscence, alongside substantial short-term improvements in gender-related distress and appearance satisfaction (265,268). Where age-stratified or external comparison groups are available, postoperative complication rates in adolescents under 18 are comparable to, or lower than, those reported in young adults or cisgender minors undergoing analogous breast procedures, supporting the view that adolescent chest surgery is at least as safe as adult practice when conducted in experienced centres (265,268).

Formal certainty ratings for psychosocial outcomes in systematic reviews are generally low to very low, reflecting the predominance of non-randomised and clinic-based studies rather than conflicting signals on benefit (263,265). The broader literature on paediatric gender-affirming care underscores the importance of sustained psychosocial support surrounding surgery, and documents how factors such as insurance coverage, geography, and stigma shape access pathways and waiting times (263,268). These access constraints, and the distress associated with prolonged delays, are likely to be particularly salient in South Africa and other settings with concentrated tertiary services and marked social inequities (51).

Taken together, the emerging evidence base indicates that masculinising chest surgery, when provided within comprehensive gender-affirming care pathways, has a favourable short-term safety profile and is consistently associated with meaningful reductions in chest dysphoria and improvements in psychosocial functioning for adolescents who receive it (263,268). While very long-term data in this small and marginalised population are still developing, the available studies point in a strongly beneficial direction for recipients of surgery and highlight the harms associated with barriers and delays to access of indicated interventions (51,265). Overall, the consistently positive short-term outcomes across multiple studies remain broadly aligned with the 2021 GAHC Guideline and affirm the central role of affirming and ongoing psychosocial support as the golden thread running through all aspects of gender-affirming healthcare for adolescents (51,263).

Table 7 maps the 2021 SAHCS GAHC surgical recommendations against the emerging evidence base, identifying areas of alignment and opportunities for refinement.

### 4.4. Non-medical gender-affirming practices

Non-medical gender affirming practices, including binding, tucking, packing and padding, are outlined in Chapter 4 (Non-medical gender-affirming practices) of the SAHCS GAHC Guideline (2021).

**SAHCS GAHC Guideline (2021):** Binding, packing, tucking and padding are common strategies used by TGD people to modify gender presentation, alleviate dysphoria and improve comfort in daily life. Packing and padding carry little to no health risk, while binding and tucking may lead to pain, skin problems and, for tucking, urinary or testicular symptoms. Providers are encouraged to discuss these practices with clients, offer harm-reduction guidance (such as avoiding unsafe materials and supporting rest periods), and approach them without pathologisation.

**The 2021 guideline does not include youth-specific recommendations**; content applies broadly across age groups, with no separate considerations for children or adolescents.

Nonmedical gender-affirming practices such as binding, tucking, packing and padding are used by many TGD young people to feel more comfortable in their bodies and safer in public spaces (27,282). *Binding* involves flattening chest tissue with specialised compression garments or other materials worn under clothing to achieve a more masculine appearance in the chest area. *Tucking* (also called *genital tucking*) refers to positioning the penis and testicles backwards, then holding them in place with firm underwear, gaffs or medical tape to create a flatter groin profile; testicles may or may not be guided upwards into the inguinal canal. *Packing* uses a prosthetic (homemade or commercially available) inside underwear or under clothing to create a penis-like, masculine bulge, while *padding* uses soft inserts or prosthetics (again, these may be homemade or commercially available) underneath clothing to create the appearance of more feminine breasts, hips or other body contours.

#### 4.4.1. Overview of the evidence base

Two recent sources address binding, packing, padding and tucking among TGD adolescents: one large online survey and one narrative review. A national cross-sectional online survey of 684 adolescents and young adults (13–24 years) in the United States focused on chest binding practices among transmasculine youth, including motivations and effects (283). The narrative review summarised 17 published studies and four online resources; it integrated adult data where youth-specific information was limited, but clearly marked this as extrapolation (27). Across both sources, binding was by far the most studied practice; evidence on tucking, packing and padding was thinner and mostly descriptive. No longitudinal or clinic-based monitoring studies were identified, likely reflecting the reality that these self-affirmation practices predominately occur outside formal care and are driven by TGD young people’s own needs for comfort, safety and gender congruence.

##### Findings from original research

The online survey from the USA found that young people bind because it helps them feel safer, reduces misgendering, and supports comfort in daily life (283). Most youth in the survey who had used binders reported some physical discomfort such as back or chest pain, overheating or shortness of breath, but continued binding because the emotional and social benefits were substantial. Among the survey population, chest dysphoria was linked with lower life satisfaction, while greater access to gender-affirming endocrine and/or surgical care correlated with improved wellbeing (283).

##### Findings from systematic reviews

The narrative review found that across included sources, binding and tucking generally improved comfort, confidence, and gender congruence among adolescents, reducing dysphoria, anxiety and the stress associated with misgendering (27). Reported downsides mostly comprised temporary discomfort such as skin irritation, pain, overheating, or itching, with rare reports of more severe adverse effects such as rib fracture or testicular torsion drawn largely from adult reports. Evidence for packing and padding suggests these practices carry negligible physical health risk and contribute positively to social ease and embodiment. (27). Across the review, the certainty of evidence was low due to cross-sectional designs and reliance on self-report, but the direction of findings was consistent: young people use these practices because they work for them, and yet clear, youth-appropriate safety information is often missing.

#### 4.4.4. Evidence synthesis and guideline implications

While limited, the available evidence affirms that nonmedical gender-affirming practices are meaningful tools that TGD adolescents might use to manage gender dysphoria, navigate public spaces, and feel more at ease in their bodies. For many, use of these practices is at least partly driven by lack of access to endocrine and/or surgical GAHC. There is no indication of serious risk from packing or padding. Physical discomfort associated with binding and tucking appears to be common but manageable when TGD youth have access to safer materials and guidance regarding rest periods, skin care, and warning signs for injury to the chest, back, ribs, testicles, or urinary tract. Clinicians should be familiar with common nonmedical gender-affirming practices that TGD adolescents might use, and prepared to offer non-judgemental support and guidance regarding safer practices. Where a young TGD person is binding or tucking in a way that causes or risks physical harm, exploration of possible gender-affirming medical intervention is indicated in addition to guidance regarding safety.

The current evidence base on non-medical gender-affirming practices leaves clear gaps around developmental considerations, optimal guidance for safe practice of binding and tucking, and strategies to address barriers that limit access to medical and surgical GAHC. Our collective community and clinical experience affirms that these practices are not uncommon among South African TGD adolescents, and clinicians should therefore remain aware of safety considerations and be prepared to offer supportive guidance.

### 4.5. Policy and legal interventions

Although policy and legal interventions are not addressed as a standalone area in the SAHCS Guideline (2021), the rapid review identified several studies examining how laws, regulations, and administrative systems shape health and wellbeing outcomes for TGD children and adolescents. This section summarises that emerging evidence.

#### 4.5.1. Overview of the evidence base

Eight original research studies examined relationships between policy or legal environments and health outcomes for TGD adolescents (149,284–290). No systematic reviews were identified in this domain. The studies were conducted predominantly in high-income contexts — including the United States, United Kingdom, Aotearoa/New Zealand, and Australia — and employed cross-sectional surveys, qualitative designs, and observational cohort analyses.

Methodological limitations include the use of non-probability samples, reliance on self-reported outcomes, and predominantly cross-sectional designs. Strengths include several large national datasets, rich qualitative accounts from adolescents and caregivers, and policy-linked analyses that paired individual-level wellbeing measures with structural indicators.

We present the findings by nature of policy exposure: Access restrictions and bans on gender-affirming care; affirming and protective policy frameworks; and legal recognition and identification rights.

#### 4.5.2. Restrictions and bans on gender-affirming care

Across multiple contexts, national- and state-level restrictions on gender-affirming care were consistently associated with worsening mental-health outcomes among adolescents(284,287). A study in the UK documented rapid deterioration in wellbeing following the national ban on puberty pausers for TGD youth, with young people reporting escalating anxiety, depression, self-harm, and school withdrawal (287). Families described substantial distress linked to disrupted care pathways; those who obtained treatment abroad often reported more consistent care and reduced psychological distress (287).

Similar patterns were observed in the United States. Both proposed and enacted state-level care bans were described by youth and caregivers as contributing to or worsening heightened depression, anxiety, and suicidality among affected adolescents (284,285). Young people and caregivers described interrupted treatment, increased stigma, and pressures to relocate to maintain access to care (284,285). Some families responded through collective action or advocacy, reflecting both the psychosocial toll of policy uncertainty and the mobilisation it prompted (285).

Evidence from Aotearoa/New Zealand highlights how system-level barriers function as structural determinants of health (289). National survey data from TGD youth show that those with unmet needs for gender-affirming care – whether due to cost, age restrictions, or service shortages – had significantly higher psychological distress, with adolescents aged 14–17 particularly likely to report unmet need for hormones; in the overall youth sample, those with unmet need for hormones had more than double the odds of suicide attempts compared with peers who were able to access care (289). These results suggest that administrative or capacity-related obstacles can have impacts similar to those of formal bans.

Cross-country qualitative evidence highlights how national and regional policy environments shape access to care (149). Youth in Canada and Australia, where access to puberty suppression and hormone therapy was comparatively better, described improved mental health, reduced dysphoria, and increased optimism when able to obtain care (149). In contrast, adolescents in Switzerland and England, where regulatory restrictions, long waiting lists, and administrative hurdles limited access, reported heightened distress, hopelessness, self-harm, and suicidality. Across all settings, young people identified system-level delays and gatekeeping as primary sources of psychological harm (149).

#### 4.5.3. Protective and affirming policy environments

Population-level analyses indicate that affirming legal and policy environments correspond with more favourable mental-health and behavioural outcomes for TGD adolescents (288). A multi-state analysis of the U.S. Youth Risk Behavior Survey data found that TGD adolescents living in states with explicit anti-discrimination protections or inclusive school sport participation guidance had significantly lower odds of depression and cigarette use compared with peers in states lacking such protections (288). These results point to the buffering role that protective policy frameworks can play against minority stress processes.

A secondary analysis of the U.S. Transgender Survey compared adults who had accessed gender-affirming medical care during adolescence with those who had not, finding that adolescent treatment exposure was associated with lower odds of severe psychological distress in adulthood, with these associations moderated by state-level transgender policy climates (290). Patterns of past-year healthcare avoidance also varied by policy environment: avoidance was higher overall among those with adolescent treatment exposure, but this effect was substantially reduced in more supportive states (290). Together, the evidence suggests that legal and political contexts shape both access to care and the longer-term mental-health impacts of adolescent treatment.

#### 4.5.4. Legal gender recognition and identification rights

Access to gender-congruent identification documents appears to play a meaningful role in the mental health of TGD youth. A national U.S. survey of 6,581 TGD and non-binary youth found that adolescents unable to update the gender marker on their legal identification had approximately double the odds of a past-year suicide attempt compared with those whose documents reflected their gender (286). Elevated risk was also observed among youth who were eligible to update documents but had not yet done so. These associations remained significant after adjusting for family support, income, and access to gender-affirming medical care, suggesting that bureaucratic barriers themselves may contribute to psychological harm (286).The evidence indicates that legal recognition functions as a structural form of affirmation, with implications for emotional safety, social participation, and wellbeing (286).

#### 4.5.5. Policy evidence synthesis and guideline implications

The identified studies show that restrictive laws and systemic barriers – whether outright healthcare bans or administrative obstacles – are consistently linked, in both qualitative and quantitative studies, to heightened depression, anxiety, suicidality, and social isolation among TGD youth(149,284–287,289). Conversely, affirming policy frameworks, anti-discrimination protections, and accessible legal gender recognition are associated with improved mental-health indicators and, in some analyses, lower substance use and other risk behaviours, as well as enhanced wellbeing (286,288,290). Parents’ accounts mirror these findings, describing how proposed or enacted care bans intensified depression, anxiety, suicidality, and family distress among affected adolescents (284,285,287).

Although the SAHCS Guideline (2021) does not include a dedicated chapter on policy or legal determinants, the emerging evidence underscores their relevance for clinical practice and advocacy. Health professionals play a critical role not only in providing affirming care, but also in anticipating and helping mitigate the harms of hostile policy environments – through anticipatory guidance, psychosocial support, and policy engagement. Ensuring equitable access to GAHC thus requires attention to the broader structural conditions that enable or obstruct care.

## 5. Discussion

### 5.1. Summary of key findings

The global evidence on GAHC for TGD children and adolescents has expanded rapidly since the publication of the SAHCS GAHC Guideline in 2021.

Across psychosocial, endocrine, surgical, non-medical and policy interventions, the evidence from 2021–2025 presents a coherent picture: when TGD children and adolescents receive GAHC within supportive social, clinical, and policy environments, outcomes are consistently stable or improved, and harms are rare. Moreover, the evidence consistently links non-clinical delays in providing GAHC to worsening distress and poorer mental-health outcomes.

Key findings across each intervention domain are summarised below.

**Across diverse contexts, psychosocial interventions** are consistently associated with meaningful reductions in distress, anxiety, and suicidality. These interventions include support for social transition, family involvement, safer school environments, and neurodiversity-informed supports that recognise sensory needs, communication differences, and processing styles. Affirming psychosocial interventions are also linked to improvements in emotional regulation, resilience, belonging, school participation, and day-to-day functioning. No study reported harms arising from affirming psychosocial care. In contrast, practices that delay, withhold, or discourage affirmation, including identity change efforts, are consistently linked to psychological harm. Evidence shows that psychosocial care is not ancillary: it is a core component of gender-affirming healthcare for TGD young people, especially in contexts shaped by structural violence, poverty, stigma, and limited access to mental-health resources.

**Endocrine interventions** – including puberty-pausing medication and gender-affirming hormone therapy – consistently produce the expected and desired physiological effects under specialist monitoring. Adverse events are generally mild, reversible, and aligned with known paediatric endocrine profiles. Mental-health outcomes during puberty suppression and gender-affirming hormone therapy range from neutral to improved, with consistent evidence that timely access, rather than prolonged non-clinical delay, is associated with lower suicidality, improved mood, enhanced quality of life, and greater appearance congruence and functioning. Adolescents who receive endocrine care show high treatment satisfaction, high continuation into adulthood, and very low rates of regret. Menstrual suppression is a safe, effective, and highly valued component of care for TGD adolescents who menstruate, resulting in high amenorrhoea rates, reduced pain and menstrual-related dysphoria, and strong overall satisfaction. Fertility preservation procedures appear feasible and generally safe for adolescents, underscoring the importance of early, developmentally appropriate fertility counselling, although accessibility is often constrained by cost, dysphoria related to procedures, and timing of referral.

**Evidence on surgical interventions** for adolescents under 18 is limited but highly consistent, focusing almost exclusively on masculinising chest reconstruction – the only gender-affirming surgical procedure routinely accessed by minors. Across clinical settings, chest surgery performed within multidisciplinary programmes shows very low rates of complications and surgical revisions, with safety profiles comparable to or better than those observed in adult or cisgender comparison groups undergoing analogous breast procedures. Psychosocial outcomes are consistently positive, including improved body image, reduced dysphoria, and increased participation in social, educational, and physical activities, accompanied by high levels of patient satisfaction. Regret is exceedingly rare, even in cohorts with longer-term follow-up. Evidence on other types of adolescent gender-affirming surgeries remains extremely limited, with only isolated cases of genital procedures documented in registry data and no primary studies reporting outcomes for feminising genital surgery in minors. Overall, when delivered within comprehensive, affirming care pathways, masculinising chest surgery demonstrates reliable short-term safety and meaningful psychosocial benefit for eligible adolescents.

**Evidence on non-medical gender-affirming practices** – such as binding, tucking, packing, and padding – is limited but consistently shows that these practices help many TGD adolescents manage dysphoria and navigate daily life, particularly where access to medical care is limited. Packing and padding appear low-risk; discomfort from binding and tucking is common but usually manageable with safer materials, rest periods, and clear guidance on warning signs. Clinicians should be familiar with these practices and offer non-judgemental, practical advice, with exploration of medical options where harm is occurring or likely. Despite evidence gaps, community and clinical experience – including in South Africa – indicates that these practices are common and that supportive, evidence-informed guidance remains important.

**Policy and legal environments** have a measurable impact on the wellbeing of TGD children and adolescents. Restrictive or hostile settings – such as healthcare bans, administrative barriers, or exclusionary school policies – are consistently associated with increased distress, self-harm, suicidality, social withdrawal, disrupted care, and family strain. Conversely, protective policy frameworks, including anti-discrimination regulations, inclusive school protocols, and accessible legal gender recognition, are linked to improved mental-health outcomes, lower risk behaviours, and enhanced wellbeing. These findings demonstrate that healthcare outcomes are shaped not only by clinical interventions but by the broader social, legal, and institutional conditions that enable or obstruct access to affirming care.

**Taken together, the evidence base shows consistent patterns across diverse study designs and contexts: gender-affirming psychosocial, endocrine, and surgical interventions, as well as supportive policy and non-medical practices**, are associated with improved wellbeing and functioning for young people when provided within multidisciplinary, supportive systems. In contrast, non-clinical delays in providing care, including policy environments that curtail or complicate access, and non-affirming practices generate clear risks. Although methodological limitations constrain certainty of evidence, the direction and consistency of findings across domains reinforce the safety, benefits, and clinical relevance of affirming care for TGD children and adolescents.

### 5.2. Strengths and limitations of this rapid review

The review has several important strengths. First, it was conceived and led from the Global South by a queer- and trans-led team with combined methodological, clinical, and lived-experience expertise. This positionality shaped decisions about relevance, contextualisation, and equity, and helped ensure that the synthesis remained grounded in the legal, social, and service realities of TGD youth in South Africa rather than importing assumptions from high-income settings. To our knowledge, this is the first systematic synthesis of GAHC evidence for young people under 18 led from the Global South.

Second, the search strategy was broad and intentionally inclusive. We used a comprehensive multi-database search via EBSCOHost with no language restrictions, deliberately incorporating both current and pathologising terminology for TGD identities and interventions (288). Searches were run in two waves (2021–2024 and 2025) to capture the fast-moving post-2021 evidence base. In addition, we conducted structured LLM-assisted searches specifically for systematic reviews (including grey-literature reviews) and manually verified eligibility and methods for all suggested sources. This approach yielded 200 eligible primary studies and 33 systematic reviews (including four grey-literature systematic reviews). Cross-mapping of inclusions showed that 91 of our 200 primary studies had not been included in any of the systematic reviews identified in this project, substantially extending the evidence base available for synthesis and highlighting gaps in existing secondary reviews.

Data extraction and synthesis followed a structured and transparent process. We developed tailored Airtable extraction tools for primary studies and systematic reviews, piloted them across multiple reviewers, and used pre-specified fields to capture study characteristics, populations, interventions, outcomes, and contextual factors in alignment with rapid-review guidance on team roles, study selection, data extraction, and risk of bias (93). LLMs were used in a limited, supportive role to generate draft extractions and cross-check narrative summaries against the underlying data; all such outputs were line-checked against the original reports and manually corrected before being entered into the database or incorporated into the synthesis.

As is typical for rapid reviews, several methodological streamlining decisions were made. After an initial dual-screening calibration phase, title and abstract screening proceeded with a single reviewer, with borderline or uncertain records flagged for second-reviewer input at abstract or full-text stage. Full-text screening and data extraction were not conducted in duplicate [see team and study-selection guidance: (93). We did not conduct a de novo, outcome-by-outcome risk-of-bias or GRADE/CERQual certainty assessment; instead, we drew on existing appraisals from previous systematic reviews where available and treated them as a secondary interpretive resource rather than as formal ratings for this review (93). No meta-analysis was attempted due to the extreme heterogeneity of designs, interventions, and outcomes, and the review was not prospectively registered in a database such as PROSPERO. These choices are consistent with published guidance on rapid reviews, but they inevitably reduce the granularity of quality assessment and may increase susceptibility to subjective judgements compared with a fully resourced systematic review (89).

Other limitations arise from the scope and available evidence. Our focus on empirical studies with at least five TGD participants under 18 excluded descriptive and purely attitudinal work, as well as case reports and very small case series, which may contain clinically relevant signals but do not provide robust outcome data. The requirement for either disaggregated data for under-18s or predominantly adolescent samples led to exclusion of some mixed-age studies that could not be reliably interpreted for youth. Evidence is heavily concentrated in specialist clinic and high-income settings, with very limited South African or broader LMIC data, constraining the direct generalisability of findings to local service contexts, particularly outside urban tertiary centres.

Overall, while the rapid-review design imposed necessary methodological constraints, the breadth of the search, the systematic cross-linking of primary studies and prior reviews, the large number of newly identified studies, and the structured, team-based narrative synthesis all support the robustness and practical value of the findings. The review should therefore be understood as a transparent, contextually grounded rapid synthesis rather than a definitive, fully exhaustive systematic review, and its conclusions should be interpreted with appropriate caution and attention to the underlying evidentiary limitations.

### 5.3. Strengths and limitations of the evidence base covered in this report

The evidence assembled in this rapid review—200 primary studies, 29 academic systematic reviews, and four rigorous technical reports—shows a consistent pattern across psychosocial, endocrine, surgical, non-medical, and policy interventions. When TGD young people access affirming psychosocial support, timely endocrine care, and, for older adolescents who need it, surgical interventions, improvements are seen in mental health, dysphoria, and day-to-day functioning. Serious adverse events remain rare. Systematic reviews published since 2021—including those commissioned in conservative policy environments—echo these findings and report no evidence of population-level harm. This consistency across diverse study designs strengthens confidence in the overall direction of effect, even where formal certainty ratings remain low.

Important limitations temper the strength of the evidence. Most primary studies are observational and/or clinic-based, with small, non-representative samples, short follow-up, and limited geographic diversity. Randomised or quasi-experimental designs are scarce because they are typically neither feasible nor ethical. Comparison groups are often absent, and the field lacks consensus on appropriate comparators—other TGD youth receiving different interventions (or waitlisted controls), cisgender youth of the same sex assigned at birth, or cisgender youth of the affirmed gender. These unresolved methodological issues complicate estimation of effect size and strength. Many cohorts draw repeatedly on the same Dutch and U.S. clinical populations, giving an inflated sense of evidence volume.

Follow-up periods are generally short (6–36 months for psychosocial and endocrine outcomes; 6–12 months for surgical outcomes), constraining understanding of long-term trajectories into adulthood. Evidence for endocrine and surgical care relies heavily on retrospective chart reviews, with variable data quality, inconsistent outcome recording, and limited use of patient-reported measures. Heterogeneity in interventions accessed by individual TGD youth further limits precision.

Measurement heterogeneity compounds these challenges. Mental-health and psychosocial studies use diverse instruments and time-points, often without reporting baseline functioning, prior treatment exposure, or contextual stressors such as family or school hostility. Few studies include validated measures of everyday functioning or safety. Adverse events are inconsistently reported outside expected endocrine effects (e.g., transient bone-density or haematocrit changes) or rare short-term surgical complications.

Geographically, nearly all clinical evidence comes from high-income settings—most commonly the United States, the Netherlands, the United Kingdom, Australia, and Canada—with almost no primary research from the Global South. Across domains, key populations remain under-represented or insufficiently disaggregated, including non-binary youth, neurodivergent adolescents, and young people across diverse racial, ethnic, socioeconomic, and rural contexts.

Systematic reviews reflect these same constraints. AMSTAR-2 and GRADE assessments consistently rate certainty as low to very low due to design limitations, small samples, overlapping cohorts, short follow-up, and narrow geographic scope—not conflicting findings. Despite these limitations, the evidence base is coherent and internally consistent: TGD young people experience short- to medium-term improvements in mental health, dysphoria, and functioning when they receive affirming care, with low rates of serious adverse events. The gaps underscore the need for more rigorous, longer-term, inclusive research—especially from the Global South, and from South Africa in particular—to better characterise evolving health needs and support equitable health-system planning.

#### GRADE Evidence to Decision Framework, Low-Certainty Evidence, and South African Values

The certainty of the evidence surrounding GAHC for youth must be understood in the broader reality that low and very low certainty evidence underpins a substantial proportion of Western medical practice overall (291–293). This is especially true in paediatrics, where ethical constraints routinely preclude randomised trials and contribute to major inconsistencies across the evidence base for even common conditions such as asthma(293). A 2024 analysis by the American Academy of Pediatrics found that only 10.6% of their own practice recommendations were based on the highest-quality evidence (291). High-certainty evidence is scientifically ideal but often pragmatically out of reach; insisting on it as the only acceptable basis for clinical recommendations risks entrenching health inequities for small, marginalised, and hard-to-reach populations(294,295), including TGD youth.

The recently updated GRADE Evidence-to-Decision (EtD) framework directly acknowledges this reality. As clarified in the 2025 Core GRADE 7 update(296), low-certainty evidence typically warrants a conditional recommendation which is an explicit signal that clinicians must engage meaningfully in shared decision-making that centres patient values. Prof Gordon Guyatt, widely regarded as the “godfather of evidence-based medicine” and the architect of the GRADE framework (297), has been unequivocal about the misuse of GRADE in policy advocacy against GAHC for youth (297,298). As he and other coauthors on some of the systematic reviews cited in this rapid review(48,49,281) have recently written:

It is profoundly misguided to cast health care based on low-certainty evidence as bad care or as care driven by ideology, and low-certainty evidence as bad science. Many of the interventions we offer are based on low certainty evidence, and enlightened individuals often legitimately and wisely choose such interventions. Thus, forbidding delivery of gender-affirming care and limiting medical management options on the basis of low certainty evidence is a clear violation of the principles of evidence-based shared decision-making and is unconscionable. The appropriate use of our work is in ensuring patients receive needed care and in helping TGD patients and their clinicians in decision making.

We write this in the hope that all those who use our work to inform the care of TGD patients receiving gender-affirming care, and those using our work in consideration of policy decisions, prioritize the delivery of compassionate and conscientious care that fully respects the autonomy of the TGD patient. Gordon Guyatt et al 2025 (https://hei.healthsci.mcmaster.ca/systematic-reviews-related-to-gender-affirming-care/)

In South Africa, this is not simply a methodological point; it is a constitutional and statutory imperative. The Batho Pele principles mandate consultation and the prioritisation of people’s lived realities. Section 129 of the Children’s Act requires respect for adolescents’ evolving capacity to participate meaningfully in decisions about their own healthcare. When the overall evidence base offers low scientific certainty yet points consistently in a particular direction, the GRADE EtD framework requires clinicians to centre the patients’ values, preferences, and lived experience—not to impose blanket restrictions or personal disapproval framed as clinical caution. This approach aligns squarely with the informed-consent model endorsed in South African and global clinical guidelines on GAHC for youth(2,55).

### 5.4. Implications for clinical practice

In addition to the intervention domain-specific comparisons with the 2021 SAHCS GAHC Guideline presented earlier in the report, the evidence has broader clinical implications that cut across intervention areas and highlight principles essential for delivering safe, effective, and contextually grounded care.

#### Affirmation remains central to safe and effective care

Evidence across psychosocial, endocrine, and surgical domains consistently shows that affirming interventions are associated with improved mental-health outcomes, reduced dysphoria, and better daily functioning for children and adolescents. Practices that withhold affirmation or seek to modify a young person’s gender identity fall outside recognised standards of care and are associated with harm (55). In the South African context, affirmation is both a clinical imperative and a rights-based obligation grounded in constitutional commitments to dignity, equality, and non-discrimination.

#### Imposed non-clinical delays result in harm

Across the evidence base, avoidable delays – including administrative barriers, resource shortages, and long waitlists – are consistently associated with worsened mood, heightened distress, and increased suicidality. Minimising non-clinical delays is therefore not only operationally desirable but a core component of harm reduction.

#### Multidisciplinary, coordinated care underpins good outcomes

The strongest outcomes occur where mental-health, endocrine, and surgical teams work collaboratively, with structured information-sharing, ‘warm’ handovers, and consistent follow-up. Multidisciplinary teams improve outcomes across both medical and psychosocial domains, yet fragmentation, workforce shortages, and inconsistent record-keeping remain major obstacles in South Africa (73). Strengthening referral pathways, documentation systems, and cross-disciplinary communication is essential for continuity and safety.

#### Timely access to endocrine care improves wellbeing

Endocrine interventions for TGD adolescents show predictable physiological effects and broadly favourable psychosocial outcomes when delivered through specialist care and without avoidable delay. Combined-pathway data indicate that prolonged gaps between puberty suppression and GAHT worsen psychosocial trajectories in adolescents seeking these treatments. Clinical practice should anticipate risks of stock-outs, plan sequencing and monitoring proactively, and provide early, developmentally appropriate fertility counselling. Fertility counselling is an important component of comprehensive sexual and reproductive healthcare and supports adolescents’ long-term reproductive autonomy.

#### Psychosocial support is required across all stages of care

Even when medical interventions alleviate dysphoria, TGD adolescents continue to face minority stress, family conflict, school-based harassment, and system disruptions, all of which shape their mental-health trajectories (60,61). Ongoing counselling, parent/caregiver support, and collaboration with schools and community services are therefore critical. Mental-health comorbidities should prompt supportive management – not be used to delay or deny medically indicated gender-affirming care.

#### Affirming families and caregivers are central to positive outcomes across all intervention domains

Evidence consistently links supportive family environments with improved mental-health outcomes, reduced distress, sustained engagement in care, and improvements in wellbeing and daily functioning. Clinical practice should therefore include structured caregiver support, guidance for parents, and participatory, collaborative consent processes that foster affirming family relationships and strengthen the relational ecosystems that enable TGD children and adolescents to thrive.

#### Neurodiversity-informed practice should be routine

Evidence shows that neurodiversity-informed supports — including structured environments, concrete communication, sensory accommodations, and collaborative goal-setting — are associated with improved participation, emotional regulation, and overall functioning for neurodivergent TGD youth. These approaches should be integrated into routine assessment, consent processes, and psychosocial support, rather than added only when difficulties emerge. Consistent with findings that co-occurring mental-health conditions and developmental differences are common yet compatible with positive outcomes, neurodivergence should not be treated as a contraindication or a reason to delay medically indicated gender-affirming care.

#### Menstrual suppression should be offered proactively as part of gender-affirming care

For adolescents who menstruate, menstrual suppression provides reliable relief from dysphoria and menstrual-related pain, with favourable safety profiles across methods. Clinicians should initiate conversations about options, expected bleeding patterns, side-effects, and integration with GAHT and reproductive-health planning. Menstrual suppression may also support adolescents with comorbid reproductive health conditions and may improve wellbeing in contexts marked by period poverty (288).

#### Surgical care requires structured preparation, psychosocial support, and equitable access pathways

Masculinising chest reconstruction demonstrates high satisfaction and low complication rates when provided within multidisciplinary care. Optimal outcomes require comprehensive pre-operative counselling, family involvement, and postoperative psychosocial support. Provincial disparities, long waitlists, and cost barriers mean clinicians should also support adolescents through the psychological impacts of delays and provide accurate information and safety planning.

#### Equitable access is foundational to achieving positive clinical outcomes

Barriers such as long waitlists, geographic inequities, and inconsistent service availability can undermine care and worsen outcomes. Strengthening referral pathways, coordinating across public and private sectors, and decentralising components of care (e.g., menstrual suppression, psychosocial support, aspects of endocrine monitoring) can improve outcomes even in resource-constrained settings.

Overall, the evidence supports affirming, youth-led, multidisciplinary care with attention to timing, context, and equity. Clinicians should work relationally – with families, schools, and community networks – to build the supportive ecosystems the evidence shows are central to wellbeing.

### 5.5. Implications for policy and health systems

The evidence has several implications for policy and health-system design in South Africa. These implications highlight the structural conditions required to ensure safe, equitable, and effective gender-affirming care for TGD children and adolescents.

#### Restrictive policies cause measurable harm

The evidence shows that bans on gender-affirming care, administrative barriers, and service shutdowns are associated with increased depression, anxiety, suicidality, and social withdrawal among adolescents. These harms can emerge even before implementation, reflecting the psychological impact of policy uncertainty, stigma, hostile public discourse, and disrupted care pathways. Opaque referral structures, administrative hurdles, and exclusions from medical-aid benefits function as de facto barriers even in the absence of explicit policy restrictions (73). Policy instability itself becomes a source of minority stress and can undermine trust in health services.

#### Supportive policies strengthen wellbeing at population level

Affirming policy environments – such as anti-discrimination protections, inclusive school policies, and accessible legal gender recognition – correspond with lower psychological distress, reduced suicidality, and healthier risk behaviours among adolescents. These policies operate as structural buffers, supporting resilience and reducing demands on clinical services. They also reduce the burden on mental-health and emergency services, underscoring the value of maintaining and strengthening South Africa’s constitutional and legislative protections.

#### Adolescent health services should be strengthened to meet the needs of TGD youth

Evidence demonstrates that gender-affirming care improves mental health, reduces suicidality, and supports school engagement and social participation. While endocrine and surgical interventions for young people are specialist-delivered, many components of gender-affirming care – such as psychosocial support for young people and their families, menstrual suppression, trauma-informed counselling, management of co-occurring mental-health conditions, and safe referral pathways – can and should be integrated into routine adolescent health and mental-health services. Doing so aligns with national priorities on SRHR, HIV prevention, youth mental health, and adolescent-friendly service delivery (63).

#### Health-system design directly affects clinical outcomes

System-level barriers – including medication stock-outs, long waitlists, provider shortages, and the concentration of services within a few tertiary centres – shape treatment trajectories and contribute to avoidable distress (72,97). These challenges are intensified by the uneven provincial distribution of expertise and the absence of standardised referral pathways, which leave many adolescents without feasible routes into care (67,77).

Policy action should prioritise reducing non-clinical delays through:

- strengthened referral pathways between primary care, mental-health services, and specialist endocrine programmes
- improved procurement and stock-management systems
- transparent and equitable waitlist processes
- targeted training to expand the pool of clinicians able to participate in multidisciplinary care
- standardised documentation and interoperable health records to support continuity of care
- mechanisms for community and youth participation so that TGD adolescents and caregivers have formal channels to inform service design and oversight

These measures align service delivery with the Constitution, the National Health Act, and the Children’s Act, which already establish the legal foundation for this care. They also reflect Batho Pele obligations to provide accessible, equitable, dignified, and transparent services, particularly for populations facing ongoing marginalisation and stigmatisation.

#### Equitable financing is necessary to reduce care disparities

Long waitlists, high private-sector costs, and provincial disparities shape who can access gender-affirming care and when (67,72). Exclusion of gender-affirming care from many medical-aid benefits further entrenches inequities, disproportionately affecting families with limited financial resources. Policy mechanisms should include:

- access to, and funding for, multidisciplinary care teams
- strengthening reliable access to puberty pausers, GAHT, and menstrual suppression within existing essential medicines frameworks
- financing for training, supervision, and workforce development
- expansion of public-sector surgical capacity
- routine monitoring of wait times and geographic distribution of services

These measures would reduce inequities between provinces, between public- and private-sector services, across socioeconomic groups, and between rural and urban areas. Financing decisions should also anticipate the harms of delayed care – which are well documented in the evidence base – and treat timely access as a matter of health equity and cost-effective prevention, given the downstream impacts of unmanaged distress, disruptions to schooling, and avoidable emergency mental-health care.

### 5.6. Priorities for future research

#### Growing the evidence base in low- and middle-income countries

Almost all the research evidence synthesised in this review originates in high-income settings. There is an urgent need for research from South Africa and other low- and middle-income countries to understand how resource constraints, workforce shortages, stock-outs, fragmented referral pathways, and geographic inequities affect care outcomes and feasibility. This gap reflects not a lack of local expertise, but a lack of coordinated resourcing and investment. South Africa has the clinical and research capacity to lead in this field; what is missing is infrastructure, dedicated funding mechanisms, and long-term institutional support to generate locally grounded evidence that reflects our social, legal, and health-system realities.

#### Expanding long-term follow-up into adulthood

Few studies identified in our review extend beyond early adulthood. Long-term outcomes related to bone health, cardiometabolic risk, neurodevelopmental trajectories, sexual wellbeing, fertility decisions, and psychosocial functioning remain insufficiently characterised. Locally informed cohorts and strengthened routine data systems would enable more accurate understanding of long-term safety and effectiveness.

#### Improving visibility of non-binary adolescents in research

Most studies include non-binary adolescents but do not disaggregate their data, obscuring the specific experiences, needs, and outcomes of this group. Research should explicitly examine diverse hormonal goals, psychosocial pathways, embodiment needs, and models of care for non-binary adolescents. Tools and outcome measures must also be validated or adapted to capture non-binary experiences meaningfully.

#### Investigating structural determinants and intersectional factors

Race, class, rurality, disability, neurodiversity, and other determinants are rarely analysed in depth. South African treatment realities cannot be understood without intersectional analysis. Future research should also examine how structural barriers – including long waitlists, administrative hurdles, medical-aid exclusions, transport barriers, and variable service availability – shape treatment timing, wellbeing, and long-term outcomes.

#### Strengthening evidence on menstrual suppression and adolescent fertility pathways

The emerging menstrual-suppression literature is promising but small and methodologically limited. Research is also needed on adolescents’ fertility preferences, decision-making processes, reproductive intentions, and experiences with fertility preservation, including how cost, dysphoria, and timing of referral influence uptake.

#### Expanding co-produced research and participatory approaches

Adolescents’ voices remain under-represented, particularly in endocrine and surgical domains. Participatory and co-designed studies are essential for understanding treatment goals, definitions of successful outcomes, decision-making needs, and experiences of care. Research ethics processes must better recognise community expertise and support youth collaboration in shaping research questions, methods, and dissemination.

### 5.7. Conclusion

The findings from this rapid review demonstrate that, amid growing global and local opposition to GAHC for young people, the SAHCS GAHC Guideline remains a robust, evidence-informed framework grounded in constitutional commitments to dignity, equality, and non-discrimination. Evidence published since 2021 strengthens rather than challenges the Guideline: across psychosocial, endocrine, surgical, and policy domains, findings from 2021–2025 consistently show that gender-affirming care improves wellbeing, prevents harm, and supports healthier developmental and mental-health trajectories when delivered within supportive clinical, social, and policy environments. While most available studies are observational, this reflects ethical and methodological realities of paediatric research and interventions that cannot be ethically randomised or withheld, rather than evidentiary weakness; consistent findings across diverse settings provide compelling, real-world evidence of effectiveness and safety. Attempts to impose unrealistic standards of proof on care for TGD youth are therefore political rather than scientific. Taken together, the evidence provides a clear mandate: reducing non-clinical delays, expanding access, and strengthening the enabling conditions for care are urgent public-health and human-rights priorities. This review is both an affirmation of the SAHCS Guideline and a call to action to ensure that all TGD young people in South Africa can access the care that supports their wellbeing, autonomy, and inherent dignity.

## Supplementary Material

Supplementary Files

This is a list of supplementary files associated with this preprint. Click to download.

- DataAppendixFullDatasetsforYouthGAHCRapidReviewversion1.0.1.xlsx

## Figures and Tables

**Figure 1:**
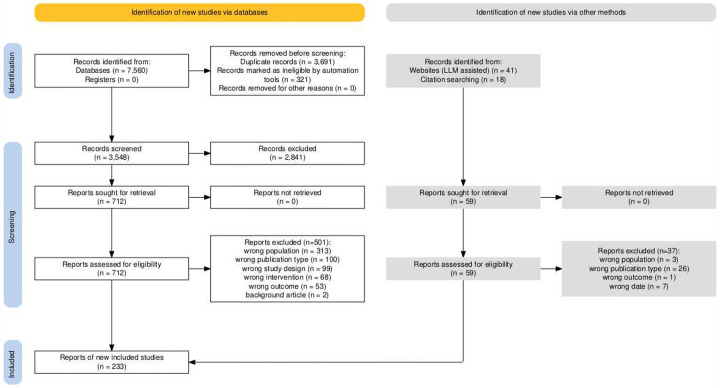
PRISMA diagram showing flow of literature searches, exclusions and inclusions of retrieved sources

**Table 1:** Key constitutional, statutory, and policy instruments relevant to adolescent GAHC in South Africa

Instrument	Provisions relevant to adolescent GAHC	Implications for practice
Constitution (Sections 9, 10, 27, 28)	Equality, dignity, access to healthcare; best interests of the child paramount	Duty to provide equitable, non-discriminatory access to needed care, including for TGD youth
Batho Pele principles	Consultation, access, information, transparency, redress	Patient-centred, respectful services; clear information; responsive complaints pathways
Children’s Act	≥12s may consent to medical treatment if sufficiently mature; surgical treatment requires child’s consent (if capable) and parental/guardian assistance	Clear consent routes for puberty-pausing medications/GAHT; additional safeguards for surgery; pathways where parental support is absent should be available
UN Convention on the Rights of the Child	Right to health, non-discrimination, evolving capacities, best interests	Consider the adolescent’s views and maturity; avoid discriminatory barriers; prioritise best interests
National Contraception & Fertility Planning Policy (2019)	Person-centred, differentiated service delivery; coordination of services; fertility planning	Integrate contraception and fertility counselling in GAHC as indicated; support decentralised, tailored hormonal care
NSP for HIV, TB & STIs (2023–2028)	Expanded coverage for key populations; integrated, equitable services; geographic redistribution	Emphasises equity, integration, and accessibility of services across geographic areas
National SRHR Policy (2021)	Comprehensive SRHR for adolescents and youth; services must be non-judgmental, confidential, private, accessible	Embeds adolescent-friendly standards in GAHC delivery; highlights confidentiality and convenient access
The South African Schools Act 84 of 1996	Safe, supportive school environments; anti-bullying measures	Emphasises safety, inclusivity, and protection from discrimination in educational settings

**Table 2:** PICOT map of eligibility criteria for included studies

Domain	Criteria
*Population*	TGD youth under 18 ***or*** family units including TGD youth under 18. Minimum N = 5.
*Intervention / exposure*	Any type of psychosocial or medical care, support, policy, practice or deliberate harm ***delivered to or targeting TGD youth under age 18*** across all socioecological levels (individual, dyadic/familial, institutional, policy/legal, sociocultural).
*Comparator / control*	Any or none (not required).
*Outcomes*	Any empirically assessed health or psychosocial outcome(s)
*Time*	Any follow-up time frame allowed, including both prospective or retrospective assessment.
*Study types*	Quantitative, qualitative, mixed-methods, including prospective and retrospective designs. Systematic reviews eligible if based on transparent, reproducible search and screening protocols.

**Table 3:** Summary of evidence and guideline implications for psychosocial care (2021–2025).

Domain	Recommendation (SAHCS 2021)	Implication (2021–2025 evidence)
**1. Informed consent**	For children under 12, consent from parents/guardians with child assent; for adolescents ≥12, participatory consent involving adolescent and caregiver(s); disclose risks/benefits.	**Consistent** → Evidence supports consent as a youth-centred, affirming process. **Refine** → Emphasise neurodiversity- informed adaptations (clear structure, accessible communication).
**2. Social transition**	Affirmed names, pronouns, clothing, and gender expression should be supported when requested; processes should be discussed with families/schools.	**Consistent** → Evidence shows strong protective effects of affirmation, stability of early transitions, and harms when support is delayed. **Refine** → Emphasise that both childhood and adolescent transitions are safe when affirmed, and that additional support might be required in hostile school or community environments.
**3. Family involvement**	Family participation improves outcomes; interventions may include psychoeducation, counselling, and resource-linking for caregivers and extended family.	**Consistent** → Evidence supports family affirmation as the key protective factor. **Refine** → Emphasise psychoeducation and supportive interventions that help families move through fears/uncertainties, and the importance of proactive safety-planning if family rejection occurs.
**4. School and community engagement**	MHPs should support TGD youth in navigating schools and communities, advocate for safe and inclusive environments, and provide psychoeducation to stakeholders.	**Consistent** → Evidence reinforces the protective role of inclusive school climates, safe bathrooms, supportive teachers, and access to affirming counselling and confidential school-based spaces. Harms are linked to victimisation and exclusion rather than affirmation.
**5. Mental health assessment & support**	MHPs should assess/address co-occurring conditions (depression, anxiety, suicidality, ASD) without pathologising identity.	**Consistent** → High rates of distress are linked to victimisation/rejection, not identity. Affirming interventions show benefit. **Refine** → Explicitly recommend autism-informed practices (structured sessions, sensory adaptations) and recognise distress arising from care delays or prolonged waitlists.
**6. Affirmation & non-pathologisation**	Care must be affirming, non-pathologising, and avoid gatekeeping; centre the adolescent’s lived experience.	**Consistent** → Affirmation reduces suicidality and distress, while restrictive or “watchful waiting” approaches that withhold affirmation cause harm. **Refine** → Emphasise that non-clinical enforced delays undermine wellbeing and are inconsistent with affirming care.
**7. Enabling affirming environments**	MHPs should advocate for TGD youth’s rights, contribute to policy/institutional reforms, and promote safe environments.	**Consistent** → Supportive family, school, and community environments are strongly protective. **Refine** → Emphasise anticipating minority stress in hostile social environments and the need to address inequities faced by multiply marginalised youth.
**8. Contraindicated approaches**	Practices such as gender identity and expression change efforts (“conversion therapy”), pathologisation, or non-clinical delays in care are harmful and contraindicated.	**Consistent** → Evidence confirms these practices increase distress, self-harm and suicidality. **Refine** → Explicitly name contraindicated practices.
**9. Equity & access**	Not explicitly addressed in the 2021 guideline.	**New** → Evidence highlights persistent inequities in affirmation and safety for youth facing intersecting forms of exclusion - including nonbinary young people and those in unsupportive families or schools. Future guidance should explicitly incorporate equity considerations.

**Table 4:** Summary of evidence and guideline implications for puberty-pausing medication (2021–2025)

Domain	Recommendation (SAHCS 2021)	Implication (2021–2025 evidence)
**1. Indications & eligibility**	Offer GnRHa from Tanner stage 2 for adolescents with persistent gender incongruence to relieve dysphoria, prevent unwanted pubertal changes, and support wellbeing.	**Consistent** → Evidence confirms reliable suppression and short-term safety. **Refine** → Refine → Earlier initiation is associated with more favourable physical and psychosocial trajectories; non-clinical delays reduce effectiveness and increase distress.
**2. Initiation & consent**	Initiate after MDT assessment confirming gender incongruence and capacity for informed consent; adolescents ≥12 may consent independently; caregiver involvement encouraged.	**Consistent** → Current MDT-based framework remains appropriate; combined-pathway evidence shows that coordinated endocrine–mental-health care supports clearer counselling and smoother decisions about timing and possible GAHT sequencing.
**3. Family / caregiver involvement**	Collaborative approach improves outcomes; lack of family support should not preclude access.	**Consistent** → Evidence shows that family support enhances wellbeing. Combined-pathway data suggest better mental-health trajectories when adolescents have family engagement during both PS and GAHT stages, underscoring the importance of family counselling where feasible.
**4. Clinical oversight**	Care should be managed by paediatric endocrinologist or trained provider using available GnRHa agents.	**Consistent** → Evidence reinforces the need for skilled oversight.
**5. Information & counselling**	Provide clear information on benefits, risks, and reversibility.	**Consistent** → Evidence supports the importance of clear, anticipatory counselling about expected effects, reversibility, and expected timing of potential subsequent GAHT.
**6. Fertility**	Counsel before initiation; early suppression can preclude gamete preservation.	**Consistent** → Evidence remains limited but coherent, and supports early, developmentally tailored counselling. Semen and oocyte cryopreservation are feasible and generally safe, but uptake is low due to procedural dysphoria, high cost, and late referral.
**7. Monitoring – Psychosocial wellbeing**	Follow-up should include psychosocial monitoring.	**Consistent** → Evidence indicates stable or improved mental health during PS, with no signals of harm. Combined-pathway findings show additional psychological benefit when progression to GAHT is timely and uninterrupted.
**8. Monitoring – Bone health & growth**	Monitor bone health and growth during follow-up.	**Consistent**→ Evidence shows transient BMD reductions (especially lumbar spine) and slowed growth during PS, with recovery after GAHT. Combined-pathway studies suggest stronger bone-density catch-up with testosterone than with oestrogen. **Refine** → Earlier pubertal stage at initiation is linked to more favourable growth and bone-density trajectories, while later initiation and prolonged GnRHa monotherapy may constrain recovery, underscoring the need for DXA monitoring where clinically indicated.
**9. Monitoring – Body composition & metabolic indicators**	Not explicit in 2021 guideline.	**Refine** → PS increases fat mass and reduces lean mass, which generally normalises after GAHT. Include anticipatory guidance on these expected changes, alongside counselling on physical activity and nutrition to support bone health and cardiometabolic wellbeing.
**10. ASD considerations**	Use concrete communication; involve ASD-experienced clinicians.	**Consistent** → No new PS-specific evidence. Maintain guidance.
**11. Equity & access**	Not explicitly addressed in 2021 guideline.	**New** → Structural barriers (delays, insurance, geographic inequities) affect timing and outcomes. Combined-pathway evidence shows delays between PS and GAHT can worsen mental-health trajectories. Add explicit guidance on equity in access, provincial provision, and appeal pathways.

**Table 5:** Summary of evidence and guideline implications for GAHT (2021–2025)

Domain	Recommendation (SAHCS 2021)	Implication (2021–2025 evidence)
**1. Indications & eligibility**	Initiate GAHT for adolescents with persistent incongruence, desire for hormonal change, and capacity for informed consent.	**Consistent** → Evidence shows GAHT reliably induces intended changes and improves wellbeing. Combined-pathway evidence shows continuity of benefit when GAHT follows timely PS.
**2. Consent**	From age 12, competent adolescents may consent independently; partially irreversible effects require careful counselling.	**Consistent** → Maintain detailed counselling. Combined-pathway data reinforces the need to discuss sequencing, timing, and irreversible features.
**3. Decision-making**	Clinicians and MHPs should confer on readiness; parental input improves outcomes.	**Consistent** → MDT approach remains appropriate. Combined-pathway evidence highlights the value of coordinated endocrine-mental-health care during transition from PS to GAHT.
**4. Timing**	Timing should be individualised, considering prior PS, growth potential, family support, and risks of delay.	**Refine** → Structural or administrative delays are associated with worsening mental-health symptoms. Combined-pathway studies indicate improved wellbeing and social functioning when progression from PS to GAHT is uninterrupted. Service planning should define and support timely access.
**5. Regimens (incl. non-binary care)**	Gradual dose escalation in younger adolescents; tailor to goals, including for non-binary youth.	**Consistent** → Goal-based titration remains appropriate. Combined-pathway data show that earlier PS may shape later outcomes (height, hip geometry). Continue supporting flexible dosing for non-binary clients.
**6. Fertility**	Provide counselling before GAHT on irreversible effects and preservation options.	**Consistent** → Evidence supports early, realistic fertility counselling. Gamete preservation is often feasible when GAHT is paused for several months, but adolescent data remain limited and uptake is low, partly due to cost, procedural dysphoria, and late referral.
**7. Contraception**	GAHT is not contraceptive; discuss contraception where pregnancy is possible.	**Consistent** → Maintain guidance.
**8. Concurrent health needs**	Manage co-existing conditions alongside GAHT; do not delay access unnecessarily.	**Consistent** → Evidence supports this approach;; there is no evidence that supports delaying GAHT for common concurrent conditions.
**9. Monitoring – Bone health & growth**	Monitor bone health and growth.	**Refine** → Combined-pathway evidence shows that BMD typically declines during GnRHa and increases after GAHT, with generally favourable long-term recovery, including stronger catch-up on testosterone. Transfeminine adolescents may have persistent lumbar-spine deficits, underscoring the need for ongoing DXA monitoring, adequate oestrogen dosing, vitamin D assessment, and avoidance of prolonged suppression without progression to either GAHT or endogenous puberty, as indicated.
**10. Monitoring – Body composition & metabolic indicators**	Monitor lipids, glucose, liver enzymes, and other metabolic markers.	**Consistent** → Predictable metabolic shifts occur that are consistent with the affirmed gender and are rarely clinically significant. Combined-pathway data show no additional metabolic safety concerns when transitioning from PS to GAHT.
**11. Monitoring – Cardiovascular / thrombosis**	Include cardiovascular risk assessment.	**Consistent** → Adolescents on GAHT do not show new cardiovascular safety signals; thrombotic events are rare. Combined-pathway studies report small QTc changes and transient blood-pressure shifts that remain within normal ranges, including among youth on psychotropic medications.
**12. Monitoring – Psychosocial** **outcomes**	Monitor psychosocial wellbeing and satisfaction.	**Consistent** → Evidence demonstrates improved wellbeing and high satisfaction during GAHT. Combined-pathway studies show very high continuation, rare regret, and stable or improved mental-health trajectories from PS → GAHT.
**13. Equity & access**	Not explicit in 2021 guideline.	**New** → Structural inequities (funding, geography, age-based restrictions, service delays) shape treatment timing and outcomes. Combined-pathway findings show that delays between PS and GAHT worsen psychosocial symptoms. Add explicit equity guidance: provincial scaling, public-sector funding, telehealth access, and accountability mechanisms for wait times.

**Table 6:** Summary of evidence and guideline implications for menstrual suppression (2021–2025)

Domain	Recommendation (SAHCS 2021)	Implication (2021–2025 evidence)
**1. Indication & purpose**	Mentioned only as an option when bleeding persists on testosterone or when clients do not wish to use testosterone.	**New** → Evidence shows menstrual suppression functions as a gender-affirming intervention that reduces menstrual-related dysphoria and pain. Recognise suppression as a standalone component of gender-affirming care, not only an adjunct to testosterone.
**2. Eligible methods**	Leuprolide, medroxyprogesterone acetate, anastrazole may be considered.	**Refine** → High amenorrhoea rates and favourable safety profiles demonstrated across progestin-based methods and LARC (LNG-IUD, etonogestrel implant). Expand recommended evidence-based method options and include expected effectiveness.
**3. Counselling & informed consent**	No adolescent-specific guidance; general advice only.	**New** → Provide anticipatory, developmentally appropriate counselling on expected bleeding patterns, onset of amenorrhoea, side-effect profiles, and integration with future GAHT or reproductive-health planning. Clarify that menstrual suppression does not impair long-term fertility and may support adolescents who wish to preserve future reproductive options. Address potential dysphoria linked to procedures (e.g., pelvic exams, IUD insertion).
**4. Psychosocial outcomes**	Not addressed.	**New** → Evidence shows reductions in menstrual-related dysphoria, improved daily functioning, and high satisfaction once amenorrhoea is achieved. Highlight psychosocial benefits in the guideline.
**5. Safety & adverse events**	Not addressed.	**New** → Evidence shows generally mild, manageable adverse effects and no serious complications. Reinforce routine monitoring and anticipatory side-effect counselling.
**6. Equity & access**		**New** → Address access barriers (cost, method availability, procedural discomfort, and variable provider experience with gender-affirming menstrual suppression) to support equitable provision across public-sector services.

**Table 7:** Summary of evidence and guideline implications for adolescent GAS (2021–2025).

Domain	Recommendation (SAHCS 2021)	Implication (2021–2025 evidence)
**1. Eligibility & decision-making**	Surgical eligibility and decision-making should be determined by a multidisciplinary team, including mental-health professional(s), surgeon(s), the adolescent, and parents/legal guardians.	**Consistent** → All adolescent surgeries in the identified evidence occurred within specialist multidisciplinary services and were embedded in broader GAHC pathways. Outcomes were favourable in these settings. No evidence supports modifying multidisciplinary eligibility processes; findings reinforce the importance of team-based, longitudinal, individualised care.
**2. Indications and type of surgery in adolescence**	For adolescents, GAS is most commonly limited to masculinising chest reconstruction for those mature enough to require and benefit from surgery; other procedures are usually deferred to adulthood.	**Consistent** → Evidence in minors is almost entirely limited to masculinising chest surgery, with high satisfaction and very low regret in small, mostly single-centre cohorts with low- to very-low-certainty evidence, though point estimates for regret are consistently close to zero. Insufficient data exist to expand adolescent indications, but findings support maintaining access where clinically indicated.
**3. Preoperative requirements**	Preoperative requirements include a thorough informed-consent process and at least one referral letter from a mental-health provider familiar with gender-affirming healthcare.	**Refine** → Low complication rates, high satisfaction, and very low regret occur in settings where careful assessment and preparation are standard. At the same time, evidence of unmet need and distress from cost, waitlists, and policy restrictions suggests preventing and avoiding non-medical delays beyond those required for safe, well-informed decision-making.
**4. Consent**	Consent requires the adolescent to be over 12, sufficiently mature, cognitively capable, and legally assisted by a parent/legal guardian. Irreversibility must be emphasised.	**Consistent** → No included studies examined consent processes, and South African legal standards remain unchanged. Current guidance is appropriate, especially as surgery is typically only offered to older adolescents. Emerging evidence affirms the value of clear communication and expectation-setting.
**5. Autonomy & wellbeing**	The autonomy and well-being of the adolescent must be respected and central to decisions.	**Refine** → Improvements in dysphoria, body image, daily functioning, and social participation, alongside very low regret, underscore the importance of centring adolescent goals. Evidence that non-clinical delays contribute to distress reinforces balancing caution with respect for adolescents’ bodily autonomy through shared decision-making that actively incorporates adolescents’ stated priorities.
**6. Psychosocial support**	Comprehensive psychosocial support before and after surgery is strongly recommended; ongoing support is especially important for adolescents with neurodevelopmental or psychological challenges.	**Refine** → Surgery alleviates major sources of distress but does not address broader minority stress or structural barriers. Evidence emphasises structured, ongoing mental-health, family, peer, and school-based support to sustain gains in wellbeing while addressing ongoing minority stressors.
**.7. Post-surgical care, safety & complications**	Post-surgical care should include psychological support, physiotherapy, and peer/community support resources.	**Refine** → Available cohort and systematic-review evidence from predominantly single-centre, observational studies demonstrates low complication rates in adolescents, with serious events rare and rates comparable to, or lower than, adults or cisgender adolescents undergoing analogous procedures. Guidelines could more explicitly acknowledge this favourable safety profile while reinforcing the need for structured follow-up and ongoing psychosocial care.
**8. Equity & access**	Access is constrained by limited resources, long public waitlists, and high private-sector costs.	**Refine** → Evidence from cohort and qualitative studies shows structural barriers (cost, geography, waitlists and policy restrictions) drive treatment delays, unmet need and psychological distress. Guidelines could incorporate explicit equity-focused recommendations: reducing non-clinical delays, scaling public-sector capacity, transparent waitlist management, and advocacy for funding and rights-based accountability.

**Table 8.** Summary of Evidence and Guideline Implications for Nonmedical Gender-Affirming Practices (Binding, Tucking, Packing, Padding)

Domain	Recommendation (SAHCS 2021)	Implication (2021–2025 evidence)
**1. Awareness and Engagement**	Recognise that binding, tucking, packing and padding are common gender-affirming practices and create a safe and comfortable environment in which clients can raise concerns.	**Refine** → Ensure youth are specifically mentioned. Providers should be ready to initiate open dialogue, normalise these practices, and guide clients towards safer practices when warranted.
**2. Clinical Sensitivity**	Be aware of the potential presence of specialised garments and prostheses during physical examinations, and sensitive to their purpose.	**Refine** → Ensure youth are specifically mentioned. Recognise the documented mental health benefits for youth of these practices, including reduced misgendering and improved social comfort.
**3. Safer practices: Binding**	Help patients reduce negative outcomes associated with binding by recommending ‘off-days’ from binding when possible, practising good skin hygiene, and avoiding elastic bandages, duct tape and plastic wrap as methods for binding.	**Refine** → Ensure youth are specifically mentioned. Recognise that patients may prioritise emotional benefits over physical discomfort, and support safer practice. Recognise signs of possible chest, back or rib injury and, if needed, guide clients towards safer practices with compassion and sensitivity. Support access to endocrine and/or surgical GAHC where indicated.
**4. Safer practices: Tucking**	Advise safer ways of tucking to relieve pain, such as shorter periods of tucking, less tight tucking, good skin hygiene and skin safe materials.	**Refine** → Ensure youth are specifically mentioned. As with binding, recognise that patients who tuck may prioritise emotional benefits over physical discomfort, and support safer practice with compassion and sensitivity. Consider advising on proper hydration and urinating before taping. Support access to endocrine and/or surgical GAHC where indicated.
**5. Access and equity**	*Not included in the 2021 guideline*.	**New** → Specialised undergarments and specialised tape to support safer binding and tucking exist, but commercial availability in South Africa is historically limited, erratic, and often prohibitively expensive. Clinicians can help advocate for wider availability of these products. Some TGD people, including youth, bind or tuck even when painful or injurious because they lack access to endocrine or surgical GAHC. Expanding and facilitating wider access to GAHC is beneficial.

## Box 1. Other relevant local guidelines and statements reinforcing affirming, rights-based care

In addition to the SAHCS GAHC Guideline (2021), several South African and regional professional and civil society guidelines, toolkits and statements strengthen the commitment to affirming, patient-centred and rights-based care for TGD youth. These include:

- Psychological Society of South Africa (PsySSA) Practice Guidelines for Psychology Professionals Working with Sexually and Gender-Diverse People, 2nd Edition (2025)
- Toolkit for Mental Health Providers Working with Survivors of Conversion Practices in Africa (2025)
- Embracing Diversity, Upholding Rights: A South African Position Statement on Evidence-Based Care For Transgender and Gender-Diverse Young People (2024)
- South African Society of Psychiatrists (SASOP) Child and Adolescent Psychiatry Special Interest Group Position Statement on the Care of Transgender and Non-Binary Youth (2024)
- Johannesburg Declaration Against SOGIE Change Efforts and Conversion Practices (2023)

## Appendix 1: Glossary of terms

**Table:**

Term	Definition
**Affirmative practice / affirmative stance**	Approaches that are ethical, respectful, empathic, non-judgmental, and comprehensive in understanding sexual and gender diversity, aligned with human-rights-based healthcare.
**Affirmed gender**	The gender with which an individual identifies and lives, regardless of legal or biological assignment.
**AMAB / AFAB**	“Assigned male at birth” / “assigned female at birth”; describes a legal sex designation made at birth, not the person’s gender identity.
**Batho Pele**	A South African public-service principle meaning “People First.” Emphasises dignity, respect, fairness, and accountability in service delivery, guiding ethical and person-centred healthcare.
**Binding**	The practice of flattening chest tissue using specialised compression garments or other materials to achieve a more masculinised or gender-affirming chest shape.
**Bottom surgery**	Shorthand used by many TGD people for gender-affirming genital procedures (e.g., phalloplasty, metoidioplasty, vaginoplasty) to align anatomy with gender identity.
**Chosen name / affirmed name**	The name that aligns with a person’s gender identity and replaces their birth-assigned name (see deadname); a core aspect of social affirmation.
**Cisgender**	People whose current gender identity corresponds to the sex assigned at birth.
**Cisnormativity**	The assumption that being cisgender is the default or “normal,” marginalising trans and non-binary identities as deviant or less desirable.
**Coming out**	Voluntary disclosure of one’s sexual orientation and/or gender identity to self or others; typically an ongoing process across different contexts.
**Conversion practices**	Discredited attempts to change or suppress a person’s sexual orientation, gender identity, or gender expression. Internationally known as ‘conversion therapy’ or SOGIE change efforts.
**Depathologisation**	Removal of sexual and gender diversity from psychiatric classification. Reflected in DSM-5 (“Gender Dysphoria”) and ICD-11 (“Gender Incongruence”) reclassifications outside the mental-disorders chapter.
**Detransition / retransition**	Detransition = discontinuing or reversing aspects of a previous transition. Retransition = later change to another gender identity or presentation. Neither invalidates identity or prior experience. Current evidence shows detransition is rare and most often due to external pressures (stigma, safety concerns) rather than regret.
**DSM-5 / DSM-5-TR**	DSM-5 (2013) replaced “Gender Identity Disorder” with “Gender Dysphoria.” DSM-5-TR (2022) retains the term and aligns terminology with ICD-11 “Gender Incongruence.”
**Fertility preservation**	Options offered before or during transition (e.g., gamete or tissue storage) to maintain future biological parenting potential.
**Gatekeeping**	Requiring unnecessary external approvals before offering gender-affirming care; often criticised as restrictive and unethical.
**Gender**	Identity, expression, and/or social role shaped by culture; includes cisgender, transgender, non-binary, gender-fluid, agender, etc.
**Gender affirmation / gender-affirming care**	Social, psychological, medical, and legal support aligning lived experience with gender identity; grounded in informed consent.
**Gender-affirmation surgery (GAS)**	Surgical procedures to alter primary and/or secondary sex characteristics to affirm gender identity; includes top and bottom surgeries.
**Gender-affirming hormone therapy (GAHT)**	Use of hormones (e.g., oestrogen, testosterone) to induce physical traits consistent with affirmed gender.
**Gender binary**	Belief that only two genders exist (male/female); underpins many exclusionary norms.
**Gender diverse / gender diversity**	Umbrella term for identities and expressions differing from expectations tied to sex assigned at birth. *See also* **trans/transgender**.
**Gender dysphoria**	Distress that may occur when gender identity differs from sex assigned at birth or societal expectations. In DSM-5, a diagnosis only when distress is clinically significant.
**Gender distress**	A non-clinical term increasingly used in anti-trans or sceptical policy discourse; conflates distress from incongruence with distress from stigma. Not recognised in DSM-5-TR or ICD-11.
**Gender euphoria**	Positive feelings arising from alignment between one’s gender identity and gender expression, embodiment, or social recognition. A key indicator of wellbeing.
**Gender expression**	External presentation of gender through appearance, behaviour, or voice; may or may not align with identity due to safety or social pressures.
**Gender exploratory therapy**	Approach claiming neutrality but typically assumes trans identity arises from pathology; widely criticised for mirroring conversion practices.
**Gender fluid**	A gender identity that shifts over time or context.
**Gender identity**	A person’s deeply felt, internal sense of their own gender.
**Gender incongruence (ICD-11)**	ICD-11 diagnostic category: marked, persistent incompatibility between experienced gender and sex assigned at birth; not classified as a mental disorder.
**Gender marker**	The gender designation on legal documents; may be changed through legal gender recognition processes.
**Gender modality**	How one’s gender identity relates to sex assigned at birth: cisgender, transgender, or non-binary/gender-diverse.
**Gender non-conforming**	Expression or identity not aligned with societal expectations of masculinity or femininity.
**Human-rights-based care**	A clinical and ethical approach grounded in constitutional rights to dignity, equality, bodily autonomy, and access to healthcare. Ensures that care is delivered without discrimination.
**Informed consent model**	A healthcare model in which capable individuals can access gender-affirming care without unnecessary psychiatric gatekeeping, focusing instead on patient autonomy and informed decision-making.
**Intersex**	People born with sex characteristics that do not fit typical male or female definitions; community term often preferred to “DSD.”
**Legal gender recognition (LGR)**	Processes that allow individuals to change the gender marker on official documents. Access and requirements vary by jurisdiction.
**Minority stress**	Chronic stress experienced by marginalised groups due to stigma, discrimination, and social exclusion. Strongly associated with mental-health outcomes among TGD youth.
**Misgender / misgendering**	Using pronouns or forms of address that do not reflect a person’s gender identity.
**Non-binary / enby (NB)**	Gender identities outside the male–female binary.
**Outing**	Revealing someone’s gender identity or sexual orientation without consent.
**Packing**	Use of a prosthetic or soft filler worn under clothing to create the appearance of a penis or masculine bulge.
**Padding**	Use of prosthetics or fillers to create the appearance of breasts, hips, or other gender-affirming contours.
**Passing**	Being perceived by others as the gender one identifies with.
**Pathologisation**	Treating gender diversity or neurodivergence as disorders rather than natural human variation.
**Pronouns and gendered language**	“Pronoun” is a grammatical term for a word or component of a word that can substitute for a name or a noun. Pronouns can refer either to people talking to each other (e.g. *I, you, ngi-, u-, ni-*) or to someone or something else (e.g. *she, he, this, that, u-, i-, leli-, nayi-*). In some languages, including English and Afrikaans, it is important to refer to people using pronouns that correctly reflect their gender identity (e.g. *she/her, sy/haar, he/him, hy/sy, they/them*); using correct pronouns is these languages is basic respect. In many South African languages, the pronouns used when talking about one person are the same regardless of gender (e.g. *yena*, *u-*), but it is still important to respect how each person wants to be called (e.g. *sesi, bhuti*).
**Puberty pausers / puberty blockers (GnRH agonists)**	Medications that temporarily pause puberty to prevent unwanted secondary sex characteristics and allow time for identity exploration.
**Resilience**	Psychological strength and adaptive coping in the face of stigma or discrimination.
**Secondary sex characteristics**	Physical traits emerging at puberty; do not determine gender identity.
**Social transition / social affirmation**	Changes in name, pronouns, clothing, and social recognition to align with gender identity.
**Top surgery**	Shorthand used by many TGD people for gender-affirming chest surgery — mastectomy with contouring (transmasculine) or breast augmentation (transfeminine).
**Trans / transgender**	Umbrella terms for people whose gender identity or expression differs from that expected for sex assigned at birth; including transgender, non-binary, gender-fluid, genderqueer, agender, as well as culturally specific and/or precolonial indigenous identities such as hijra (parts of South Asia), Two Spirit (some Indigenous people in North America), muxe (Zapotec people in Mexico), brotherboy/sistergirl (some Australian Aboriginal and Torres Strait Islander cultures) and many others.
**Trans feminine (trans femme)**	AMAB person who identifies with or expresses femininity.
**Trans masculine (trans masc)**	AFAB person who identifies with or expresses masculinity.
**Trans men / men of trans experience**	People who identify as men and were AFAB; older terms like FTM are outdated.
**Trans women / women of trans experience**	People who identify as women and were AMAB; older terms like MTF are outdated.
**Transnormativity**	Social expectation that “acceptable” trans identity follows a narrow script (binary, early childhood certainty, medical transition).
**Transphobia**	Negative attitudes, beliefs, or actions toward transgender people.
**Transition / transitioning**	Social, medical, and/or legal processes aligning lived gender with identity.
**Tucking**	Practice used by some AMAB people to create a flatter groin profile, using garments or tape.
**Ubuntu**	A Southern African philosophy emphasising relationality, interconnectedness, and collective responsibility. In healthcare, it highlights that wellbeing is supported through caring and reciprocal relationships within families, communities, and society.

## Appendix 2: PRISMA 2020 Checklist

Rapid Review of Gender-Affirming Healthcare for Children and Adolescents: Evidence Synthesis (2021–2025) and Recommendations for South Africa (Version 1.0 2025-12-01)

**Table:**

Section and Topic	Item #	Checklist item	Location where item is reported
**TITLE**			
Title	1	Identify the report as a systematic review. *It’s a rapid review, but it is in the title*	Title page
**ABSTRACT**			
Abstract	2	See the PRISMA 2020 for Abstracts checklist.	Technical Summary
**INTRODUCTION**			
Rationale	3	Describe the rationale for the review in the context of existing knowledge.	1.1 Rationale for this rapid review
Objectives	4	Provide an explicit statement of the objective(s) or question(s) the review addresses.	1.2 Purpose and research questions
**METHODS**			
Eligibility criteria	5	Specify the inclusion and exclusion criteria for the review and how studies were grouped for the syntheses.	3.1 Eligibility criteria
Information sources	6	Specify all databases, registers, websites, organisations, reference lists and other sources searched or consulted to identify studies. Specify the date when each source was last searched or consulted.	3.2 Search strategy, screening and selection
Search strategy	7	Present the full search strategies for all databases, registers and websites, including any filters and limits used.	3.2 Search strategy, screening and selection
Selection process	8	Specify the methods used to decide whether a study met the inclusion criteria of the review, including how many reviewers screened each record and each report retrieved, whether they worked independently, and if applicable, details of automation tools used in the process.	3.2 Search strategy, screening and selection
Data collection process	9	Specify the methods used to collect data from reports, including how many reviewers collected data from each report, whether they worked independently, any processes for obtaining or confirming data from study investigators, and if applicable, details of automation tools used in the process.	3.3. Data extraction and Analysis
Data items	10a	List and define all outcomes for which data were sought. Specify whether all results that were compatible with each outcome domain in each study were sought (e.g. for all measures, time points, analyses), and if not, the methods used to decide which results to collect.	3.3. Data extraction and Analysis
	10b	List and define all other variables for which data were sought (e.g. participant and intervention characteristics, funding sources). Describe any assumptions made about any missing or unclear information.	3.3. Data extraction and Analysis
Study risk of bias assessment	11	Specify the methods used to assess risk of bias in the included studies, including details of the tool(s) used, how many reviewers assessed each study and whether they worked independently, and if applicable, details of automation tools used in the process.	Described under “Inclusion of original research reports in previous systematic reviews” within 3.3. Data extraction and Analysis
Effect measures	12	Specify for each outcome the effect measure(s) (e.g. risk ratio, mean difference) used in the synthesis or presentation of results.	Due to diverse interventions and outcomes, these measures, where available, are presented in results subsections 4.1.2 Findings from primary studies; 4.2.2 Puberty-pausing medication; 4.2.3 Gender-affirming hormone therapy; 4.2.4 Combined endocrine treatment pathways; 4.2.5 Menstrual suppression as gender-affirming care; 4.2.6 Fertility counselling and preservation; 4.3.2 Gender-affirming surgery.
Synthesis methods	13a	Describe the processes used to decide which studies were eligible for each synthesis (e.g. tabulating the study intervention characteristics and comparing against the planned groups for each synthesis (item #5)).	3.4 Narrative synthesis of findings; 4.1.4 Psychosocial care findings synthesis and guideline implications; 4.2.6 Synthesis of endocrine evidence and guideline implications; 4.3.5 Synthesis of surgical-care findings and guideline implications; 4.4 Synthesis and Implications for Guidelines; 4.5.5 Policy evidence synthesis and guideline implications.
	13b	Describe any methods required to prepare the data for presentation or synthesis, such as handling of missing summary statistics, or data conversions.	3.3 Data extraction and Analysis; 3.4 Narrative synthesis of findings.
	13c	Describe any methods used to tabulate or visually display results of individual studies and syntheses.	3.3 Data extraction and Analysis (description of Airtable-based tabulation); 4.x Results tables within each domain (psychosocial, endocrine, surgical, non-medical, policy).
	13d	Describe any methods used to synthesize results and provide a rationale for the choice(s). If meta-analysis was performed, describe the model(s), method(s) to identify the presence and extent of statistical heterogeneity, and software package(s) used.	3.4 Narrative synthesis of findings.
	13e	Describe any methods used to explore possible causes of heterogeneity among study results (e.g. subgroup analysis, meta-regression).	n/a
	13f	Describe any sensitivity analyses conducted to assess robustness of the synthesized results.	n/a
Reporting bias assessment	14	Describe any methods used to assess risk of bias due to missing results in a synthesis (arising from reporting biases).	Not done, rapid review (explicitly acknowledged in 3.5 Strengths and limitations of the review process)
Certainty assessment	15	Describe any methods used to assess certainty (or confidence) in the body of evidence for an outcome.	3.3 Data extraction and Analysis (extraction of GRADE/other certainty ratings from existing systematic reviews); 3.4 Narrative synthesis of findings (use of existing appraisals as qualitative context).
**RESULTS**			
Study selection	16a	Describe the results of the search and selection process, from the number of records identified in the search to the number of studies included in the review, ideally using a flow diagram.	Introduction to Section 4
	16b	Cite studies that might appear to meet the inclusion criteria, but which were excluded, and explain why they were excluded.	Not done. 501 reports excluded with reasons indicated in PRISMA diagram.
Study characteristics	17	Cite each included study and present its characteristics.	Results and Data Tables
Risk of bias in studies	18	Present assessments of risk of bias for each included study.	Results subsections (4.1–4.5), with process described under “Inclusion of original research reports in previous systematic reviews” within 3.3 Data extraction and Analysis
Results of individual studies	19	For all outcomes, present, for each study: (a) summary statistics for each group (where appropriate) and (b) an effect estimate and its precision (e.g. confidence/credible interval), ideally using structured tables or plots.	Results tables
Results of syntheses	20a	For each synthesis, briefly summarise the characteristics and risk of bias among contributing studies.	4.1.4 Psychosocial care findings synthesis and guideline implications; 4.2.6 Synthesis of endocrine evidence and guideline implications; 4.3.5 Synthesis of surgical-care findings and guideline implications; 4.4 Synthesis and Implications for Guidelines; 4.5.5 Policy evidence synthesis and guideline implications; 5.1 Summary of key findings
	20b	Present results of all statistical syntheses conducted. If meta-analysis was done, present for each the summary estimate and its precision (e.g. confidence/credible interval) and measures of statistical heterogeneity. If comparing groups, describe the direction of the effect.	Not done, due to extreme heterogeneity
	20c	Present results of all investigations of possible causes of heterogeneity among study results.	Not done, due to extreme heterogeneity and rapid review
	20d	Present results of all sensitivity analyses conducted to assess the robustness of the synthesized results.	Not done, due to extreme heterogeneity and rapid review
Reporting biases	21	Present assessments of risk of bias due to missing results (arising from reporting biases) for each synthesis assessed.	Not done due to rapid review
Certainty of evidence	22	Present assessments of certainty (or confidence) in the body of evidence for each outcome assessed.	3.3 Data extraction and Analysis; 3.4 Narrative synthesis of findings;withing individual sections of Chapter 4 Results and 5.2 Strengths and limitations of the current evidence base
**DISCUSSION**			
Discussion	23a	Provide a general interpretation of the results in the context of other evidence.	5.1 Summary of key findings
	23b	Discuss any limitations of the evidence included in the review.	5.3 Strengths and limitations of the current evidence base
	23c	Discuss any limitations of the review processes used.	5.2 Strengths and limitations of this rapid review
	23d	Discuss implications of the results for practice, policy, and future research.	5.4 Implications for clinical practice; 5.5 Implications for policy and health systems; 5.6 Priorities for future research
**OTHER INFORMATION**			
Registration and protocol	24a	Provide registration information for the review, including register name and registration number, or state that the review was not registered.	Methods para 2
	24b	Indicate where the review protocol can be accessed, or state that a protocol was not prepared.	Methods para 2
	24c	Describe and explain any amendments to information provided at registration or in the protocol.	n/a
Support	25	Describe sources of financial or non-financial support for the review, and the role of the funders or sponsors in the review.	Author Declarations
Competing interests	26	Declare any competing interests of review authors.	Author Declarations
Availability of data, code and other materials	27	Report which of the following are publicly available and where they can be found: template data collection forms; data extracted from included studies; data used for all analyses; analytic code; any other materials used in the review.	Not explicitly reported (underlying screening and extraction tables held in internal Airtable and Excel databases; available from authors on reasonable request)

*From:* Page MJ, McKenzie JE, Bossuyt PM, Boutron I, Hoffmann TC, Mulrow CD, et al. The PRISMA 2020 statement: an updated guideline for reporting systematic reviews. BMJ 2021;372:n71. doi: 10.1136/bmj.n71. This work is licensed under CC BY 4.0. To view a copy of this license, visit https://creativecommons.org/licenses/by/4.0/

This “Abstract” checklist applies to the “Technical Summary” in the full report

**Table:**

Section and Topic	Item #	Checklist item	Reported (Yes/No)
**TITLE**			
Title	1	Identify the report as a systematic review.	Yes
**BACKGROUND**			
Objectives	2	Provide an explicit statement of the main objective(s) or question(s) the review addresses.	Yes
**METHODS**			
Eligibility criteria	3	Specify the inclusion and exclusion criteria for the review.	Yes
Information sources	4	Specify the information sources (e.g. databases, registers) used to identify studies and the date when each was last searched.	Yes
Risk of bias	5	Specify the methods used to assess risk of bias in the included studies.	Yes
Synthesis of results	6	Specify the methods used to present and synthesise results.	Yes
**RESULTS**			
Included studies	7	Give the total number of included studies and participants and summarise relevant characteristics of studies.	Yes
Synthesis of results	8	Present results for main outcomes, preferably indicating the number of included studies and participants for each. If meta-analysis was done, report the summary estimate and confidence/credible interval. If comparing groups, indicate the direction of the effect (i.e. which group is favoured).	Yes
**DISCUSSION**			
Limitations of evidence	9	Provide a brief summary of the limitations of the evidence included in the review (e.g. study risk of bias, inconsistency and imprecision).	Yes
Interpretation	10	Provide a general interpretation of the results and important implications.	Yes
**OTHER**			
Funding	11	Specify the primary source of funding for the review.	Yes
Registration	12	Provide the register name and registration number.	Yes – stated as not

*From:* Page MJ, McKenzie JE, Bossuyt PM, Boutron I, Hoffmann TC, Mulrow CD, et al. The PRISMA 2020 statement: an updated guideline for reporting systematic reviews. BMJ 2021;372:n71. doi: 10.1136/bmj.n71. This work is licensed under CC BY 4.0. To view a copy of this license, visit https://creativecommons.org/licenses/by/4.0/

## List of abbreviations

AFAB: Assigned female at birth
AMAB: Assigned male at birth
ASAB: Assigned sex at birth
ASD: Autism spectrum disorder
DSM: Diagnostic and Statistical Manual of Mental Disorders
DSD: Differences in sex development
EtD: Evidence to Decision
GAHC: Gender-affirming healthcare
GAHT: Gender-affirming hormone therapy
GRADE: Grading of Recommendations Assessment, Development and Evaluation
HIV: Human Immunodeficiency Virus
HIVSS: HIV self-screening
ICD: International Classification of Disease
LGBTQAI+: Lesbian, Gay, Bisexual, Transgender, Queer, Intersex, Asexual and others
NSP: National Strategic Plan (HIV, TB & STIs)
PATHSA: Professional Association for Transgender Health South Africa
PEP: Post-exposure prophylaxis
PrEP: Pre-exposure prophylaxis
PRISMA: Preferred Reporting Items for Systematic Reviews and Meta-Analyses
PROSPERO: International Prospective Register of Systematic Reviews
PsySSA: Psychological Society of South Africa
SA: South Africa
SoC-8: Standards of Care, Version 8
SOGIE: Sexual Orientation and Gender Identity and Gender Expression
SRHR: Sexual and Reproductive Health and Rights
STI: Sexually transmitted infection
TGD: Transgender and gender diverse
WHO: World Health Organization
